# Design, Facile Synthesis and Characterization of Dichloro Substituted Chalcones and Dihydropyrazole Derivatives for Their Antifungal, Antitubercular and Antiproliferative Activities

**DOI:** 10.3390/molecules25143188

**Published:** 2020-07-13

**Authors:** Afzal B. Shaik, Richie R. Bhandare, Srinath Nissankararao, Zehra Edis, N. Ravikiran Tangirala, Shaik Shahanaaz, M. Mukhlesur Rahman

**Affiliations:** 1 Department of Pharmaceutical Chemistry, Vignan Pharmacy College, Jawaharlal Nehru Technological University, Vadlamudi 522 213, India; 2 Department of Pharmaceutical Sciences, College of Pharmacy & Health Sciences, Ajman University, Ajman PO Box 346, UAE; z.edis@ajman.ac.ae; 3 Independent Researcher, Montvale, NJ 07645, USA; srinath.n2k@gmail.com; 4 Department of Pharmaceutical Chemistry, Narasaraopeta Institute of Pharmaceutical Sciences, Jawaharlal Nehru Technological University, Narsaraopet 522 601, India; ravikirannaga58@gmail.com; 5 Department of Pharmaceutical Chemistry, Victoria College of Pharmacy, Nallapadu, Guntur District 522 001, India; shahanaazchemistry@gmail.com; 6 Medicines Research Group, School of Health, Sports and Bioscience, University of East London, Stratford Campus, Water Lane, London E15 4LZ, UK

**Keywords:** chalcone, dihydropyrazole, antifungal, antitubercular, antiproliferative activity

## Abstract

Infectious diseases caused by fungi and mycobacteria pose an important problem for humankind. Similarly, cancer is one of the leading causes of death globally. Therefore, there is an urgent need for the development of novel agents to combat the deadly problems of cancer, tuberculosis, and also fungal infections. Hence, in the present study, we designed, synthesized, and characterized 30 compounds including 15 chalcones (**2**–**16**) and 15 dihydropyrazoles (**17**–**31**) containing dichlorophenyl moiety and also screened these compounds for their antifungal, antitubercular, and antiproliferative activities. Among these compounds, the dihydropyrazoles showed excellent antifungal and antitubercular activities whereas the chalcones exhibited promising antiproliferative activity. Among the dihydropyrazoles, compound **31** containing 2-thienyl moiety showed promising antifungal activity (MIC 5.35 µM), whereas compounds **22** and **24** containing 2,4-difluorophenyl and 4-trifluoromethyl scaffolds revealed significant antitubercular activity with the MICs of 3.96 and 3.67 µM, respectively. Compound **16** containing 2-thienyl moiety in the chalcone series showed the highest anti-proliferative activity with an IC_50_ value of 17 ± 1 µM. The most active compounds identified through this study could be considered as starting points in the development of drugs with potential antifungal, antitubercular, and antiproliferative activities.

## 1. Introduction

**Figure 1:**
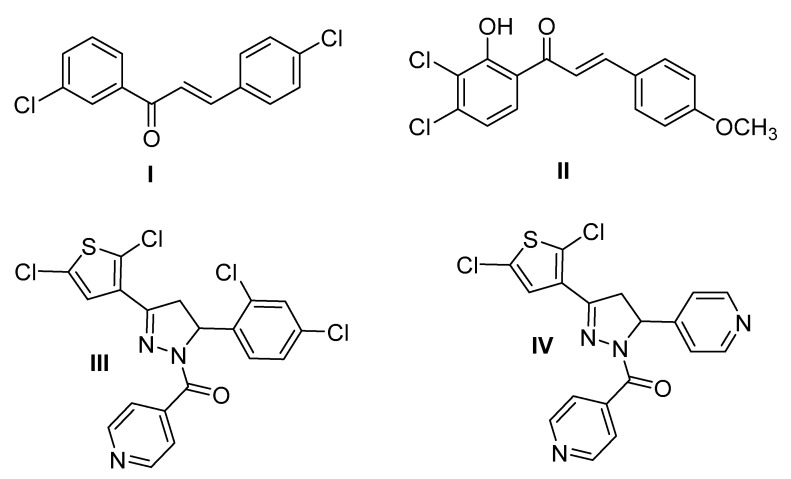
Structures of chlorinated and heteroaryl-based chalcones and dihydropyrazoles with potential biological activities.

Chalcones are a class of natural products characterized by the presence of two aromatic rings connected through α,β-unsaturated ketone moiety. Hence, these are a type of 1,3-diphenyl-2-propene-1-ones which serve as precursors for the synthesis of different classes of flavonoids and isoflavonoids—a class of biologically important of natural products. The α,β-unsaturated ketone moiety is highly reactive, making the chalcones valuable starting materials for the synthesis of a range of heterocyclic compounds of ring size of 5, 6, or 7 with nitrogen, oxygen, and sulfur as hetero atoms [1]. Chalcone derivatives, which are excellent starting points in drug design [2,3,4,5,6,7], are well-known for varied biological activities such as antimicrobial, anticancer, antimalarial, antioxidant, anti-tubercular, anti-inflammatory, and anti-tumor activities [8,9,10,11,12,13,14,15]. Dihydropyrazole is an interesting heterocyclic compound, which can be synthesized from chalcones. Compounds containing dihydropyrazole skeletons have been reported to possess varied activities such as antibacterial, antifungal, anticancer, analgesic, and anti-inflammatory activities [16,17,18,19,20]. We have previously reported various phenyl-substituted and heterocycle-based chalcones and dihydropyrazoles with significant antioxidant, antibacterial, antifungal, antitubercular, cytotoxic, and anticancer activities [21,22,23,24,25,26,27,28]. Previous reports published in the literature suggest that the presence of chalcones (Figure 1 I,II) and dihydropyrazoles (Figure 1 III,IV) substituted with chlorine and other substituents such as heterocycle-based chalcones and dihydropyrazole derivatives are essential structural features for biological activities including antifungal, antibacterial, anticancer, and antioxidant activities [2,5,15,29,30,31,32,33]. For example, chlorinated chalcones I and II showed significant antibacterial activities against both Gram-positive (*Staphylococcus aureus* and *Bacillus subtilis*) and Gram-negative bacteria (*Pseudomonas aeruginosa* and *E. coli*) and antifungal activity against *Candida albicans*, *Aspergillus niger* and *Penicillium chrysogenum* [4]. The understanding of chlorine’s affect on biological activity was further facilitated by data generated through computational analysis [34] which suggested that the presence of chlorine atoms along with another electrophilic substituents on the aromatic ring retains/enhances biological activities such as antimicrobial properties [34].

**Figure 2:**
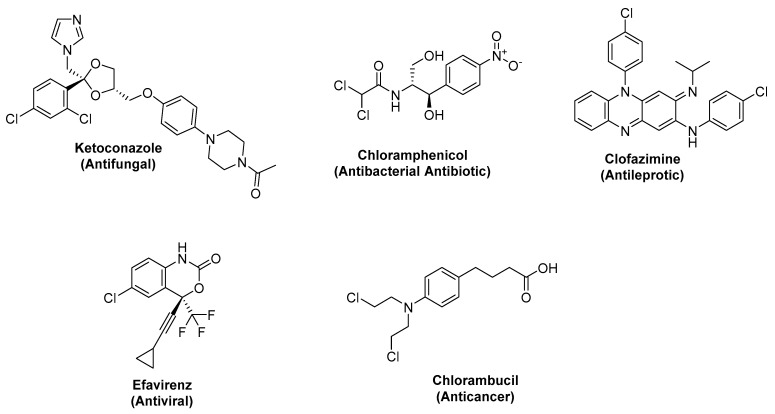
Structures of selected drugs containing one or more chlorine atoms.

The presence of chlorine as a substituent in many drugs appears to be responsible for bioactivity. Fang et al. [35] reported that a total of 163 compounds out of the 233 drugs approved for clinical uses contain chlorine in their structures. Among the 163 compounds, nearly 73% of them possess one chlorine atom whilst 23%, 2.6%, 1.4%, and 2.5% of such compounds were found to have two, three, four, and six chlorine atoms in their molecular structures, respectively [35]. The current market for chlorine containing drugs is worth approximately USD 168.5 billion, of which anti-infectives and cytostatics account for USD 9.5 and 12.7 billion, respectively [36]. The presence of one or more chlorine atoms is the prominent feature in antibiotic, antifungal, antileprotic, antiviral, and anticancer agents (Figure 2).

**Figure 3:**
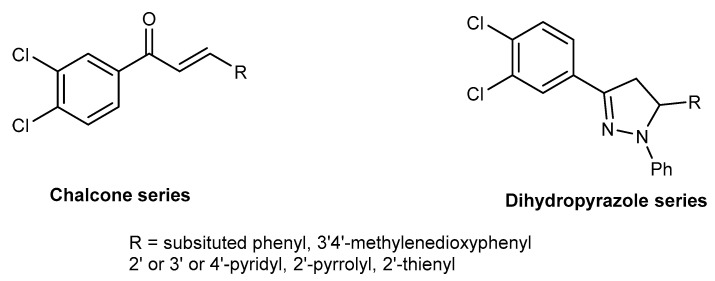
Newly designed chlorine containing chalcones and dihydropyrazole derivatives.

The term “mycos”, as in the term “mycology” (the study of fungi), means waxy, which indicates that the cell walls of fungal and mycobacterial microorganisms are hydrophobic in nature. Therefore, lipophilicity is an important concern for designing molecules to act as better antifungal and antitubercular agents. It was observed that the incorporation of a chlorine substituent in one or the other specific locations of the pharmacologically active molecules improved their inherent bioactivity [37]. The presence of the chlorine atom as a substituent improves the lipophilicity of the molecule and in turn favors the greater partitioning of the chlorinated compounds into the lipophilic component of the cell membranes or the lipophilic domains of the protein molecule. This leads to a higher concentration of the molecule at the site of action and may result in improved bioactivity. The chlorine atom present in the alkyl chain is highly reactive and readily undergoes nucleophilic aliphatic substitution reaction in the body [38]. The chlorine present as a substituent on an aromatic or heteroaromatic ring or olefinic fragment is non-reactive, hence it will be carried by the molecule to the site of action where it may help in the efficient binding of the drug molecule with the target through the electronic and/or steric effects of chlorine [38]. In addition to its lipophilicity and membrane penetrability, another important feature of the presence of the chlorine atom in molecules is its ability to form halogen bonding. The participation of halogen bonding is evident in ligand–receptor interactions due to its unique ability to act as both Lewis acid and Lewis base [39,40]. Hence, the presence of the chlorine atoms in the chemical structure will enhance the required lipophilicity, aid in the penetration of the molecule, facilitate halogen bonding and in turn help to increase bioactivity. Halogen bonding plays the same important role in polyiodides related to antimicrobial activities and lipophilicity [41,42,43]. As a part of our ongoing research as well as based on the information published in the literature and our previously reported work, here we report the design and facile synthesis of dichloro-substituted chalcones and dihydropyrazoles (Figure 3) for their antifungal, anti-tubercular and antiproliferative activities.

## 2. Results and Discussion

### 2.1. Chemistry

**Scheme 1:**
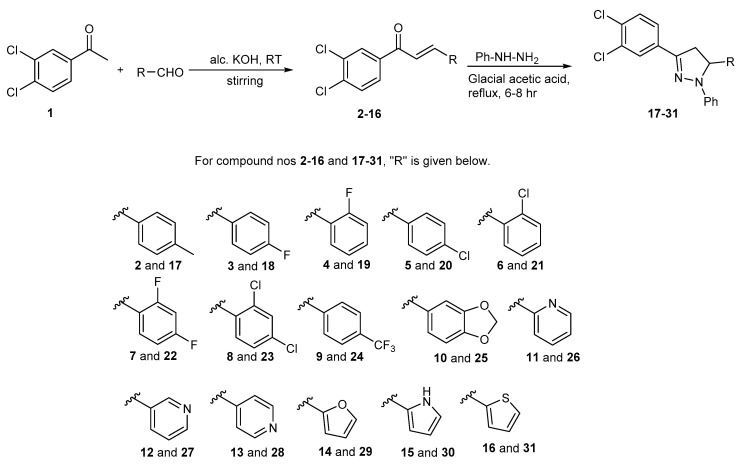
Protocol for the synthesis of dichlorophenyl rings containing chalcones and dihydropyrazole derivatives; RT: room temperature.

Chalcones (**2**–**16**) were prepared by a Claisen–Schmidt condensation reaction in the presence of alcoholic potassium hydroxide as a catalyst. The 3,4-dichloroacetophenone (**1**) was condensed with substituted benzaldehyde by stirring under basic conditions at room temperature for 24 h followed by transferring the mixture to a beaker containing crushed ice. The crude precipitate was recrystallized using ethanol to afford chalcones **2**–**16** (Scheme 1). The target dihydropyrazoles **17**–**31** were obtained by refluxing the synthesized chalcones with phenyl hydrazine for 6–8 h in glacial acetic acid and purified using silica gel column chromatography (Scheme 1). The synthesized compounds **2** [44], **3** [45], **4**–**6** [44], **10** [46], and **13** [47] were characterized by various spectroscopic methods including FTIR, ^1^H-NMR, ^13^C-NMR, and MS. (Spectral data are provided in the supplementary material).

All the synthesized chalcones exhibited characteristic absorption bands in the FTIR spectra (cm^−1^) in the ranges 1640–1660 (C=O), 1570–1605 (C=C quadrant of Ar), and 1500–1550 (HC=CH), and at other regions of the spectrum depending upon the specific substituents present in each compound. The ^1^H-NMR spectrum (400 MHz, CDCl_3_) of the chalcones revealed the characteristic ethylenic protons of the α,β-unsaturated ketone system between δ 6.85 and 8.10 ppm as two doublets. A higher chemical shift value at 8.10 ppm was observed for the protons beta to the carbonyl group. The spectra also showed the peaks accounting for the aromatic protons and for the different substituent protons between the corresponding regions of the spectrum.

Compound **17** was analyzed for C_22_H_18_Cl_2_N_2_, melting point (m.p.) 168 °C, well supported by its molecular ion [M]^+^ that appeared at *m*/*z* 381.30 in its mass spectrum. The spectrum also showed an isotope satellite signal (^37^Cl) with one-third intensity at *m*/*z* 383.30. The FTIR spectrum (KBr disc) showed characteristic intense absorption bands at 1592 (C=N), 1120 (C-N), 1586 (C=C quadrant of Ar), and 830 (C-Cl) cm^−1^. The ^1^H-NMR spectrum (400 MHz, CDCl_3_) showed the characteristic HA, HB, and HX protons at δ 3.28, 3.72, and 5.45, respectively, as doublet of doublets (dd) with *J*_AB_ = 16.95 Hz, *J*_AX_ = 7.50 Hz and *J*_BX_ = 9.35 Hz respectively. The spectrum also accounted for all 12 aromatic protons that appeared in the range δ 6.75–7.91. The ^13^C-NMR spectrum (100 MHz, CDCl_3_) showed all the carbon resonances characteristic of the above compound at δ 152.50 (C-3), 66.54 (C-5), 45.50 (C-4), 133.95 (C-2′ and C-2″), 143.51 (C-3′ and C-3″), 134.88 (C-4′ and C-4″), and in the range δ 112.12–145.96 for all the carbons of the phenyl ring. Based on the above spectral data, the structure of compound **17** was confirmed as 3-(3,4-dichlorophenyl)-1-phenyl-5-(p-tolyl)-4,5-dihydro-1H-pyrazole.

### 2.2. Biological Studies

All the compounds synthesized through this study were evaluated for their antifungal, antitubercular, and antiproliferative activities employing standard protocols. The antiproliferative and cytotoxic studies on the synthesized compounds were carried out at Chemiloids Life Sciences Pvt Limited, Vijayawada, Andhra Pradesh, India. The compounds were sorted into three different categories based on their structural features. For instance, compounds **2**–**6**, **9**, **17**–**21**, and **24** represented monosubstituted chalcones and dihydropyrazoles, whereas compounds **7**, **8**, **22**, and **23** represented disubstituted chalcones and dihydropyrazoles, and compounds **10**–**16** and **25**–**31** represented the bioisosteric substitution of a phenyl ring.

#### 2.2.1. Antifungal Activities

In the monosubstituted chalcone series (Table 1) **2**–**6** and **9**, the para and ortho position were substituted by either CH_3_ (**2**), F (**3, 4**), or Cl (**5**, **6**) groups. The chalcone (**2**) with methyl substituent showed the lowest antifungal activity with an MIC of 109.9 µM. Replacing methyl (CH_3_) substituent (**2**) with fluorine (F) (**3**) or chlorine (Cl) (**5**) resulted in two- and fourfold improvement in activity against *Aspergillus niger*, respectively, but twofold improvement against *Candida tropicalis* in the case of both compounds. Changing the position of F from para to ortho (**3** vs **4**) resulted in twofold improvement in activity against *Aspergillus niger* but no change was observed against *Candida tropicalis*. At the ortho position, when F was substituted with Cl (**4** vs. **6**), no change in activity was observed against *Aspergillus niger* and *Candida tropicalis*. When the CH_3_ group at the para position (**2**) was replaced with a highly electron-withdrawing group such as CF_3_ (**9**), it resulted in twofold and 1.6 fold improvement in activity of 11.58 µM against *Aspergillus niger* and *Candida tropicalis*, in comparison to the reference drug fluconazole (MIC 26.11 and 19.58 µM against *Aspergillus niger* and *Candida tropicalis*), which suggests that a highly electron-withdrawing group is suitable at this position.

In the disubstituted chalcones **7** and **8**, substituting another F or Cl on the para position of compounds **4** and **6** resulted in no change in activity. In the bioisosteric chalcone series, substituting a phenyl ring with methylene dioxyphenyl (**10**) resulted in MIC of 23 µM against *Aspergillus niger* but poor activity against *Candida tropicalis* (MIC 180 µM). Among the six-membered heterocycles **11** to **13**, compound **12** demonstrated better MIC (14.43 µM) against *Candida tropicalis*. It also appeared that nitrogen at position 3 on the pyridine ring (**12**) showed better activity against positions 2 (**11**) and 4 (**13**). Among the five-membered bioisostere-based compounds, substituting chalcones **14** to **16** with the 2-thienyl group (**16**) exhibited the best antifungal activity of 14.12 µM. Other bioisosteres, such as 2-furfuryl (**14**) and 2-pyrrolyl (**15**), showed low and modest activity (MICs 234.97, 117.48, and 60.37 µM). Compound **16** with the 2-thienyl group was found to be similar in activity (MIC 14.12 µM) to compound **9** (MIC 11.58 µM) in comparison with other mono- and disubstituted phenyls and six-membered heterocyclic rings containing chalcones. The most potent among the heterocycle-based chalcone series was compound **16**, with 1.84- and 1.35-fold better activity against *Aspergillus niger* and *Candida tropicalis* in comparison to fluconazole.

The dihydropyrazole series fared better than the chalcones. The overall antifungal activity of the dihydropyrazole series ranged from 5.35–41.96 µM. In the monosubstituted series **17**–**21** and **24**, antifungal activity ranged from 9.18–41.96 µM against *Aspergillus niger* and *Candida tropicalis*. Para substitution with CH_3_ (**17**) gave the lowest activity among the monosubstituted series with an MIC of 41.96 µM. Replacing CH_3_ with the highly electronegative substituent CF_3_ (**24**) caused 4.6-fold improvement in activity against both species suggesting that an electron-withdrawing group at the para position is favored for activity. When the CH_3_ group was replaced with either F (**18**) or Cl (**20**), antifungal activity was improved twofold to 28.76 µM against *Aspergillus niger*. Substitution at the ortho position with F (**19**) or Cl (**21**) showed identical MICs with the para position having F (**18**) and Cl (**21**) (20.76 and 19.91 µg/mL for *Aspergillus niger* and 41.53 and 39.82 µM for *Candida tropicalis*, respectively). In the monosubstituted chalcone series, compound **24** fared 2.84- and twofold better in activity over fluconazole against *Aspergillus niger* and *Candida tropicalis*.

In the disubstituted compounds **22** and **23**, F and Cl substitution at ortho and para did not show any improvement in antifungal activity in comparison to compounds **18**–**21**. Compound **22** only showed twofold improvement in activity (MIC 19.83 µM) against *Candida tropicalis* in comparison to compounds **18** and **19**. Among the bioisosteres (**25**–**31**), the activity ranged from 5.35–44.91 µM. The bioisostere 3,4-methylenedioxyphenyl (**25**) had an MIC of 19.45 µM against *Aspergillus niger* and *Candida tropicalis*, similar to standard fluconazole. Substitution on the pyridine ring at the 2′, 3′, or 4′ position (**26**–**28**) did not alter antifungal activity (MIC 21.72 µM). In the five-membered heterocycles (**29**–**31**), the best activity was observed for compound **31** (MIC 5.35 µM) and it was found to be 4.9 and 3.7-fold better in activity over standard fluconazole against *Aspergillus niger* and *Candida tropicalis*.

In comparison to chalcone **9**, the monosubstituted series containing dihydropyrazole **24** showed similar MICs of 11.58 and 9.18 µM against both fungi. However, in the five-membered heterocycle, dihydropyrazoles fared better than chalcones, showing 2.6-fold improvement in MIC (**31**, 5.35 µM vs **16**, 14.12 µM) indicating that antifungal activity proceeded better in dihydropyrazoles than in chalcones. 

#### 2.2.2. Antitubercular Activity

In the monosubstituted chalcone series (Table 2), substitution with the CH_3_ (**2**) group at the para position of the phenyl ring resulted in the lowest activity of 343.44 µM. When CH_3_ was replaced with F or Cl, it showed fourfold improvement in activity (MIC 84.70 µM) indicating that the electronegative halogen substituent was favorable over the electron-releasing group. Hence, when CH_3_ was substituted with CF_3_, the activity was improved by approximately 40-fold for **9** (MIC 9.03 µM) vs. compound **2** (MIC 343.44 µM). Substitution with other electronegative functional groups, such as fluorine in compounds **3** and **4** at para and ortho positions, showed similar activity with an MIC of 84.70 µM. However, compound **6** containing chlorine at the ortho position (MIC 20.05 µM) showed a fourfold increase in activity compared with **5** which had chlorine at the para position (MIC 80.23 µM). Overall, in the monosubstituted series of chalcones, **9** showed 2.8-fold better activity than the standard pyrazinamide (MIC 9.03 vs. 25.34 µM).

Among the disubstituted chalcones, the incorporation of F (**7**; MIC 9.96 µM) at both ortho and para positions resulted in activity nearly equal to that of compound **9** (MIC 9.03 µM)). Replacing F (**7**) with Cl (**8**) caused a nearly twofold drop in activity. Among the bioisosteres, the MIC ranged from 22.07 to 72.24 µM. Among the six-membered heterocycles, substitution with 3-pyridyl (**12**) resulted in an MIC of 22.56 µM, an activity greater than pyrazinamide whereas compounds **11** and **13** containing 2- and 4-pyridinyl scaffolds showed half the potency of that shown by compound **12** and pyrazinamide, indicating the significance of the point of attachment on the pyridine ring. Among the five-membered heterocycles, 2-thienyl (**16**) gave the best activity at 22.07 µM and was found to be comparable with pyrazinamide. Substitution of the 2-thienyl ring with 2-furfuryl (**14**) or 2-pyrrolyl (**15**) resulted in a twofold drop in activity.

The dihydropyrazoles in most of the cases showed activity at lower MIC values than the corresponding chalcones. This designates that the dihydropyrazole derivatives are more promising antitubercular agents than the compounds belonging to the chalcone series. In the monosubstituted series, compound **17** containing the CH_3_ group was the least potent in the dihydropyrazole series with MIC 262.26 µM whereas the other compounds **18**–**21** containing fluorine and chlorine atoms showed activity ranging from 15.55 to 32.44 µM. Compound **21** containing Cl at ortho position showed 1.6 times more activity than pyrazinamide whereas compounds **18** and **19** containing F at ortho and para positions as well as **20** with Cl at para position showed 1.2-fold less activity than pyrazinamide. Compound **24** containing CF_3_ showed top-class activity with an MIC of 3.67 µM, and this was the most potent among the thirty tested compounds having sevenfold greater activity than pyrazinamide.

However, in the case of the disubstituted compounds **22** and **23**, when the ortho and para positions were substituted with F as in **22** (MIC, 3.96 µM) the activity improved eightfold. Similarly, the activity of compound **23** (MIC, 7.15 µM) bearing Cl atoms at ortho and para positions showed 4- and 2-fold improvement in activity compared to compounds **20** and **21**, respectively.

Among the bioisosteres, there was a drop in the activity of the six-membered heterocycles **26**–**28** containing pyridine attachments (2′, 3′, and 4′, MIC = 67.88 µM) compared to the corresponding chalcones **11**–**13**. Similarly, **30**, bearing a five-membered nitrogen-containing pyrrole nucleus, had a 1.5-fold reduction in activity compared with **15**. However, the other two compounds **29** and **31** containing five-membered heterocycles 2′-furyl and 2′-thienyl moieties showed 2.6-fold more activity than the chalcones **14** and **16**. Overall from the SAR study, we can infer that dihydropyrazole-based compounds showed improvement in antitubercular activity over chalcones, and that compounds bearing halogen atoms such as fluorine and chlorine especially are crucial for activity in both chalcones and dihydropyrazole derivatives.

#### 2.2.3. Antiproliferative and Cytotoxicity Activity

In the monosubstituted chalcone series **2**–**6** and **9** activity ranged from 141 ± 2 to 432 ± 2 µM. In the disubstituted compounds **7**–**8**, the IC_50_ observed ranged from 31 ± 2 µM to 254 ± 2 µM, **7** being better than **8** by a factor of eight. In bioisosteric substitution, the activity ranged from 17 ± 1 to 353 ± 2 µM. The best activity in the chalcone series was observed for 2-thienyl bioisostere (**16)** with an IC_50_ of 17±1 µM, but this was found to be less than the reference standard methotrexate (11 ± 1 µM).

**Figure 4:**
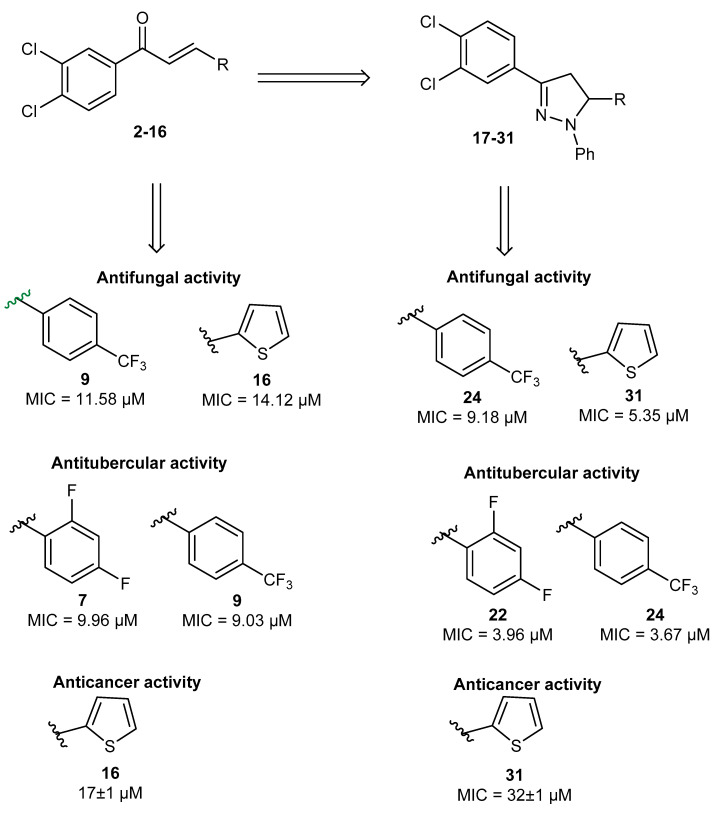
Summary of the antifungal, antitubercular, and antiproliferative activities of chalcones (**2**–**16**) and dihydropyrazoles (**17**–**31**).

Among the dihydropyrazoles **17**–**31**, activity ranged from 32 ± 1 to 351 ± 2 µM. Among the monosubstituted series **17**–**21** and **24**, the activity ranged from 134 ± 2 to 351 ± 2 µM. Substituting additional F or Cl (compounds **22** and **23**) did not bring about any improvement in activity over the monosubstituted compounds **19** and **21**. Among the bioisosteres **25**–**31**, activity ranged from 32 ± 2 to 271 ± 2 µM. The best activity was observed for 2-thienyl bioisostere (**31**) with ab IC_50_ of 32 ± 1 µM. This activity was found to be three times lower than the activity of the reference standard methotrexate (11 ± 1 µM). Overall, the chalcones fared better than the dihydropyrazoles in antiproliferative activity (Table 3). The cytotoxicity activity of chalcones **2**–**16** was more than 50 µg/mL whereas that of dihydropyrazoles and methotrexate (positive control) was greater than 75 µg/mL on the normal cell lines (L02) (Table 4). Hence, these compounds were non-toxic against normal human cells. A summary of the antifungal, antitubercular, and antiproliferative activities of chalcones (**2**–**16**) and dihydropyrazoles (**17**–**31**) is depicted in Figure 4.

## 3. Materials and Methods

### 3.1. Chemicals and Instruments

The chemicals, solvents, and reagents were purchased from S D Fine-Chem and Sigma Aldrich and were used without further purification. Thin layer chromatography (TLC) was carried out on pre-coated silica gel 60 F_254_ plates to monitor the reactions as well as to check the purity of the final products. The spots were visualized by UV lamp (254 nm). The chalcones were purified by recrystallization, whereas the dihydropyrazoles were purified by column chromatography employing silica gel (200 to 300 mesh) as the stationary phase and hexane-ethyl acetate as the mobile phase. Melting points were determined using Boetius melting point apparatus in open capillary tubes and were uncorrected. FTIR spectra were scanned using KBr discs on a Bruker Vertex 80v spectrometer whereas the ^1^H and ^13^C-NMR spectra were recorded on a Bruker AMX 400 MHz NMR spectrophotometer by dissolving the compounds in CDCl_3_ with TMS as an internal standard. The ^1^H-NMR and ^13^C-NMR spectra were recorded at operating frequencies of 400 and 100 MHz respectively and the chemical shift values are given in parts per million (ppm) relative to TMS. Mass spectra (MS) were obtained on Agilent 6100 QQQ ESI mass spectrophotometer by the electron spray ionization technique.

### 3.2. Synthesis

#### 3.2.1. General Procedure for the Synthesis of Chalcones (**17**–**31**)

A mixture of 3,4-dichloroacetophenone (**1**) (1 mmol) and the appropriate aryl or heteroaryl aldehyde (1 mmol) was stirred in ethanol (7.5 mL) and an alcoholic solution of KOH (50%, 7.5 mL) was added dropwise to it. The mixture was stirred for a period of 24 h, and it was then transferred into crushed ice. Later, the precipitate formed was filtered under vacuum and the product was washed thoroughly with water to remove the water-soluble impurities and dried. The dried precipitate was further recrystallized from ethanol to obtain the crystals of chalcone derivatives (**2**–**16**).

*(E)-1-(3,4-dichlorophenyl)-3-(2,4-difluorophenyl)prop-2-en-1-one* (**7**): Yield 83%; m.p. 265 °C; FTIR (KBr, cm^−1^): 1656 (C=O), 1581 (C=C of Ar), 1512 (CH=CH), 829 (C-Cl), 926 (C-F); ^1^H-NMR (400 MHz, CDCl_3_, ppm): 7.28 (1H, d, *J* = 17 Hz, -CO-CH=), 7.75 (1H, d, *J* = 17 Hz, =CH-Ar), 7.33–8.05 (6H, Ar-H).

*(E)-1-(3,4-dichlorophenyl)-3-(2,4-dichlorophenyl)prop-2-en-1-one* (**8**): Yield 91%; m.p. 299 °C; FTIR (KBr, cm^−1^): 1659 (C=O), 1582 (C=C of Ar), 1509 (CH=CH), 826 (C-Cl), 822 (C-Cl); ^1^H-NMR (400 MHz, CDCl_3_, ppm): 7.31 (1H, d, *J* = 17 Hz, -CO-CH=), 7.80 (1H, d, *J* = 17 Hz, =CH-Ar), 7.45–8.25 (6H, Ar-H).

*(E)-1-(3,4-dichlorophenyl)-3-(4-trifluoromethylphenyl)prop-2-en-1-one* (**9**): Yield 75%; m.p. 169 °C; FTIR (KBr, cm^−1^): 1652 (C=O), 1586 (C=C quadrant of Ar), 1521 (CH=CH), 922 (C-F); ^1^H-NMR (400 MHz, CDCl_3_, ppm): 7.35 (1H, d, *J* = 17 Hz, -CO-CH=), 7.69 (1H, d, *J* = 17 Hz, =CH-Ar), 7.22–8.09 (7H, Ar-H).

*(E)-1-(3,4-dichlorophenyl)-3-(pyridin-2-yl)prop-2-en-1-one* (**11**): Yield 43%; m.p. 156 °C; FTIR (KBr, cm^−1^): 1656 (C=O), 1600 (C=C of Ar), 1598 (C=N), 1512 (CH=CH), 1377 (C-N), 822 (C-Cl); ^1^H-NMR (400 MHz, CDCl_3_, ppm): 7.18 (1H, d, *J* = 17 Hz, -CO-CH=), 7.61 (1H, d, *J* = 17 Hz, =CH-Ar), 6.37–8.15 (7H, Ar-H).

*(E)-1-(3,4-dichlorophenyl)-3-(pyridin-3-yl)prop-2-en-1-one* (**12**): Yield 53%; m.p. 191 °C; FTIR (KBr, cm^−1^): 1655 (C=O), 1602 (C=C of Ar), 1591 (C=N), 1510 (CH=CH), 1371 (C-N), 828 (C-Cl); ^1^H-NMR (400 MHz, CDCl_3_, ppm): 7.19 (1H, d, *J* = 17 Hz, -CO-CH=), 7.71 (1H, d, *J* = 17 Hz, =CH-Ar), 7.11–8.25 (7H, Ar-H).

*(E)-1-(3,4-dichlorophenyl)-3-(furan-2-yl)prop-2-en-1-one* (**14**): Yield 55%; m.p. 172 °C; FTIR (KBr, cm^−1^): 1649 (C=O), 1581 (C=C of Ar), 1515 (CH=CH), 836 (C-Cl); ^1^H-NMR (400 MHz, CDCl_3_, ppm): 7.20 (1H, d, *J* = 17 Hz, -CO-CH=), 7.72 (1H, d, *J* = 17 Hz, =CH-Ar), 7.19–7.91 (6H, Ar-H).

*(E)-1-(3,4-dichlorophenyl)-3-(1H-pyrrol-2-yl)prop-2-en-1-one* (**15**): Yield 46%; m.p. 238 °C; FTIR (KBr, cm^−1^): 1656 (C=O), 1601 (C=C of Ar), 1581 (C=N), 1512 (CH=CH), 1375 (C-N), 3233 (N-H), 821 (C-Cl); ^1^H-NMR (400 MHz, CDCl_3_, ppm): 5.12 (1H, s, -NH), 7.08 (1H, d, *J* = 17 Hz, -CO-CH=), 7.68 (1H, d, *J* = 17 Hz, =CH-Ar), 6.45–7.95 (6H, Ar-H).

*(E)-1-(3,4-dichlorophenyl)-3-(thiophen-2-yl)prop-2-en-1-one* (**16**): Yield 58%; m.p. 194 °C; FTIR (KBr, cm^−1^): 1661 (C=O), 1611 (C=C of Ar), 1519 (CH=CH), 628 (C-S), 823 (C-Cl); ^1^H-NMR (400 MHz, CDCl_3_, ppm): 7.14 (1H, d, *J* = 17 Hz, -CO-CH=), 7.72 (1H, d, *J* = 17 Hz, =CH-Ar), 6.69–8.14 (6H, Ar-H).

#### 3.2.2. General Procedure for the Synthesis of Dihydropyrazoles (**17**–**31**)

Chalcones of 3,4-dichloroacetophenone (0.005 mol) and phenyl hydrazine hydrochloride (0.005 mol) were dissolved in glacial acetic acid (10 mL). Later, the mixture was refluxed for 6–8 h, the solvent was evaporated completely, and dried solid was obtained. The solid thus obtained was purified by silica gel column chromatography using mixtures of ethyl acetate in n-hexane (5–75%) and further recrystallized from chloroform.

*3-(3,4-dichlorophenyl)-1-phenyl-5-(p-tolyl)-4,5-dihydro-1H-pyrazole* (**17**): Yield 55%; m.p. 168 °C; FTIR (KBr, cm^−1^): 1592 (C=N), 1120 (C-N), 1586 (C=C quadrant of Ar), 830 (C-Cl); ^1^H-NMR (400 MHz, CDCl_3_, ppm): 3.28 (1H, d, HA), 3.72 (1H, d, HB), 5.45 (1H, d, Hx), 2.34 (3H, s, Ar-CH3), 6.75–7.91 (12H, Ar-H); ^13^C-NMR (100 MHz, CDCl_3_, ppm): 22.46 (-CH_3_), 66.54 (C-4), 45.50 (C-5), 152.50 (C-3), 133.95 (C-2′ and C-2″), 143.51 (C-3′ and C-3″), 134.88 (C-4′ and C-4″), 112.12–145.96 (Ar-C’s); MS (*m/z*, %): 381.30 (M^+^), 383.30 (M + 2).

*3-(3,4-dichlorophenyl)-1-phenyl-5-(4-fluorophenyl)-4,5-dihydro-1H-pyrazole* (**18**): Yield 70%; m.p. 195 °C; FTIR (KBr, cm^−1^): 1644 (C=N), 1099 (C-N), 1582 (C=C quadrant of Ar), 835 (C-Cl), 922 (C-F); ^1^H-NMR (400 MHz, CDCl_3_, ppm): 3.29 (1H, d, HA), 3.75 (1H, d, HB), 5.33 (1H, d, HX), 6.65–7.65 (12H, Ar-H).

*3-(3,4-dichlorophenyl)-1-phenyl-5-(2-fluorophenyl)-4,5-dihydro-1H-pyrazole* (**19**): Yield 61%; m.p. 149 °C; FTIR (KBr, cm^−1^): 1599 (C=N), 1105 (C-N), 1578 (C=C quadrant of Ar), 825 (C-Cl), 925 (C-F); ^1^H-NMR (400 MHz, CDCl_3_, ppm): 3.26 (1H, d, HA), 3.72 (1H, d, HB), 5.46 (1H, d, HX), 6.66–7.55 (12H, Ar-H).

*3-(3,4-dichlorophenyl)-1-phenyl-5-(4-chlorophenyl)-4,5-dihydro-1H-pyrazole* (**20**): Yield 75%; m.p. 261 °C; FTIR (KBr, cm^−1^): 1549 (C=N), 1091 (C-N), 1582 (C=C quadrant of Ar), 838 (C-Cl), 846 (C-Cl); ^1^H-NMR (400 MHz, CDCl_3_, ppm): 3.28 (1H, d, HA), 3.77 (1H, d, HB), 5.49 (1H, d, HX), 6.56–7.39 (12H, Ar-H).

*3-(3,4-dichlorophenyl)-1-phenyl-5-(2-chlorophenyl)-4,5-dihydro-1H-pyrazole* (**21**): Yield 72%; m.p. 133 °C; FTIR (KBr, cm^−1^): 1552 (C=O), 1591 (C=C quadrant of Ar), 832 (C-Cl), 838 (C-Cl); ^1^H-NMR (400 MHz, CDCl_3_, ppm): 3.24 (1H, d, HA), 3.71 (1H, d, HB), 5.42 (1H, d, HX), 6.69–7.42 (12H, Ar-H).

*3-(3,4-dichlorophenyl)-1-phenyl-5-(2,4-difluorophenyl)-4,5-dihydro-1H-pyrazole* (**22**): Yield 81%; m.p. 291 °C; FTIR (KBr, cm^−1^): 1526 (C=N), 1086 (C-N), 1588 (C=C quadrant of Ar), 831 (C-Cl), 921 (C-F); ^1^H-NMR (400 MHz, CDCl_3_, ppm): 3.25 (1H, d, HA), 3.73 (1H, d, HB), 5.49 (1H, d, HX), 6.87–7.69 (11H, Ar-H).

*3-(3,4-dichlorophenyl)-1-phenyl-5-(2,4-dichlorophenyl)-4,5-dihydro-1H-pyrazole* (**23**): Yield 85%; m.p. 302 °C; FTIR (KBr, cm^−1^): 1544 (C=N), 1081 (C-N), 1578 (C=C quadrant of Ar), 821 (C-Cl), 829 (C-Cl); ^1^H-NMR (400 MHz, CDCl_3_, ppm): 3.26 (1H, d, HA), 3.81 (1H, d, HB), 5.52 (1H, d, HX), 6.82–7.77 (11H, Ar-H).

*3-(3,4-dichlorophenyl)-1-phenyl-5-(4-trifluoromethylphenyl)-4,5-dihydro-1H-pyrazole* (**24**): Yield 66%; m.p. 125 °C; FTIR (KBr, cm^−1^): 1641 (C=N), 1152 (C-N), 1568 (C=C quadrant of Ar), 818 (C-Cl), 926 (C-F); ^1^H-NMR (400 MHz, CDCl_3_, ppm): 3.28 (1H, d, HA), 3.76 (1H, d, HB), 5.50 (1H, d, HX), 6.61–7.81 (12H, Ar-H).

*3-(3,4-dichlorophenyl)-1-phenyl-5-(benzo[d][1,3]dioxol-5-yl)-4,5-dihydro-1H-pyrazole* (**25**): Yield 49%; m.p. 212 °C; FTIR (KBr, cm^−1^): 1595 (C=N), 1097 (C-N), 1576 (C=C quadrant of Ar), 1236 (O-CH2-O), 845 (C-Cl); ^1^H-NMR (400 MHz, CDCl_3_, ppm): 6.25 (2H,s,-O-CH2O-), 3.18 (1H, d, HA), 3.82 (1H, d, HB), 5.28 (1H, d, HX), 6.74–7.49 (11H, Ar-H).

*3-(3,4-dichlorophenyl)-1-phenyl-5-(pyridin-2-yl)-4,5-dihydro-1H-pyrazole* (**26**): Yield 55%; m.p. 191 °C; FTIR (KBr, cm^−1^): 1645 (C=N), 1082 (C-N), 1591 (C=N), 1559 (C=C quadrant of Ar), 1325 (C-N); ^1^H-NMR (400 MHz, CDCl_3_, ppm): 3.17 (1H, d, HA), 3.84 (1H, d, HB), 5.29 (1H, d, HX), 6.39–7.89 (12H, Ar-H).

*3-(3,4-dichlorophenyl)-1-phenyl-5-(pyridin-3-yl)-4,5-dihydro-1H-pyrazole* (**27**): Yield 53%; m.p. 184 °C; FTIR (KBr, cm^−1^): 1634 (C=N) 1099 (C-N), 1591 (C=N), 1568 (C=C quadrant of Ar), 1344 (C-N); ^1^H-NMR (400 MHz, CDCl_3_, ppm): 3.04 (1H, d, HA), 3.90 (1H, d, HB), 5.59 (1H, d, HX), 6.69–7.59 (12H, Ar-H).

*3-(3,4-dichlorophenyl)-1-phenyl-5-(pyridin-4-yl)-4,5-dihydro-1H-pyrazole* (**28**): Yield 61%; m.p. 214 °C; FTIR (KBr, cm^−1^): 1641 (C=N), 1086 (C-N), 1577 (C=N), 1559 (C=C quadrant of Ar), 1335 (C-N); ^1^H-NMR (400 MHz, CDCl_3_, ppm): 3.17 (1H, d, HA), 3.87 (1H, d, HB), 5.27 (1H, d, HX), 6.79–7.44 (12H, Ar-H).

*3-(3,4-dichlorophenyl)-1-phenyl-5-(furan-2-yl)-4,5-dihydro-1H-pyrazole* (**29**): Yield 58%; m.p. 200 °C; FTIR (KBr, cm^−1^): 1612 (C=N), 1091 (C-N), 1555 (C=C quadrant of Ar), 835 (C-Cl); ^1^H-NMR (400 MHz, CDCl_3_, ppm): 3.14 (1H, d, HA), 3.85 (1H, d, HB), 5.34 (1H, d, HX), 6.44–7.79 (11H, Ar-H).

*3-(3,4-dichlorophenyl)-1-phenyl-5-(1H-pyrrol-2-yl)-4,5-dihydro-1H-pyrazole* (**30**): Yield 48%; m.p. 171 °C; FTIR (KBr, cm^−1^): 1643 (C=N), 1087 (C-N), 1564 (C=C quadrant of Ar), 1584 (C=N), 3240 (N-H), 1371 (C-N), 828 (C-Cl); ^1^H-NMR (400 MHz, CDCl_3_, ppm): 3.14 (1H, d, HA), 3.84 (1H, d, HB), 5.56 (1H, s, -NH), 5.24 (1H, d, HX), 6.49–7.59 (11H, Ar-H).

*3-(3,4-dichlorophenyl)-1-phenyl-5-(thiophen-2-yl)-4,5-dihydro-1H-pyrazole* (**31**): Yield 41%; m.p. 212 °C; FTIR (KBr, cm^−1^): 1646 (C=N), 1145 (C-N), 1549 (C=C quadrant of Ar), 633 (C-S), 826 (C-Cl); ^1^H-NMR (400 MHz, CDCl_3_, ppm): 3.49 (1H, d, HA), 3.77 (1H, d, HB), 5.19 (1H, d, HX), 6.44–7.59 (11H, Ar-H).

### 3.3. Biological Activity Studies

#### 3.3.1. Antifungal Activity

The antifungal activity of the novel chalcones (**2** to **16**) and dihydropyrazoles (**17** to **31**) was measured against selected fungal strains such as *Aspergillus niger* (ATCC-6275, *An*) and *Candida tropicalis* (ATCC-1369, *Ct*) using a standard protocol published in the literature [26,27]. The serial tube dilution method employed during the anti-fungal assay determined the minimum inhibitory concentration (MIC) values of compounds. Fluconazole was used as a standard (positive control) drug to compare the activity. Initially, 2.048 mg of a test compound or fluconazole was dissolved in 2 mL of methanol to obtain a concentration of 1.024 mg/mL as stock solution. The test fungal organism was grown at 37 °C in Potato Dextrose Agar followed by dilution with sterile nutrient broth medium to obtain a fungal suspension containing about 10^7^ cells/mL. This suspension was used as the inoculum. This antifungal assay utilized 11 test tubes, 9 of which were marked as 1, 2, 3, 4, 5, 6, 7, 8, and 9 whereas the remaining two were assigned the names T_M_ (medium), and T_MI_ (medium + inoculum). An amount of 1 mL of nutrient broth medium was transferred in to each of these 11 test tubes and then cotton plugged followed by sterilization in an autoclave at a temperature of 121 °C for 30 min under the pressure of 15 psi.

Once these sterile test tubes were cooled down, 1 mL of the stock solution of a test compound was added to the first test tube and mixed well followed by transferring 1 mL of this content to the second test tube. The process of serial dilution was continued up to the ninth test tube. Then, 10 μL of diluted inoculum containing about 10^7^ cells/mL of a fungal strain was added to each of the nine test tubes and mixed well. Another 10 μL of the inoculum was also added to the test tube marked T_MI_ to observe the growth of the organism in the medium used. The control test tube T_M_ containing only the medium was used to confirm the sterility of the medium. All the test tubes were incubated at 37 °C for 18 h. A similar experiment with medium, methanol, and inoculum without the compound was also performed to ensure that the methanol (negative control) had no inhibitory effect in the dilutions used. The test tube number in which the first sign of growth of the organism was observed was noted. The MIC was calculated as the concentration used in the test tube number just prior to the test tube number where the first sign of growth was observed. This procedure was followed in triplicate to determine the MIC values for all the compounds and the results were considered the average of three values. As the standard drug fluconazole is structurally different from the target compounds, the MIC values obtained in µg/mL were converted to molar concentration in the form of µM.

#### 3.3.2. Antitubercular Activity

The antitubercular activity of all the test compounds was tested for against the *Mycobacterium tuberculosis H37Rv* strain, employing pyrazinamide as the reference standard according to the standard procedure described in the literature [21,22,24,25,27,48]. A frozen culture in Middlebrook 7H9 broth with the addition of 0.2% glycerol and 10% albumin-dextrose-catalase was thawed and diluted in broth to 10^5^ CFU mL^−1^ (colony forming unit/mL) dilutions. Each test compound was dissolved separately in DMSO followed by dilution with broth to attain a concentration which was two times the required concentration. During this experiment, the final concentration of DMSO in the assay medium was 1.3%. Each U-tube was then inoculated with 0.05 mL of standardized culture and later incubated at 37 °C for 21 days. The growth in the U-tubes was compared with visibility in opposition to a positive control (with pyrazinamide) and negative control (without drug and inoculum). A broth dilution assay was utilized to determine the minimum inhibitory concentration (MIC) of each compound. MIC is defined as the lowest concentration of drug or a compound that inhibits ≤ 99% of the bacteria present at the start of the assay. The MIC values obtained in µg/mL were converted to molar concentration in the form of µM as the standard drug pyrazinamide and the target compounds (**2**–**31**) were structurally diverse.

#### 3.3.3. Antiproliferative and Cytotoxic Activity

The in vitro antiproliferative activity of chalcones (**2** to **16**) and dihydropyrazoles (**17** to **31**) was evaluated by Mosmann’s MTT assay, as described previously [28] on prostate cancer cell lines (DU-145). The antiproliferative activity of the compounds was determined their IC_50_ values. Methotrexate (Mtx; positive control) was used as a reference standard for comparing the antiproliferative activity of the target compounds. The MTT (3-(4,5-dimethylthiazol-2-yl)-2,5-diphenyl tetrazolium bromide) assay was based on the reduction of the soluble MTT (0.5 mg mL^−1^, 100 µL) into a blue-purple formazan product, mainly by the activity of mitochondrial reductase enzymes inside the living cells. The prostate cancer cell lines, DU-145 cells, were cultured in Dulbecco’s Modified Eagle Medium (DMEM) media at 37 °C and humidified at 5% CO_2_. Stock solutions of the test compounds (**17**–**46**) were prepared in 0.1% DMSO followed by dilution with sterile water to obtain the desired final concentrations. Briefly, the cells were placed on 96-well plates at 100 µL total volume with the density of 1 × 10^4^ cells per well and were allowed to adhere for 24 h. Then the medium was replaced with fresh media containing different dilutions of the test compounds and incubated for additional 48 h at 37 °C in DMEM with 10% fetal bovine serum (FBS) medium. Subsequently, the medium was replaced with 90 μL of fresh DMEM without FBS. The above wells were treated with 10 μL of MTT reagent (5 mg/mL of stock solution in DMEM without FBS) and incubated at 37 °C for 3–4 h. The formed blue formazan crystals were dissolved in 200 μL of DMSO. The optical density was determined at 570 nm using a micro plate reader. The assay was performed in triplicate for three independent experiments. The same experimentation was also done to confirm that the DMSO (negative control) had no effect in the study. The results had good reproducibility between replicate wells with standard errors below 10%. The antiproliferative activity results measured as IC_50_ values in µg/mL were converted and expressed in µM. Additionally, the cytotoxic activity of the compounds was also measured employing the same protocol discussed above to assess their toxicity on normal human cell lines (L02).

## 4. Conclusions

In the present study, we reported the antifungal, antitubercular, and antiproliferative activity and structure–activity relationship of 30 dichlorophenyl ring-containing compounds including 15 chalcones and 15 dihydropyrazoles. Biological screening data indicated that chalcones exhibited better antiproliferative activity over dihydropyrazoles whereas the dihydropyrazole derivatives showed superior antifungal and antitubercular activities. It was observed that the electronic property (electron withdrawing) of the substituents on the phenyl ring was instrumental in the potency of the compounds. The phenyl ring was substituted with various bioisosteres. For instance, the dihydropyrazole with a bioisostere 2-thienyl heterocycle (**31**) showed potent antifungal activity on chalcone **16**. Dihydropyrazoles **22** and **24** bearing 2,4-dichlorophenyl were the potent antitubercular compounds on chalcones **7** and **9** and the reference standard. Chalcone **16** showed better antiproliferative activity on dihydropyrazole **31** but less than the standard. Further studies are in progress to separate the enantiomers of the dihydropyrazoles as well as to assess the mode of action of the potential compounds that emerged from our study by biological and computational means.

## Figures and Tables

**Table 1:** Antifungal activities (MIC in µM) of chalcones (**2**–**16**) and dihydropyrazoles (**17**–**31**).

Compound	*A. niger*	*C. tropicalis*	Compound	*A. niger*	*C. tropicalis*
**2**	109.9	109.9	**17**	41.96	41.96
**3**	54.21	54.21	**18**	20.76	41.53
**4**	27.10	54.21	**19**	20.76	41.53
**5**	25.67	51.34	**20**	19.91	39.82
**6**	25.67	51.34	**21**	19.91	39.82
**7**	25.54	51.09	**22**	19.83	19.83
**8**	23.11	46.23	**23**	18.34	36.68
**9**		11.58	**24**	9.18	9.18
**10**	23.11	180.62	**25**	19.45	19.45
**11**	28.87	28.87	**26**	21.72	21.72
**12**	28.87	14.43	**27**	21.72	21.72
**13**	57.75	112.8	**28**	21.72	21.72
**14**	234.97	117.48	**29**	11.19	11.19
**15**	60.37	60.37	**30**	44.91	44.91
**16**	14.12	14.12	**31**	5.35	5.35
Fluconazole	26.11	19.58	Fluconazole	26.11	19.58

**Table 2:** Antitubercular activity (MIC in µM) of chalcones (**2**–**16**) and dihydropyrazoles (**17**–**31**).

Compound	*M. tuberculosis H_37_Rv*	Compound	*M. tuberculosis H_37_Rv*
**2**	343.44	**17**	262.26
**3**	84.70	**18**	32.44
**4**	84.70	**19**	32.44
**5**	80.23	**20**	31.11
**6**	20.05	**21**	15.55
**7**	9.96	**22**	3.96
**8**	18.06	**23**	7.15
**9**	9.03	**24**	3.67
**10**	72.24	**25**	60.78
**11**	45.12	**26**	67.88
**12**	22.56	**27**	67.88
**13**	45.12	**28**	67.88
**14**	46.99	**29**	17.49
**15**	47.16	**30**	70.17
**16**	22.07	**31**	8.35
Pyrazinamide	25.34		25.34

**Table 3:** Antiproliferative activity (IC_50_ in µM) of chalcones (**2**–**16**) and dihydropyrazoles (**17**–**31**).

Compound	DU-145	Compound	DU-145
**2**	432 ± 2	**17**	351 ± 2
**3**	176 ± 2	**18**	119 ± 2
**4**	393 ± 2	**19**	327 ± 2
**5**	147 ± 2	**20**	149 ± 2
**6**	141 ± 2	**21**	134 ± 2
**7**	31 ± 2	**22**	262 ± 2
**8**	254 ± 2	**23**	206 ± 2
**9**	292 ± 1	**24**	257 ± 2
**10**	138 ± 2	**25**	131 ± 2
**11**	353 ± 2	**26**	271 ± 2
**12**	256 ± 2	**27**	211 ± 2
**13**	209 ± 2	**28**	173 ± 2
**14**	206 ± 2	**29**	162 ± 2
**15**	94 ± 2	**30**	84 ± 2
**16**	17 ± 1	**31**	32 ± 1
Methotrexate	11 ± 1		

Data are presented as mean ± SD (*n* = 3). All the compounds and the standard were dissolved in dimethyl sulfoxide (DMSO), diluted with culture medium containing 0.1% DMSO. The control cells were treated with culture medium containing 0.1% DMSO.

**Table 4:** Cytotoxic activity (IC_50_ in µg/mL) of chalcones (**2–16**) and dihydropyrazoles (**17**–**31**).

Compound	L02	Compound	L02
**2**	>50	**17**	>75
**3**	>50	**18**	>75
**4**	>50	**19**	>75
**5**	>50	**20**	>75
**6**	>50	**21**	>75
**7**	>50	**22**	>75
**8**	>50	**23**	>75
**9**	>50	**24**	>75
**10**	>50	**25**	>75
**11**	>50	**26**	>75
**12**	>50	**27**	>75
**13**	>50	**28**	>75
**14**	>50	**29**	>75
**15**	>50	**30**	>75
**16**	>50	**31**	>75
Methotrexate	>75		

## Supplementary Materials

The supplementary materials are available online, FT-IR, ^1^H-NMR, ^13^C-NMR, and MS Spectral Data and Rf values.
