# Supplementary material for: Design, Facile Synthesis and Characterization of Dichloro Substituted Chalcones and Dihydropyrazole Derivatives for Their Antifungal, Antitubercular and Antiproliferative Activities
Source: Molecules. 2020 Jul 13;25(14):3188. doi: 10.3390/molecules25143188 (PMC7397056; doi:10.3390/molecules25143188)
Supplement: Supplementary file 1 [file molecules-25-03188-s001.pdf]

Design, Facile Synthesis and Characterization of Dichloro-Substituted Chalcones and Dihydropyrazole Derivatives for Their Antifungal, Antitubercular and Antiproliferative Activities

Supporting Information

FT-IR, ^{1}H-NMR, ^{13}C-NMR, and MS Spectral Data and *Rf* values

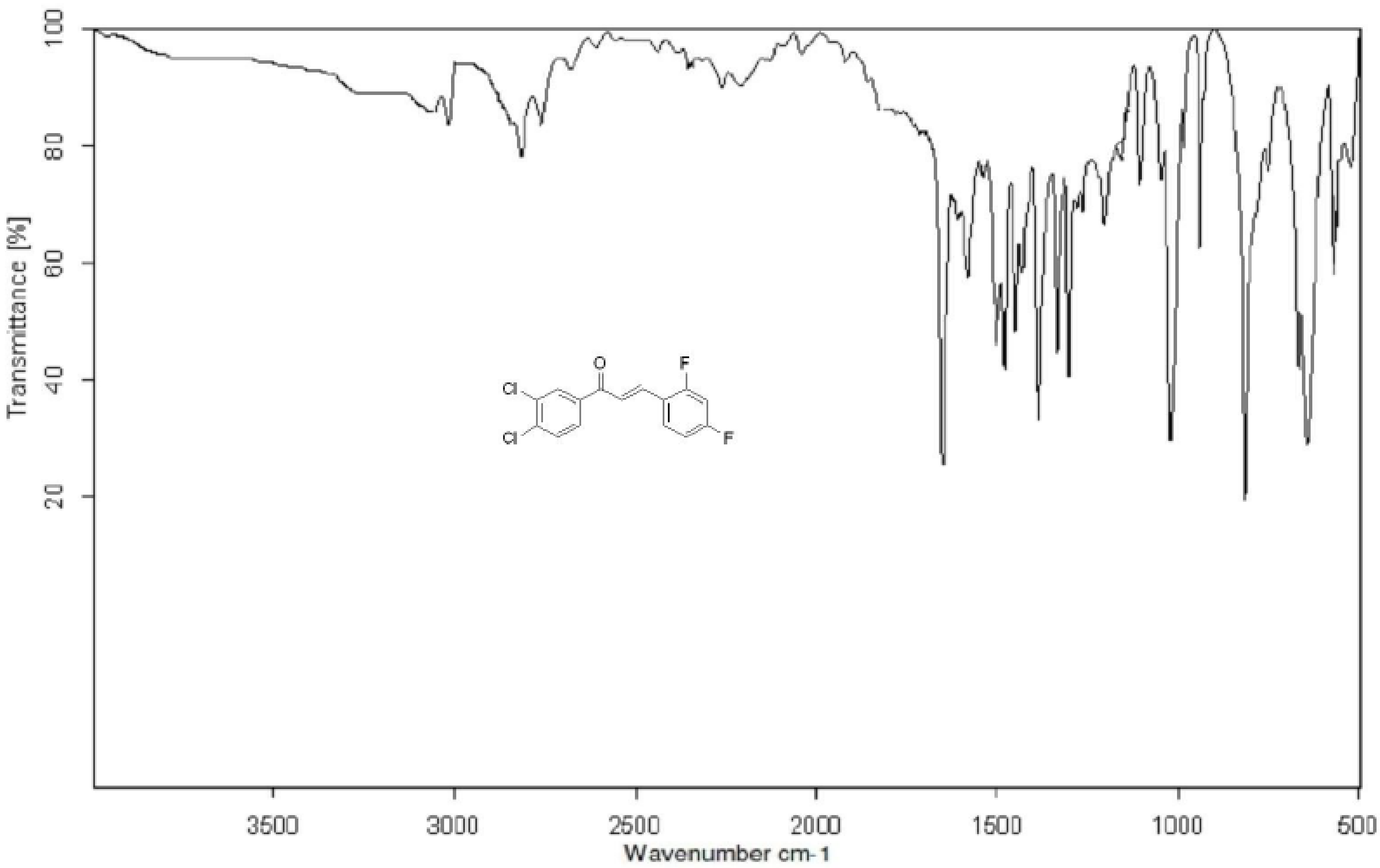

IR spectrum of (*E*)-1-(3,4-dichlorophenyl)-3-(2,4-difluorophenyl)prop-2-en-1-one (**7**).

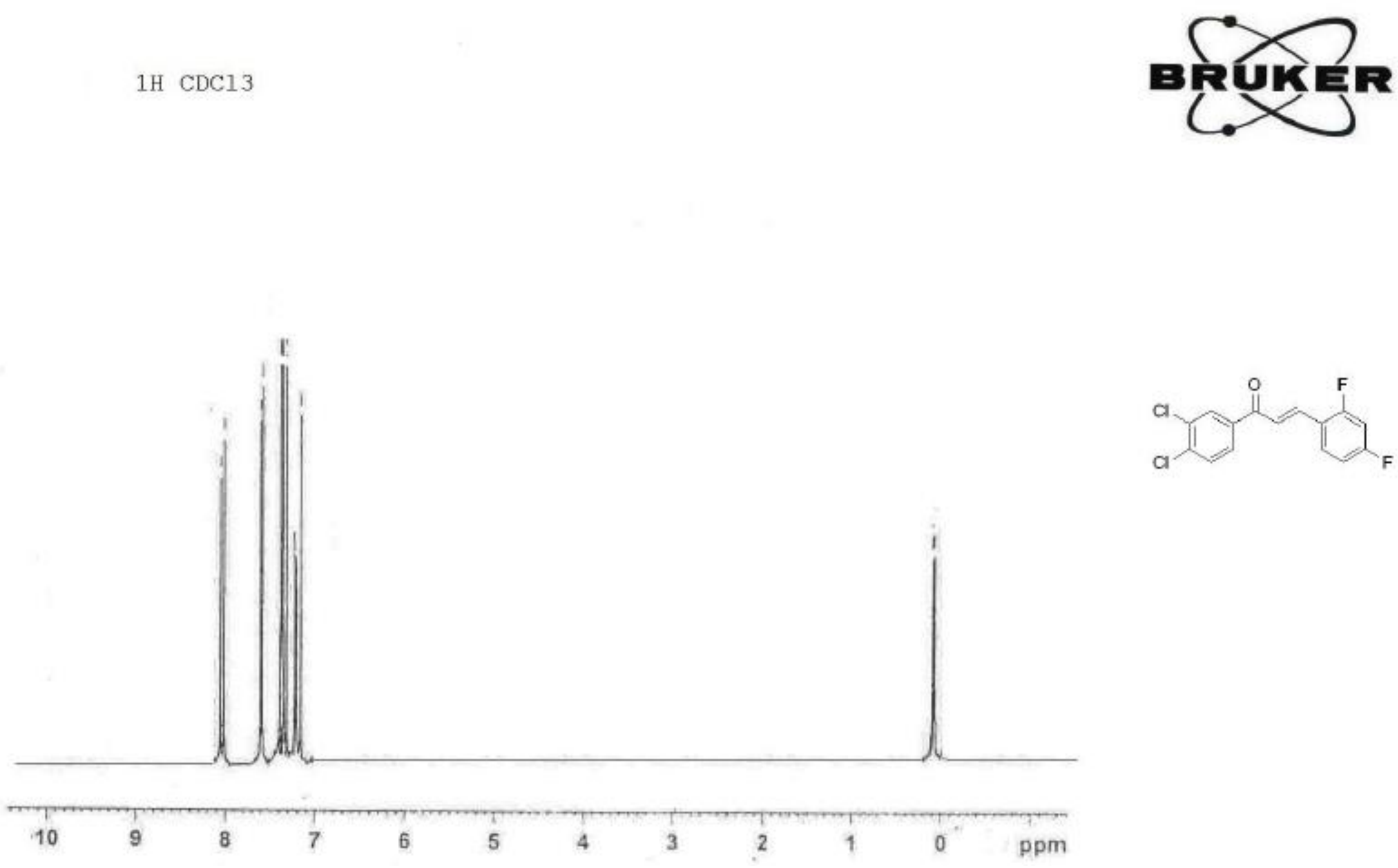

^{1}H-NMR spectrum of (*E*)-1-(3,4-dichlorophenyl)-3-(2,4-difluorophenyl)prop-2-en-1-one (**7**).

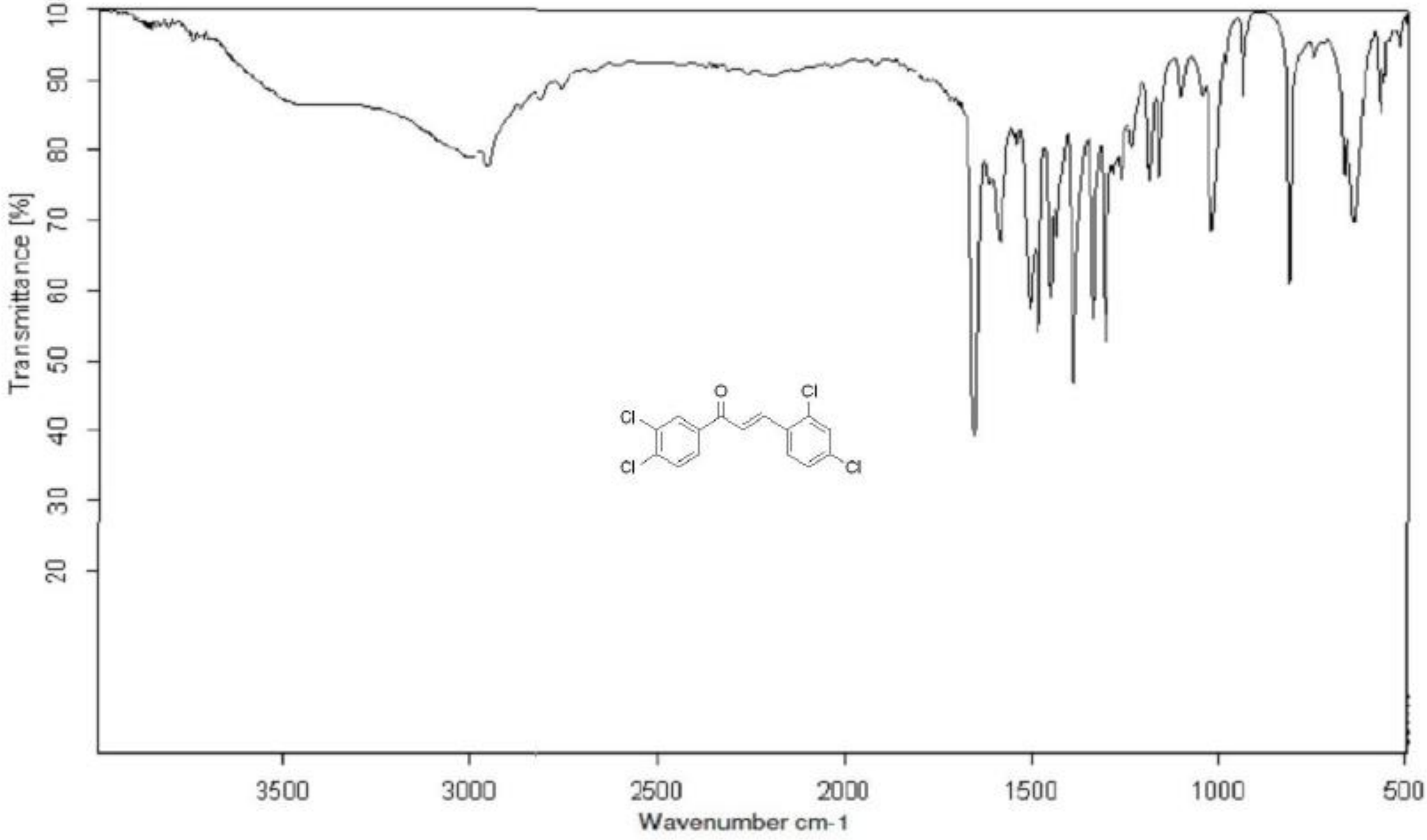

IR spectrum of (*E*)-1-(3,4-dichlorophenyl)-3-(2,4-dichlorophenyl)prop-2-en-1-one (**8**).

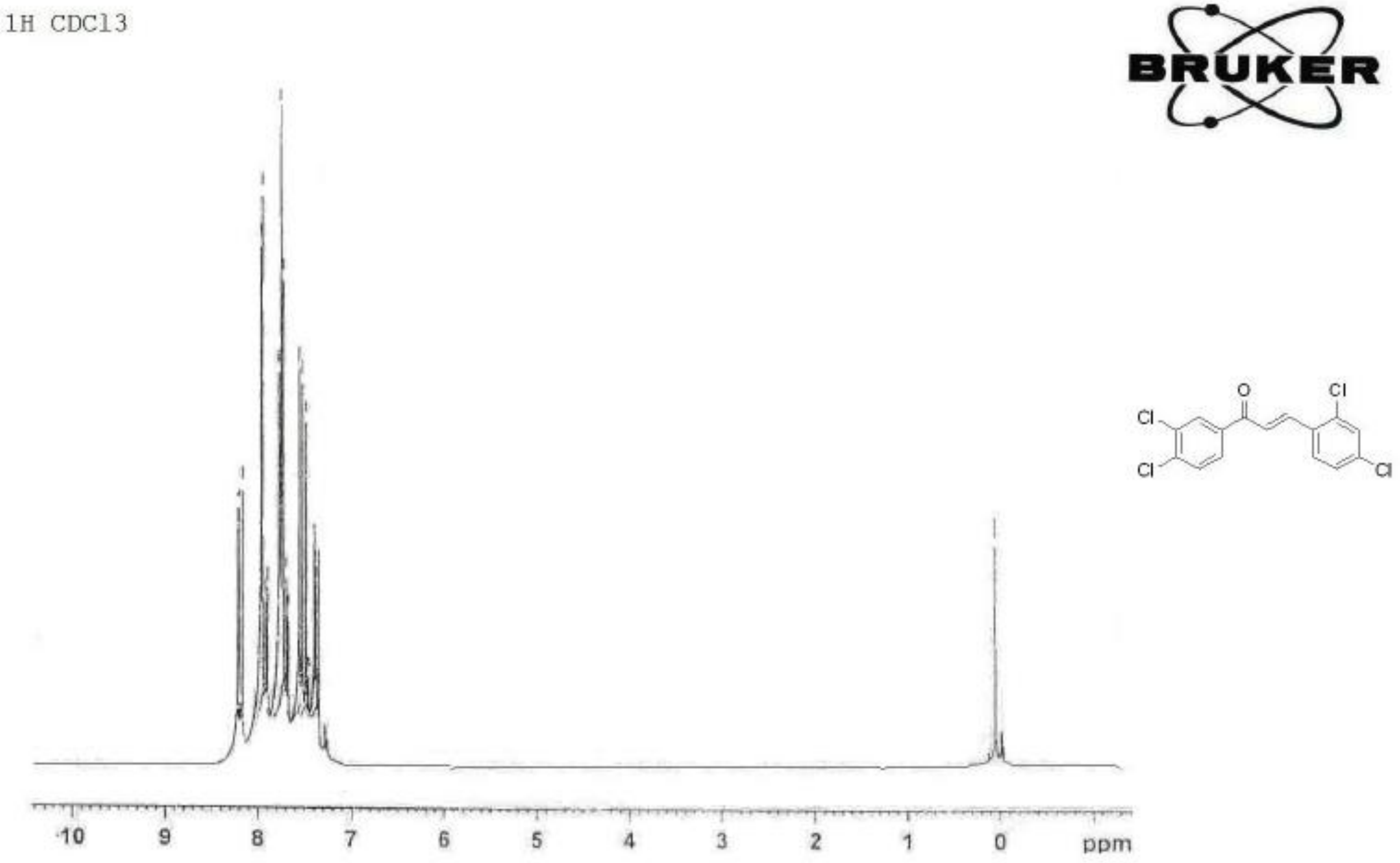

^{1}H-NMR spectrum of (*E*)-1-(3,4-dichlorophenyl)-3-(2,4-dichlorophenyl)prop-2-en-1-one (**8**).

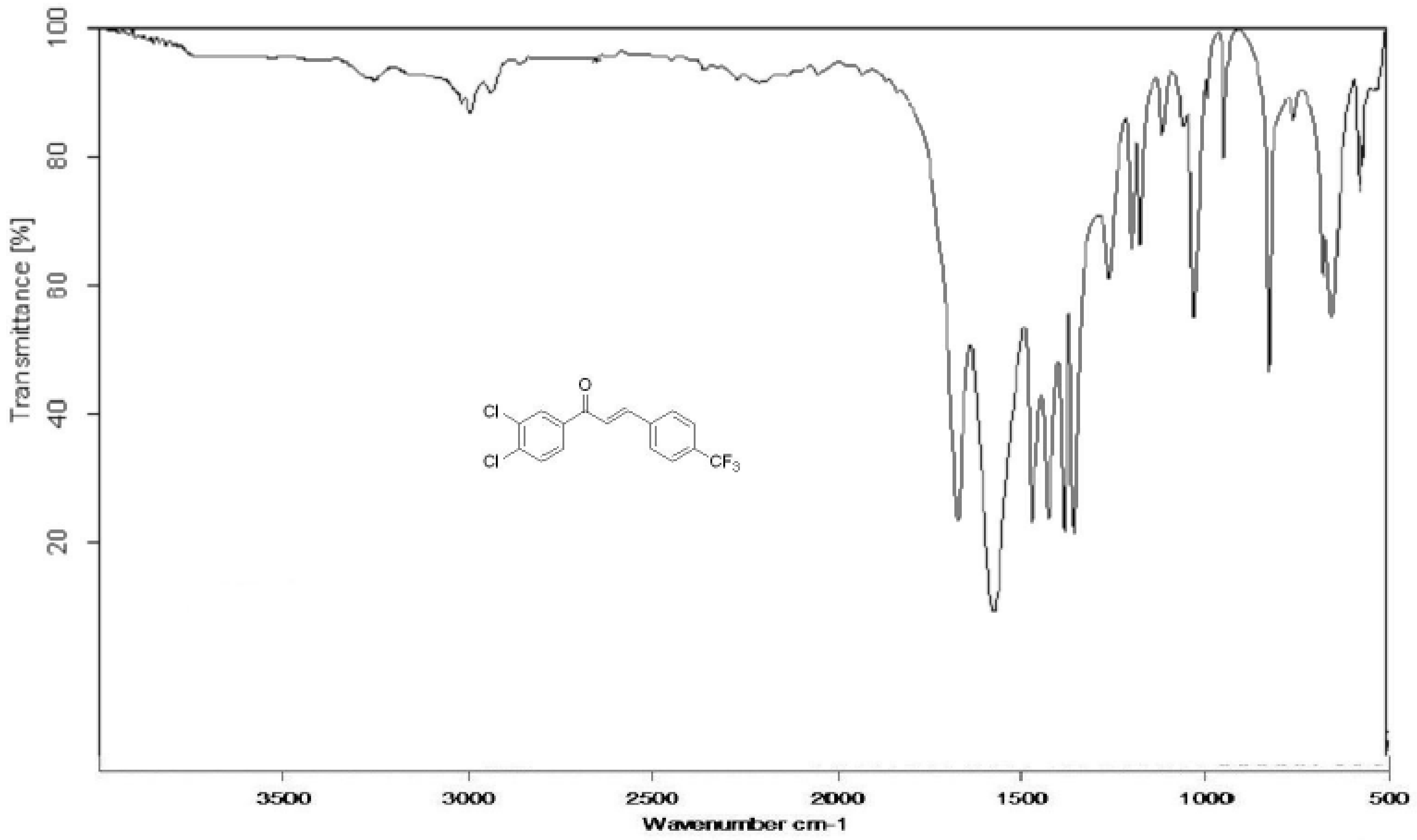

IR spectrum of (*E*)-1-(3,4-dichlorophenyl)-3-(4-trifluoromethylphenyl)prop-2-en-1-one (**9**).

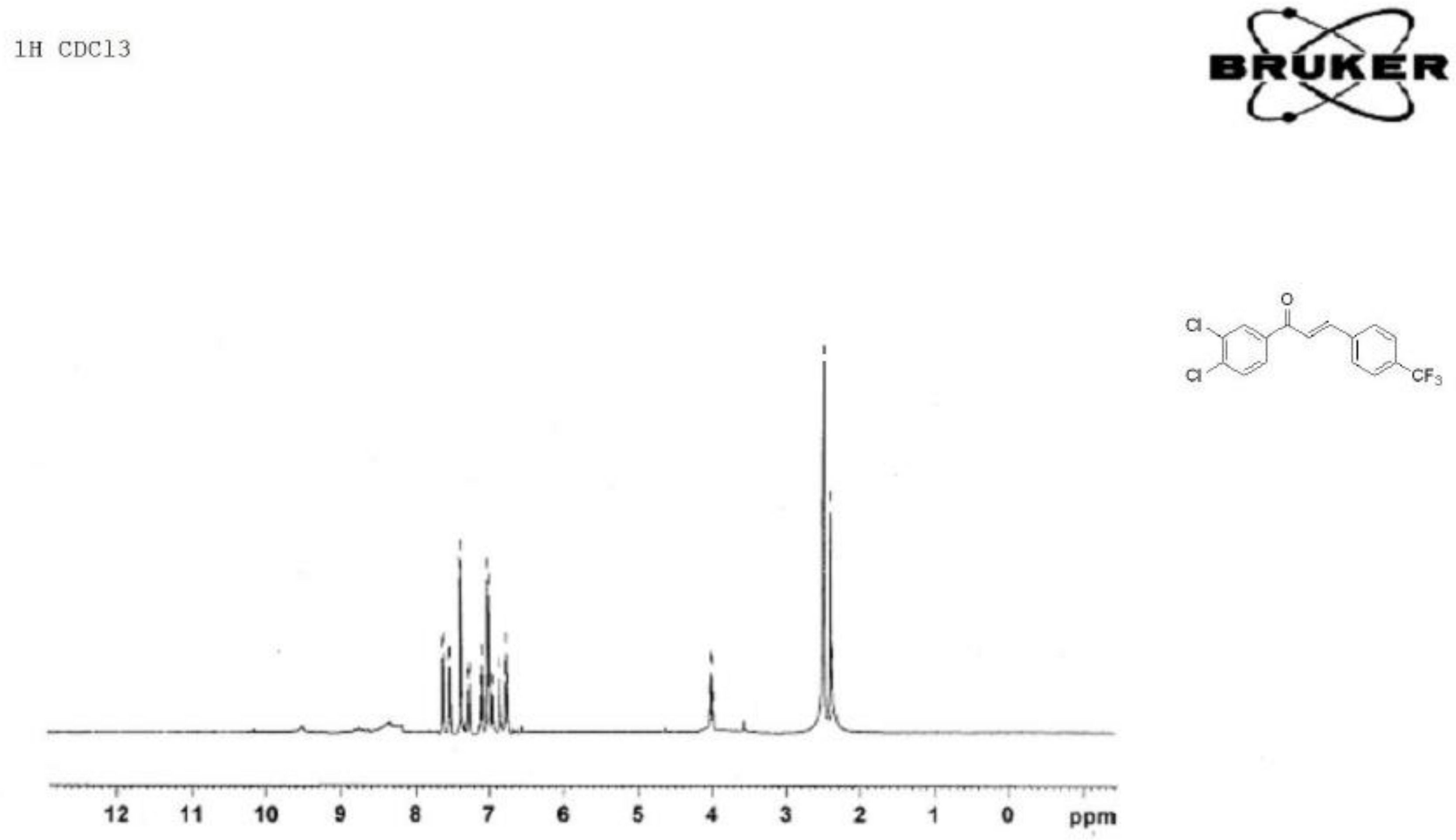

^{1}H-NMR spectrum of (*E*)-1-(3,4-dichlorophenyl)-3-(4-trifluoromethylphenyl)prop-2-en-1-one (**9**).

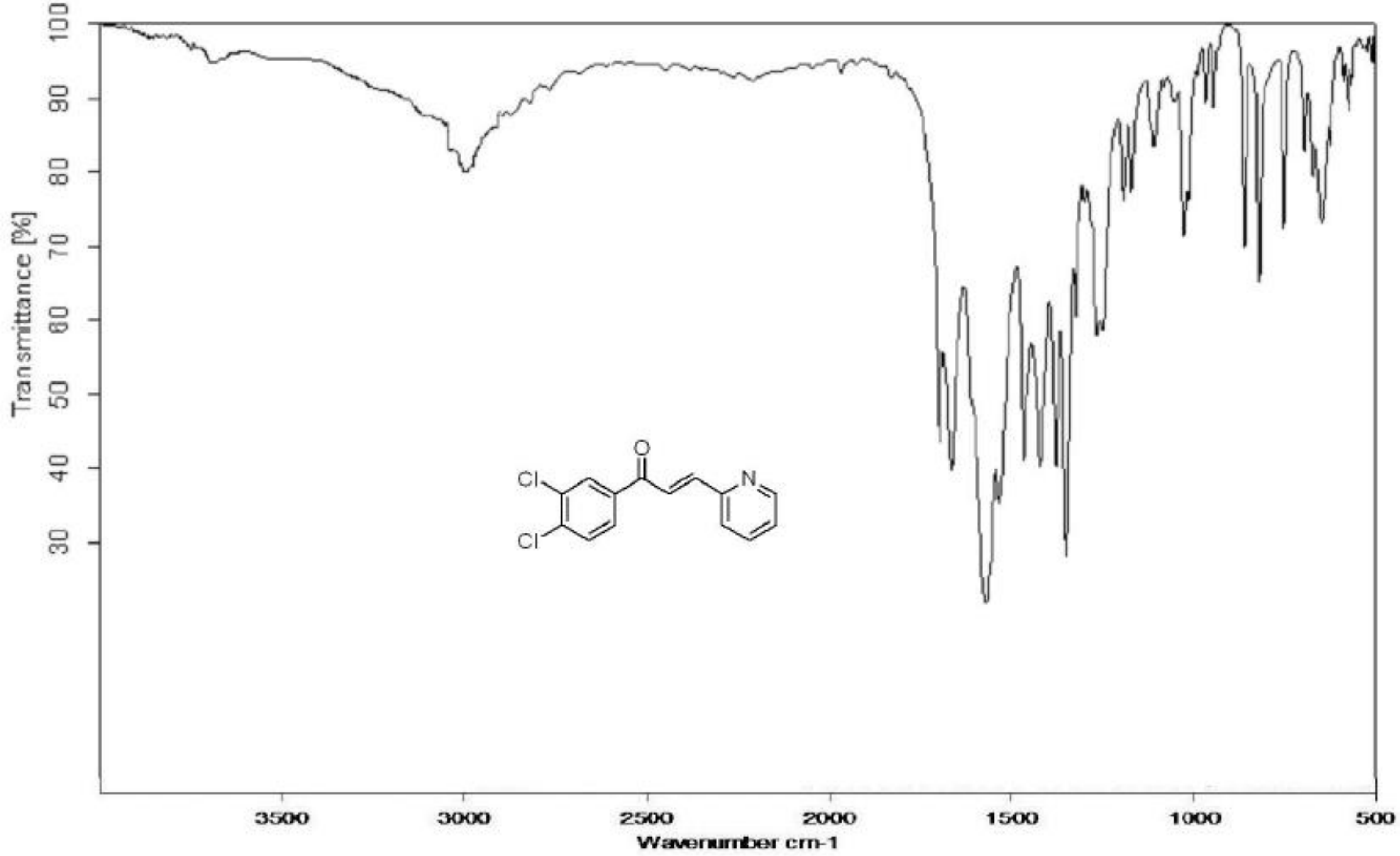

IR spectrum of (*E*)-1-(3,4-dichlorophenyl)-3-(pyridin-2-yl)prop-2-en-1-one (**11**).

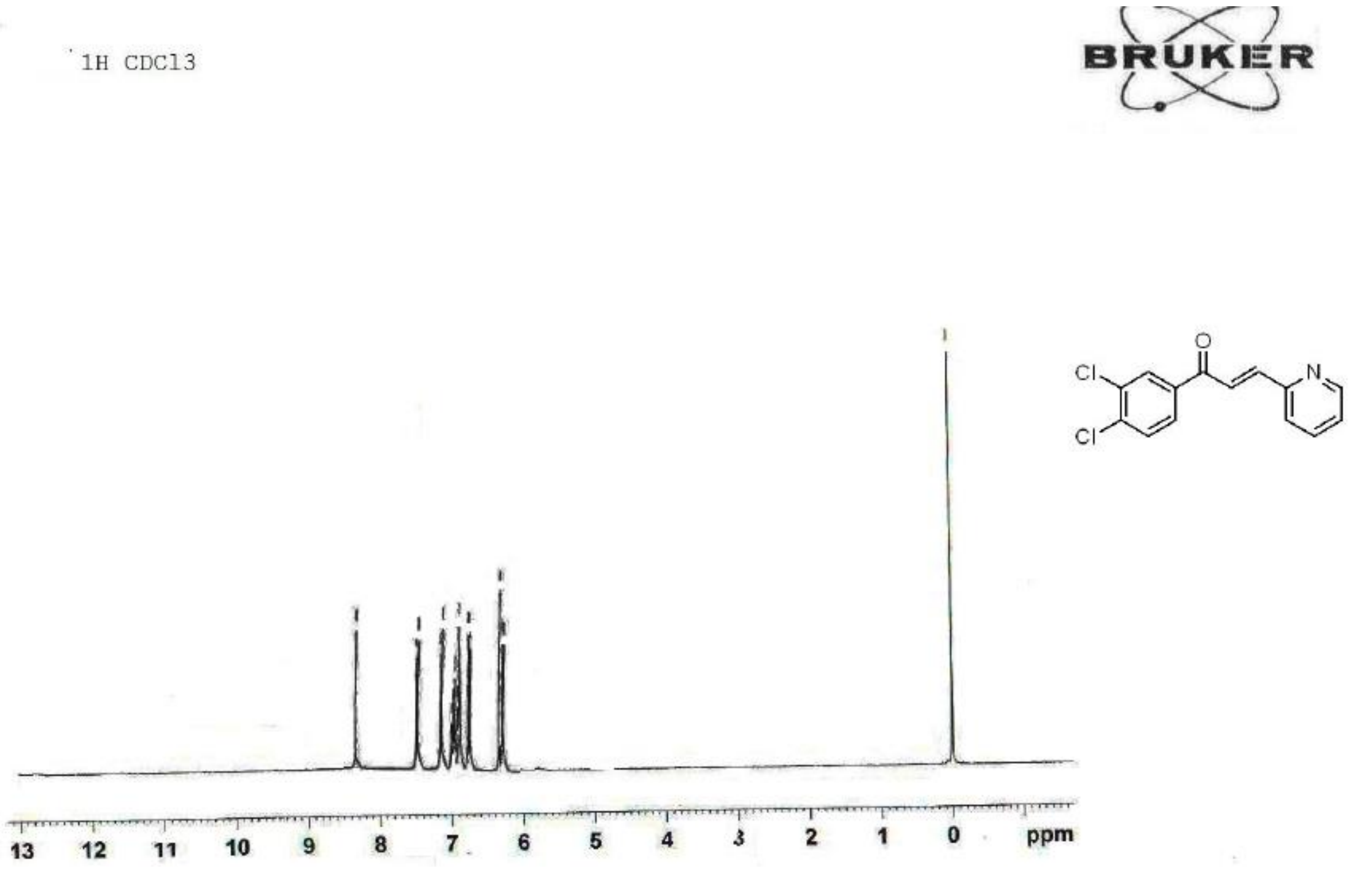

^{1}H-NMR spectrum of (*E*)-1-(3,4-dichlorophenyl)-3-(pyridin-2-yl)prop-2-en-1-one (**11**).

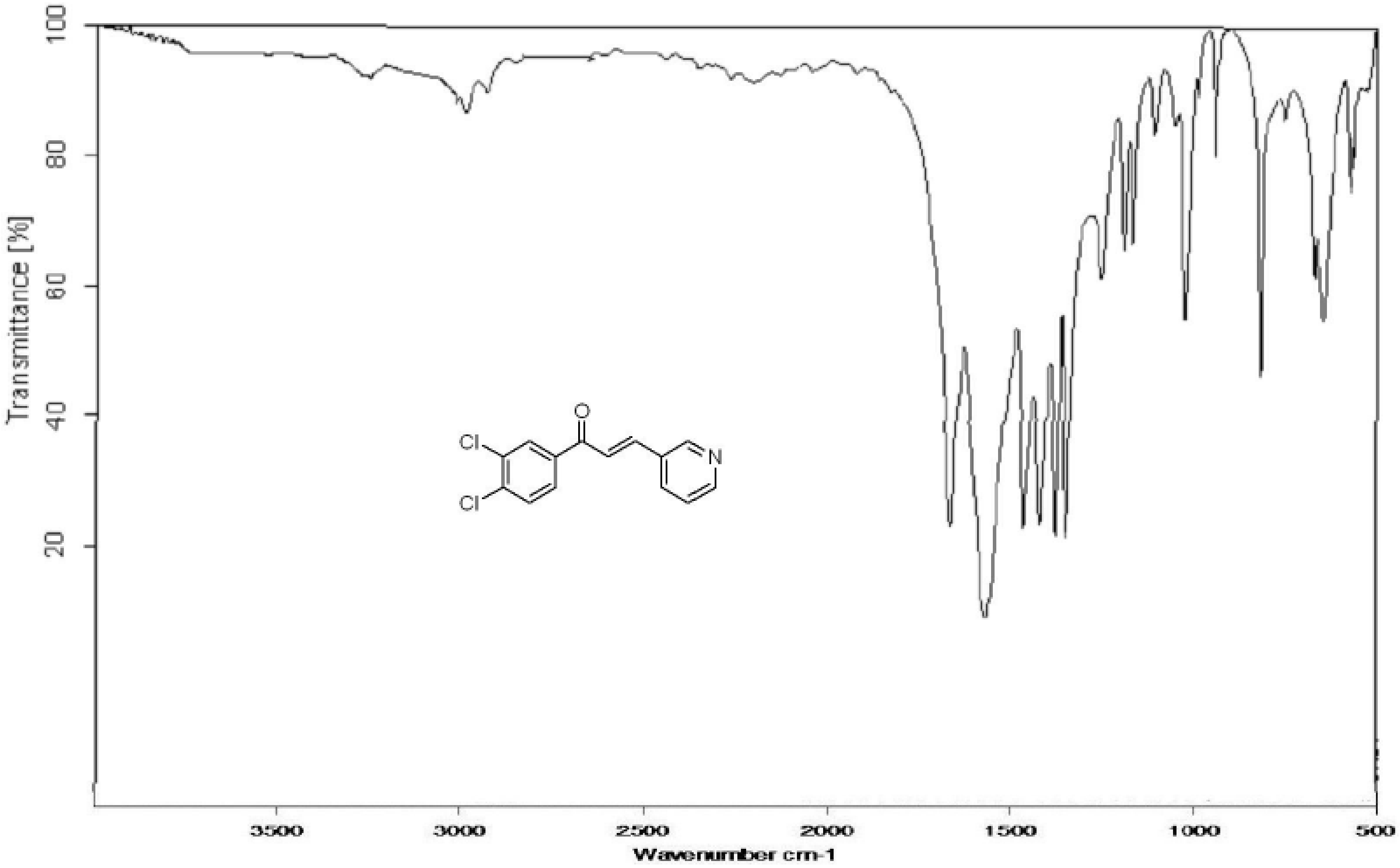

IR spectrum of (*E*)-1-(3,4-dichlorophenyl)-3-(pyridin-3-yl)prop-2-en-1-one (**12**).

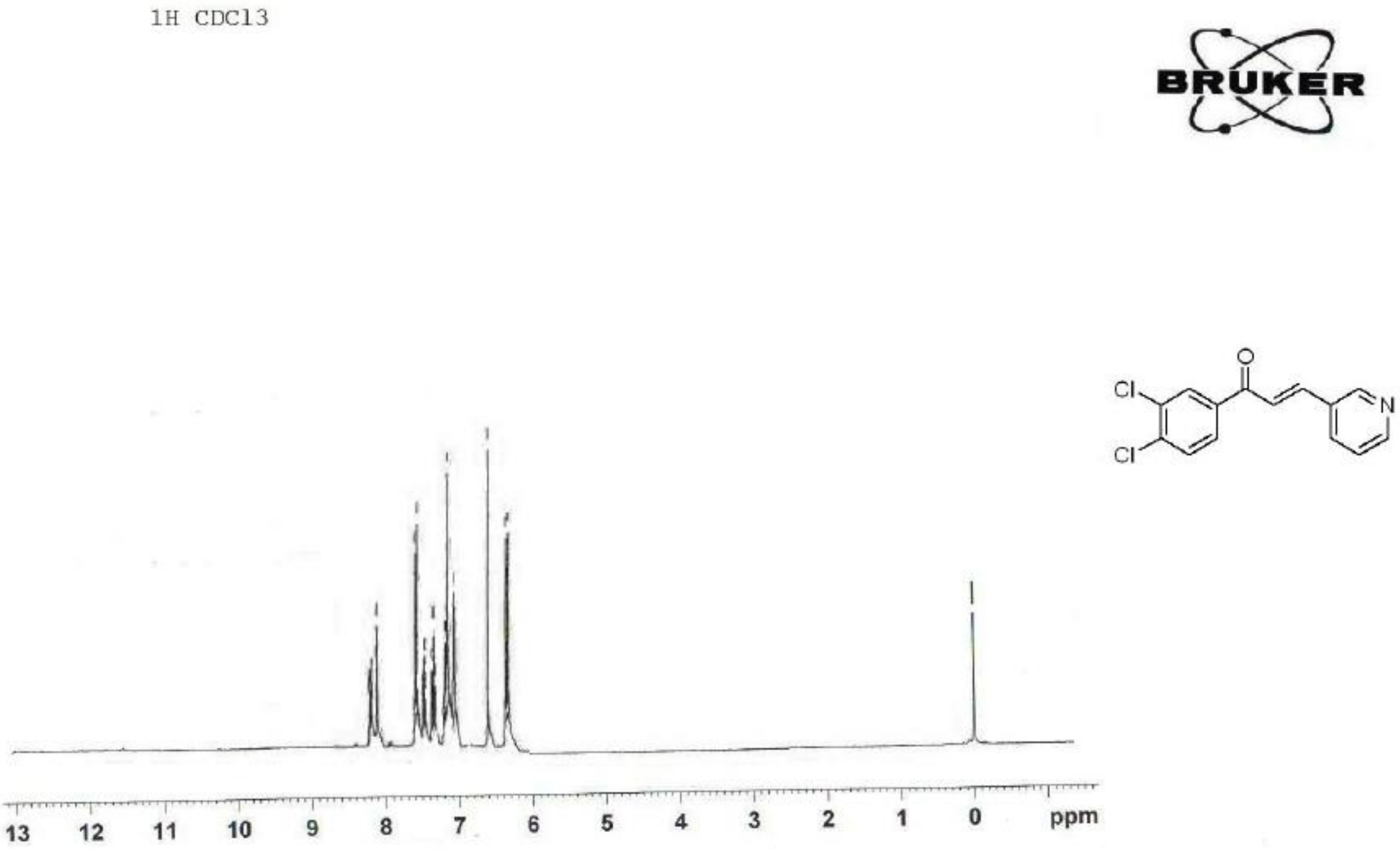

^{1}H-NMR spectrum of (*E*)-1-(3,4-dichlorophenyl)-3-(pyridin-3-yl)prop-2-en-1-one (**12**).

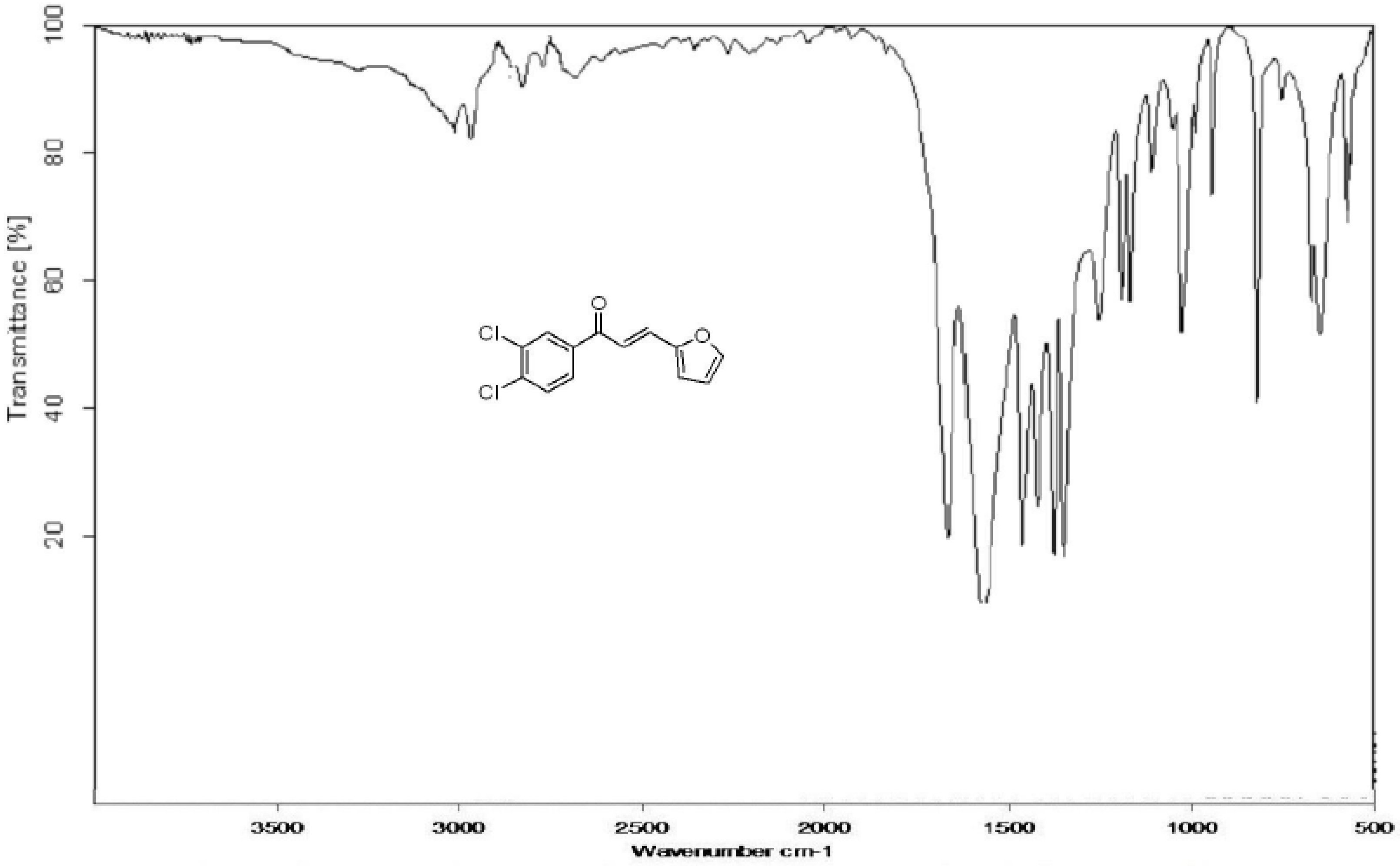

IR spectrum of (*E*)-1-(3,4-dichlorophenyl)-3-(furan-2-yl)prop-2-en-1-one (**14**).

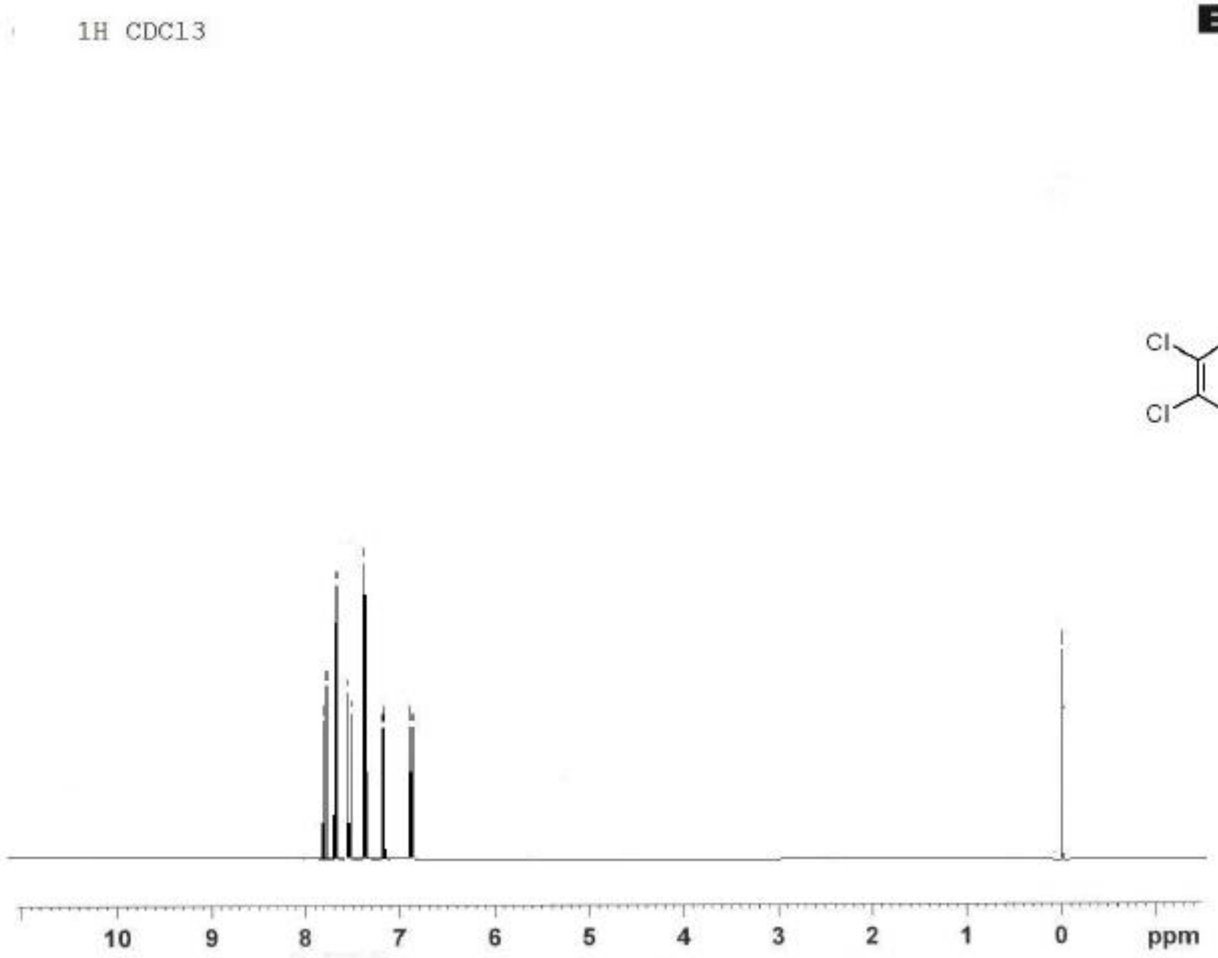

^{1}H-NMR spectrum of (*E*)-1-(3,4-dichlorophenyl)-3-(furan-2-yl)prop-2-en-1-one (**14**).

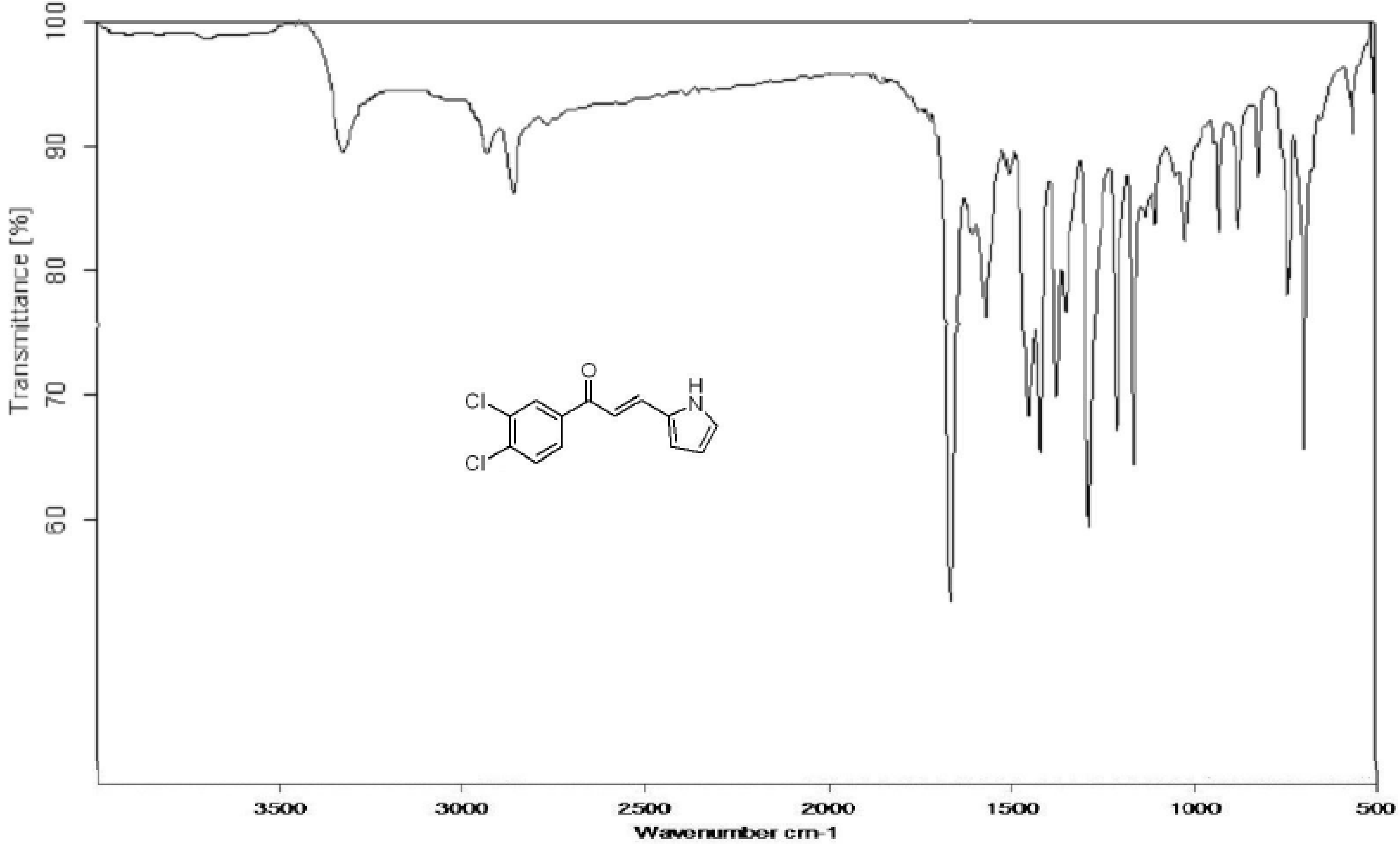

IR spectrum of (*E*)-1-(3,4-dichlorophenyl)-3-(1H-pyrrol-2-yl)prop-2-en-1-one (**15**).

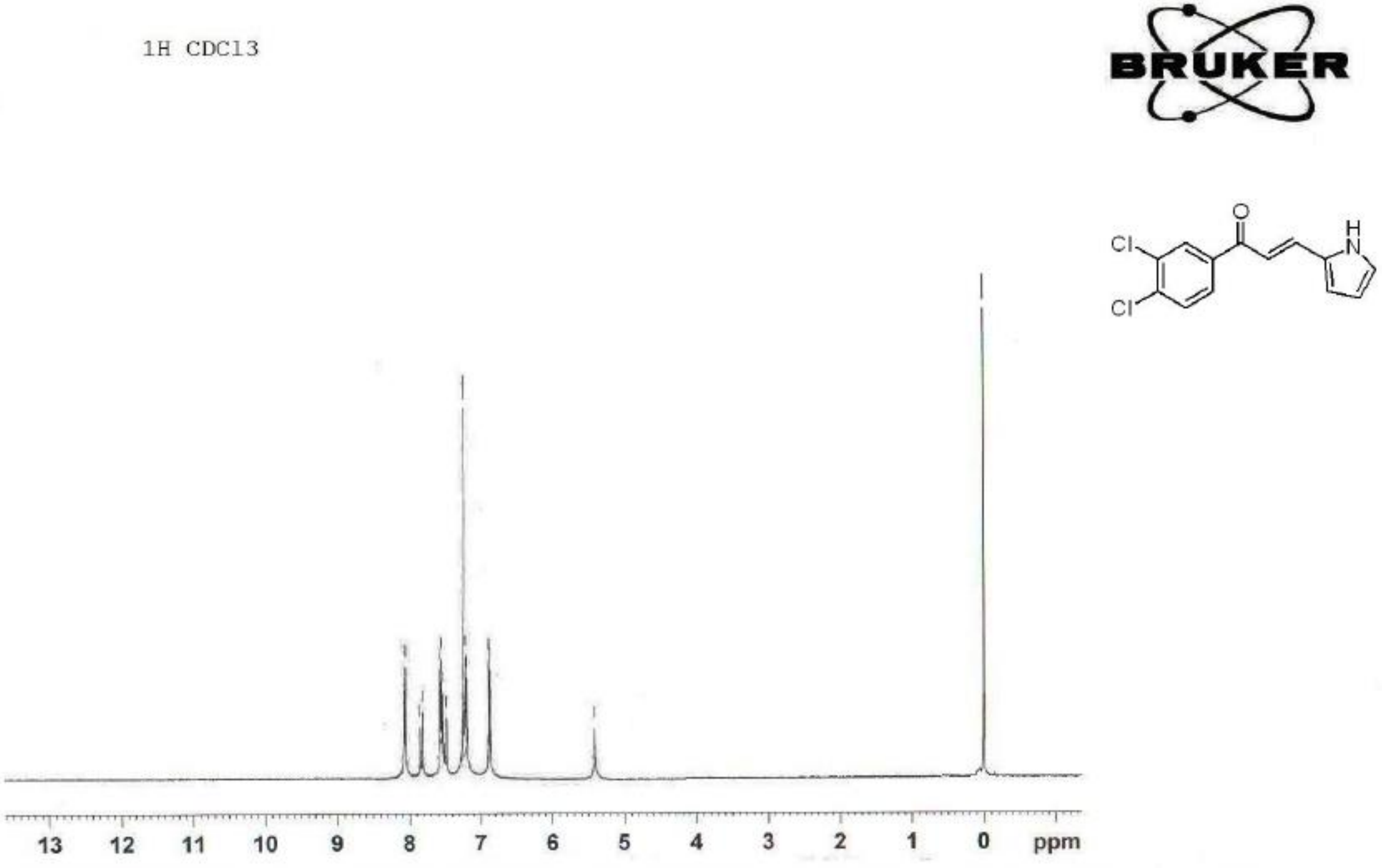

^{1}H-NMR spectrum of (*E*)-1-(3,4-dichlorophenyl)-3-(1H-pyrrol-2-yl)prop-2-en-1-one (**15**).

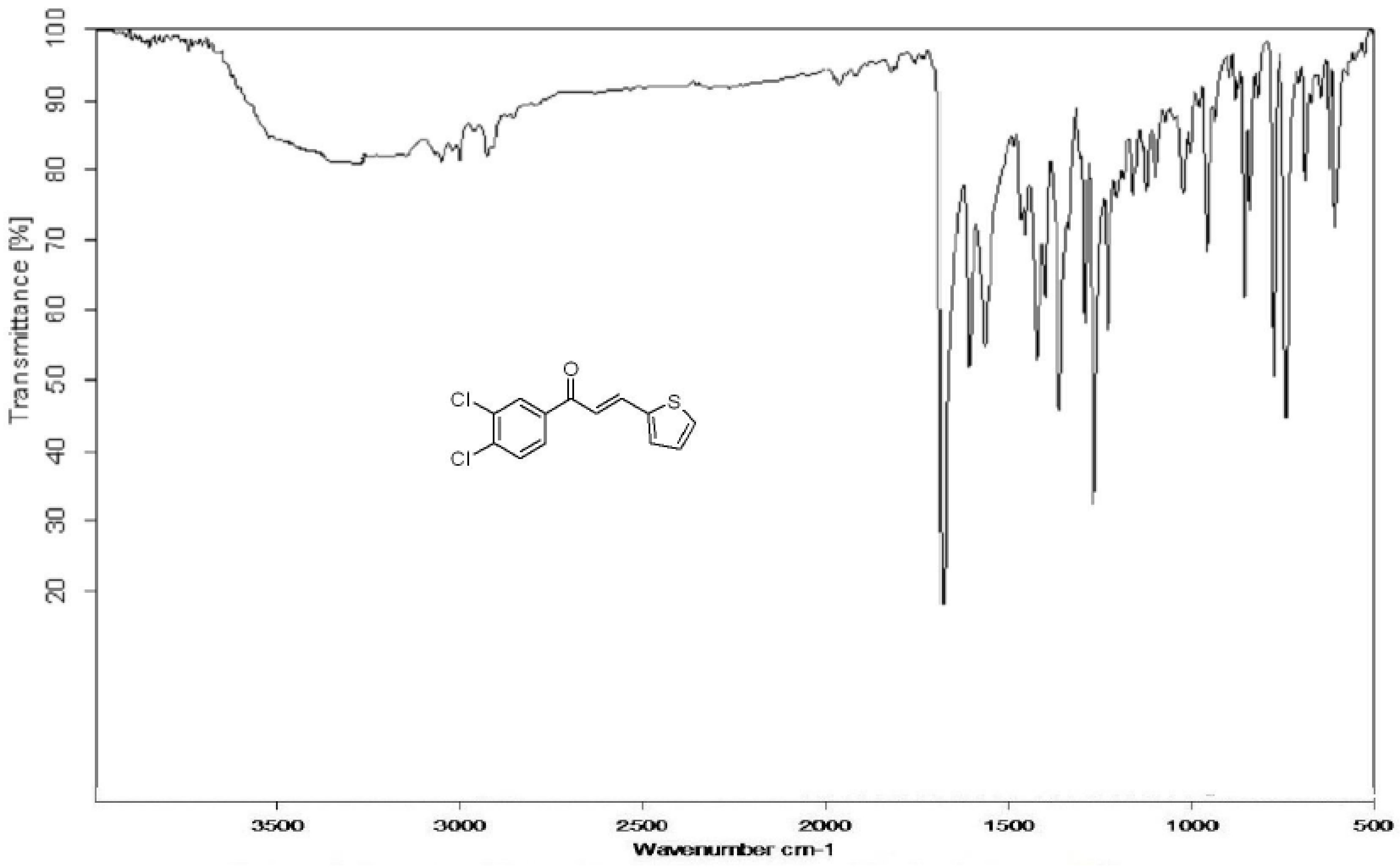

IR spectrum of (*E*)-1-(3,4-dichlorophenyl)-3-(thiophen-2-yl)prop-2-en-1-one (**16**).

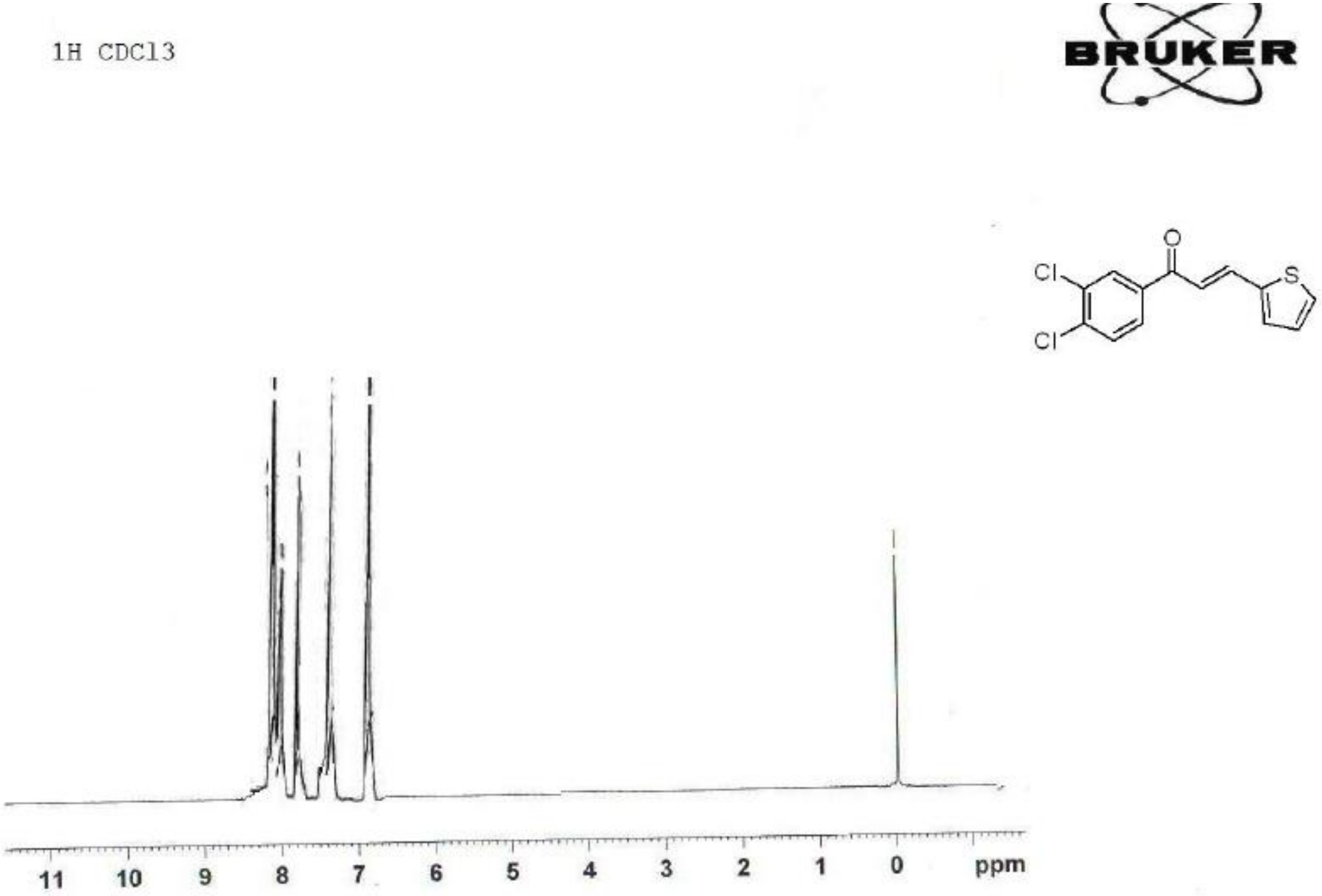

^{1}H-NMR spectrum of (*E*)-1-(3,4-dichlorophenyl)-3-(thiophen-2-yl)prop-2-en-1-one (**16**).

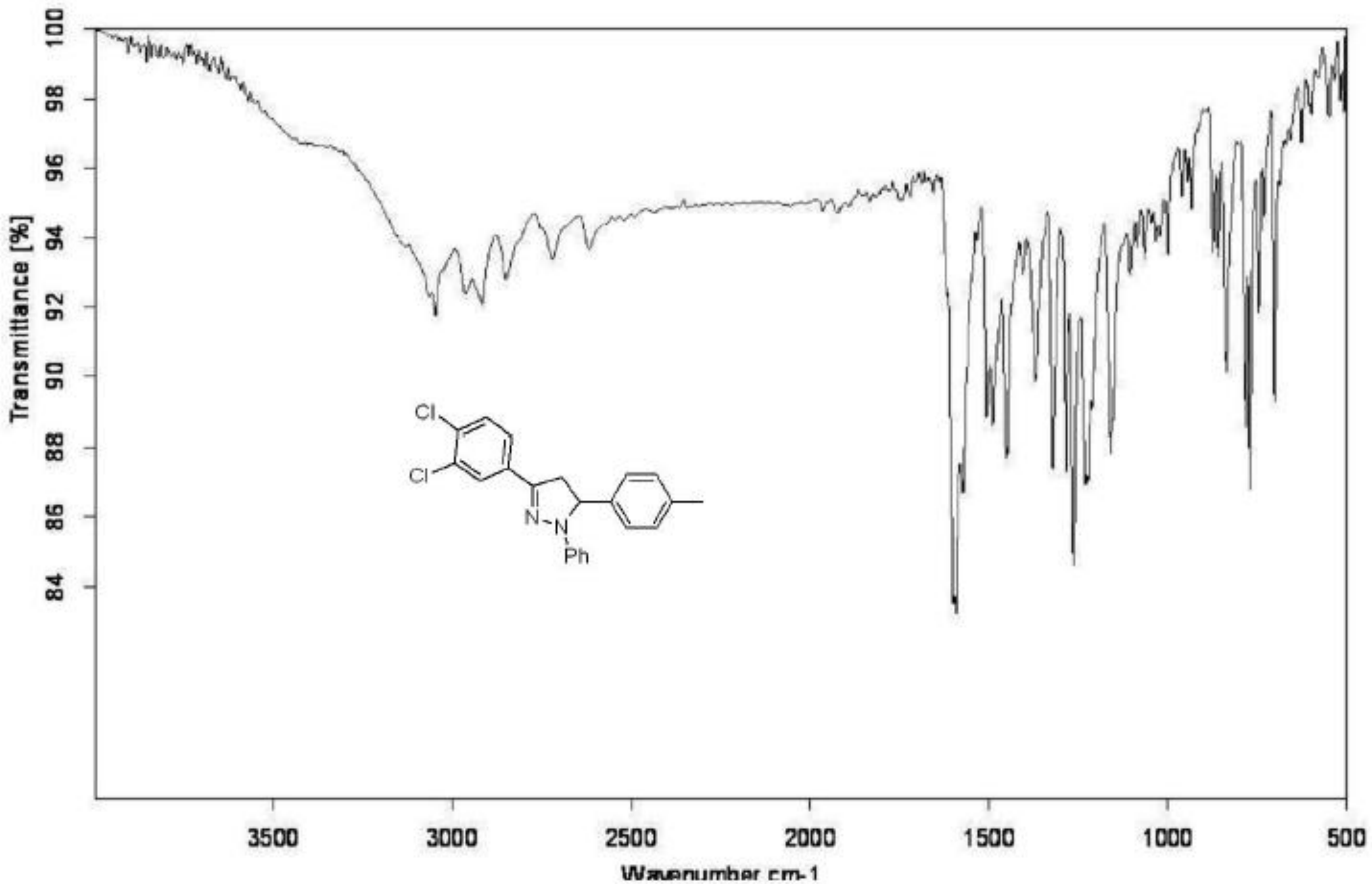

IR spectrum of 3-(3,4-dichlorophenyl)-1-phenyl-5-(p-tolyl)-4,5-dihydro-1H-pyrazole (**17**).

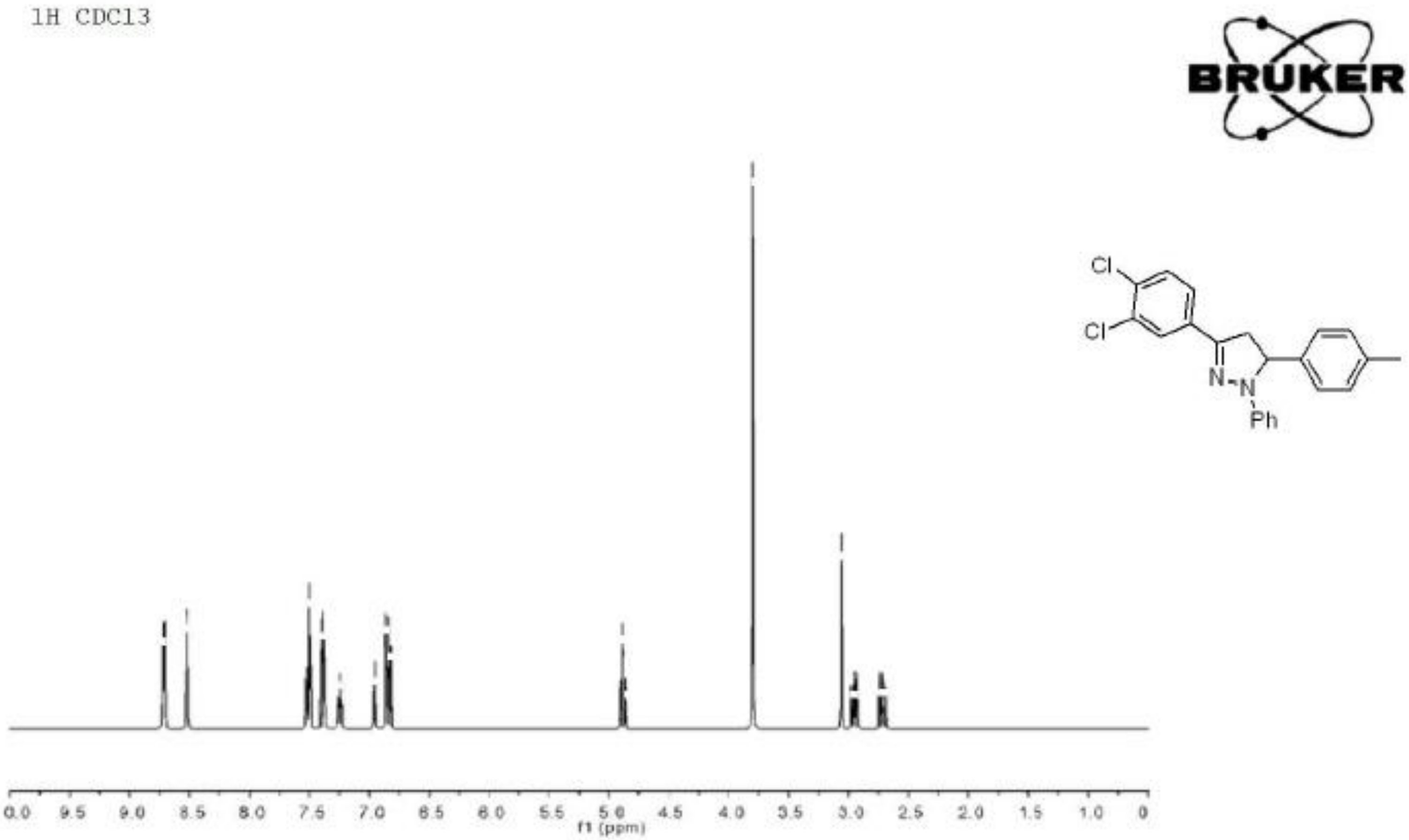

^{1}H-NMR spectrum of 3-(3,4-dichlorophenyl)-1-phenyl-5-(p-tolyl)-4,5-dihydro-1H-pyrazole (**17**).

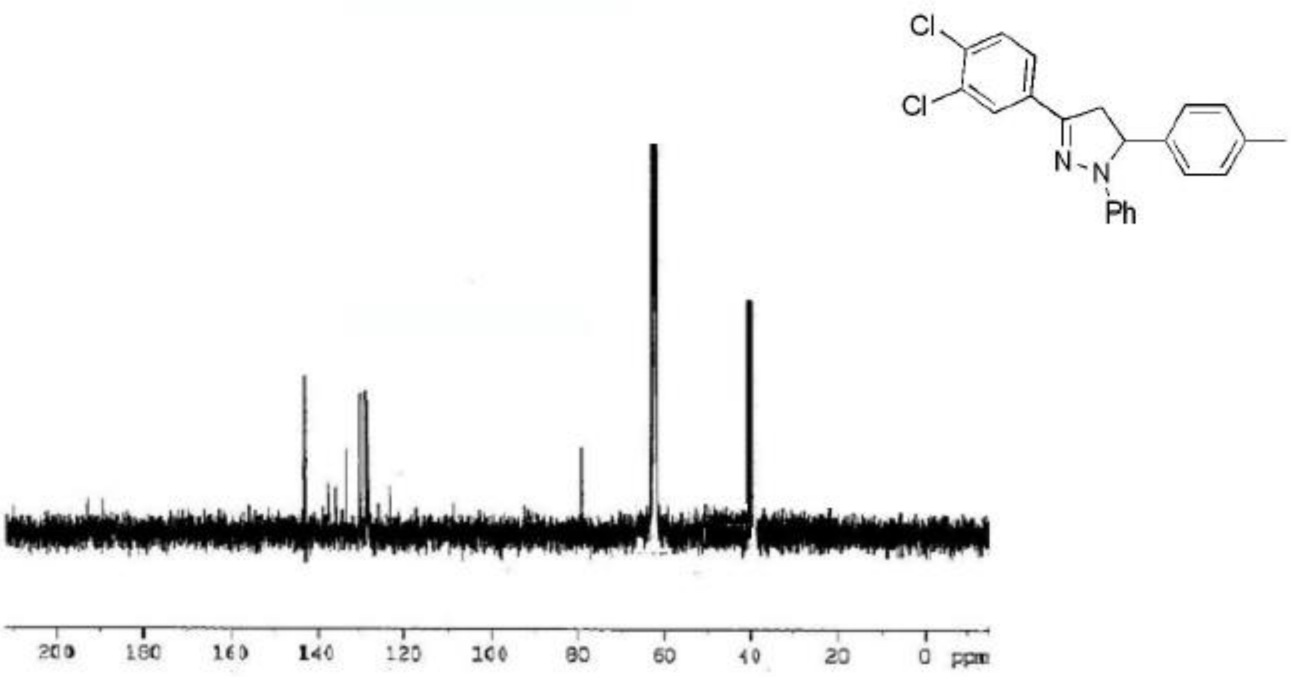

^{13}C-NMR spectrum of 3-(3,4-dichlorophenyl)-1-phenyl-5-(p-tolyl)-4,5-dihydro-1H-pyrazole (**17**).

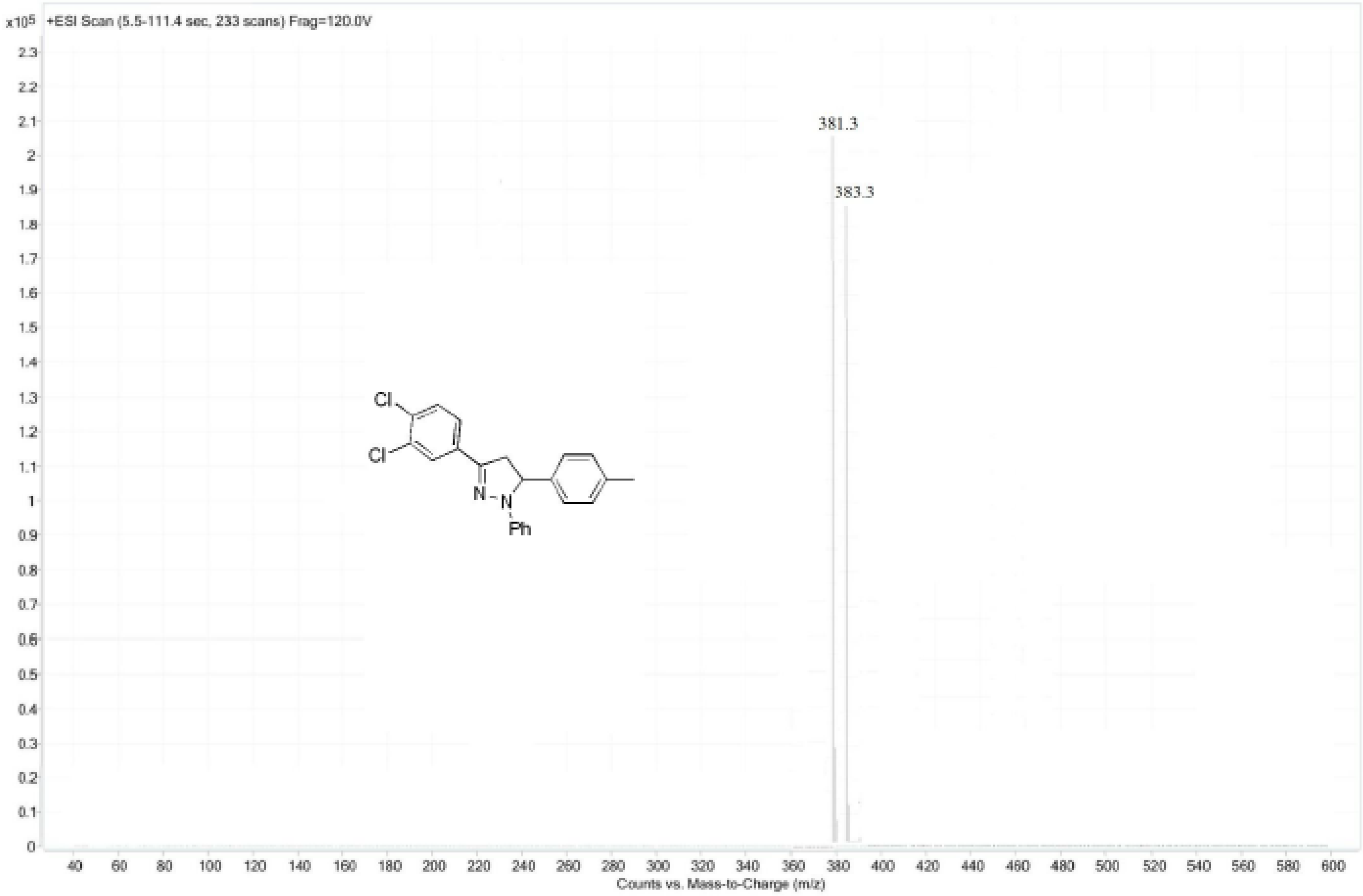

Mass spectrum of 3-(3,4-dichlorophenyl)-1-phenyl-5-(p-tolyl)-4,5-dihydro-1H-pyrazole (**17**).

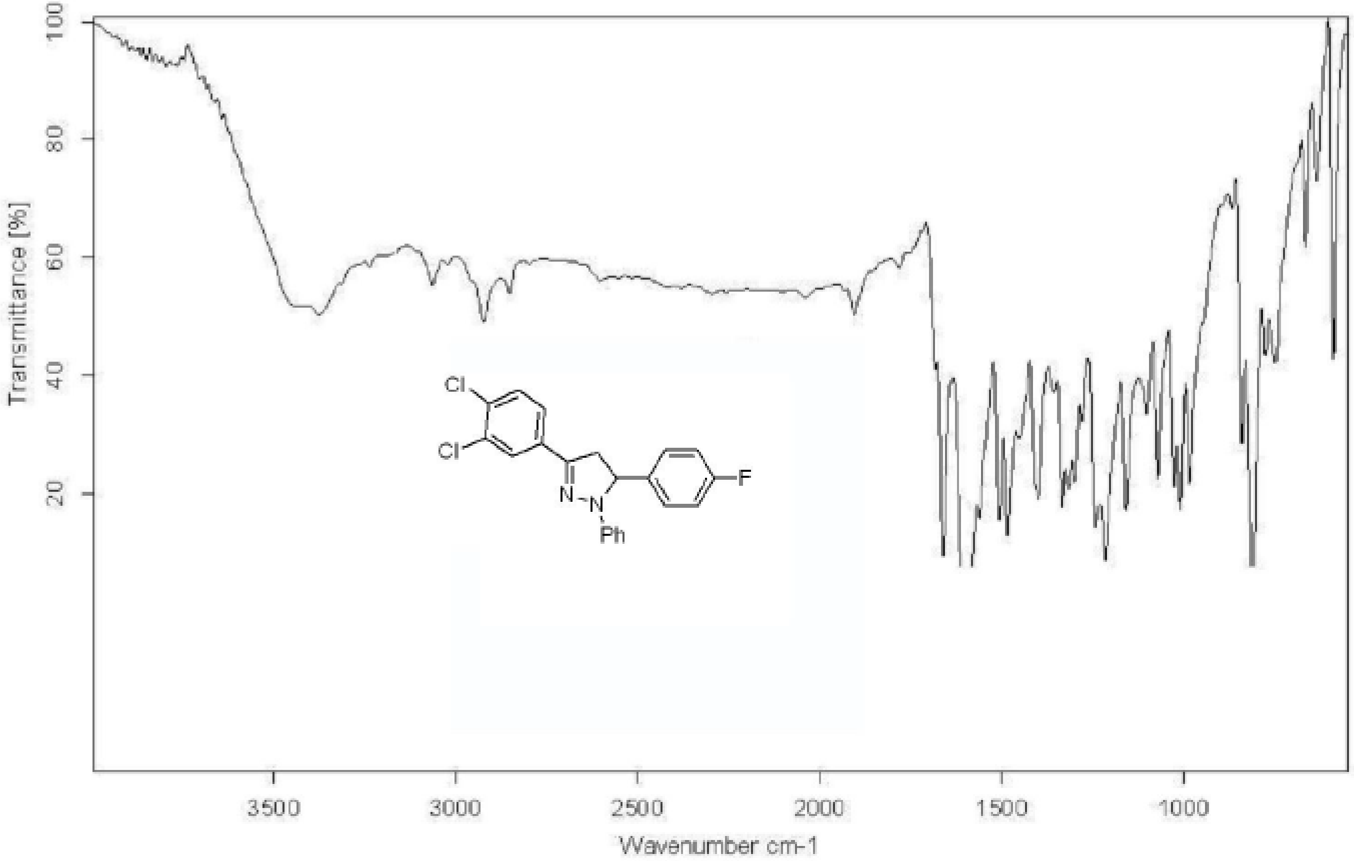

IR spectrum of 3-(3,4-dichlorophenyl)-1-phenyl-5-(4-fluorophenyl)-4,5-dihydro-1H-pyrazole (**18**).

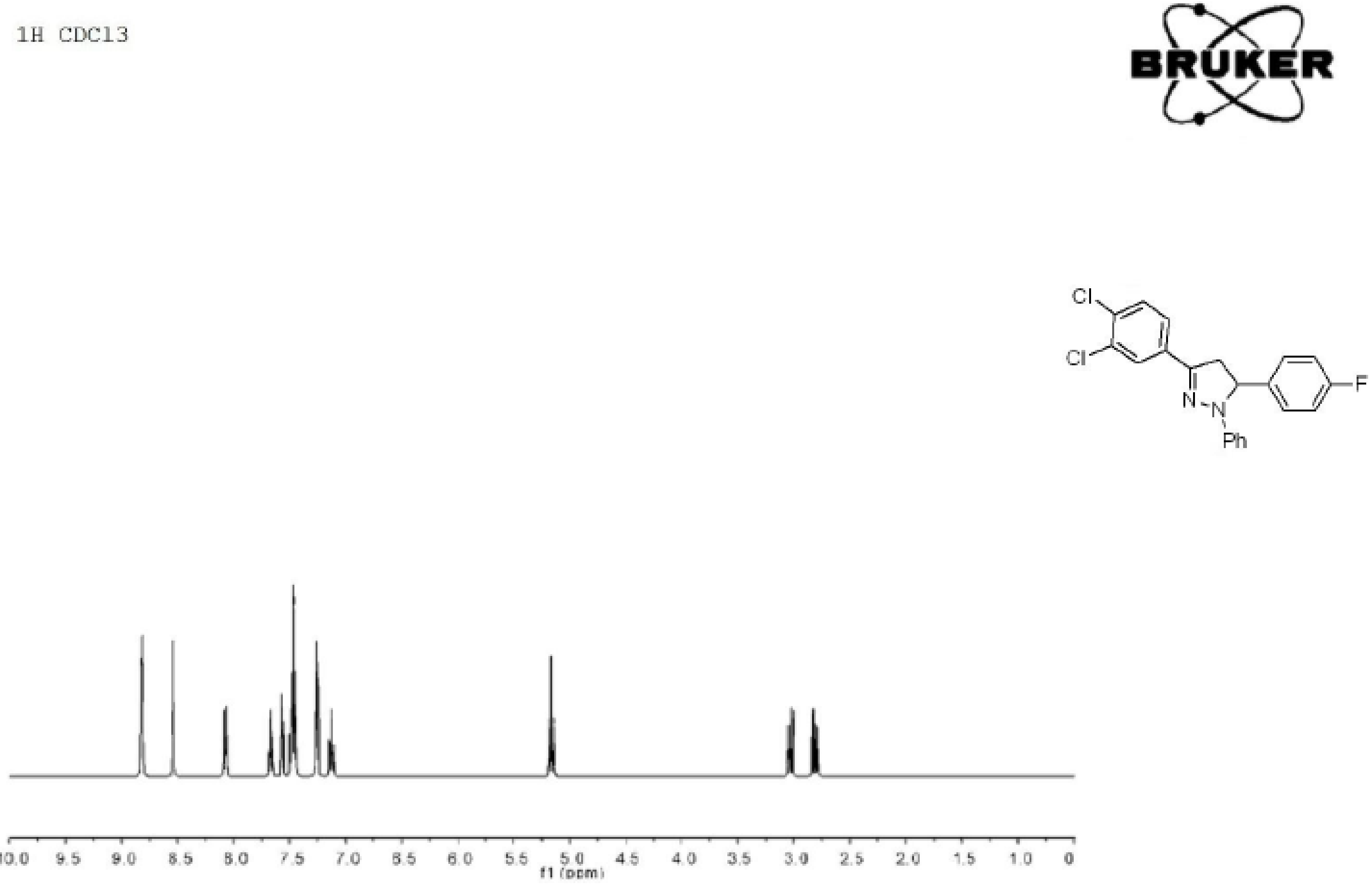

^{1}H-NMR spectrum of 3-(3,4-dichlorophenyl)-1-phenyl-5-(4-fluorophenyl)-4,5-dihydro-1H-pyrazole (**18**).

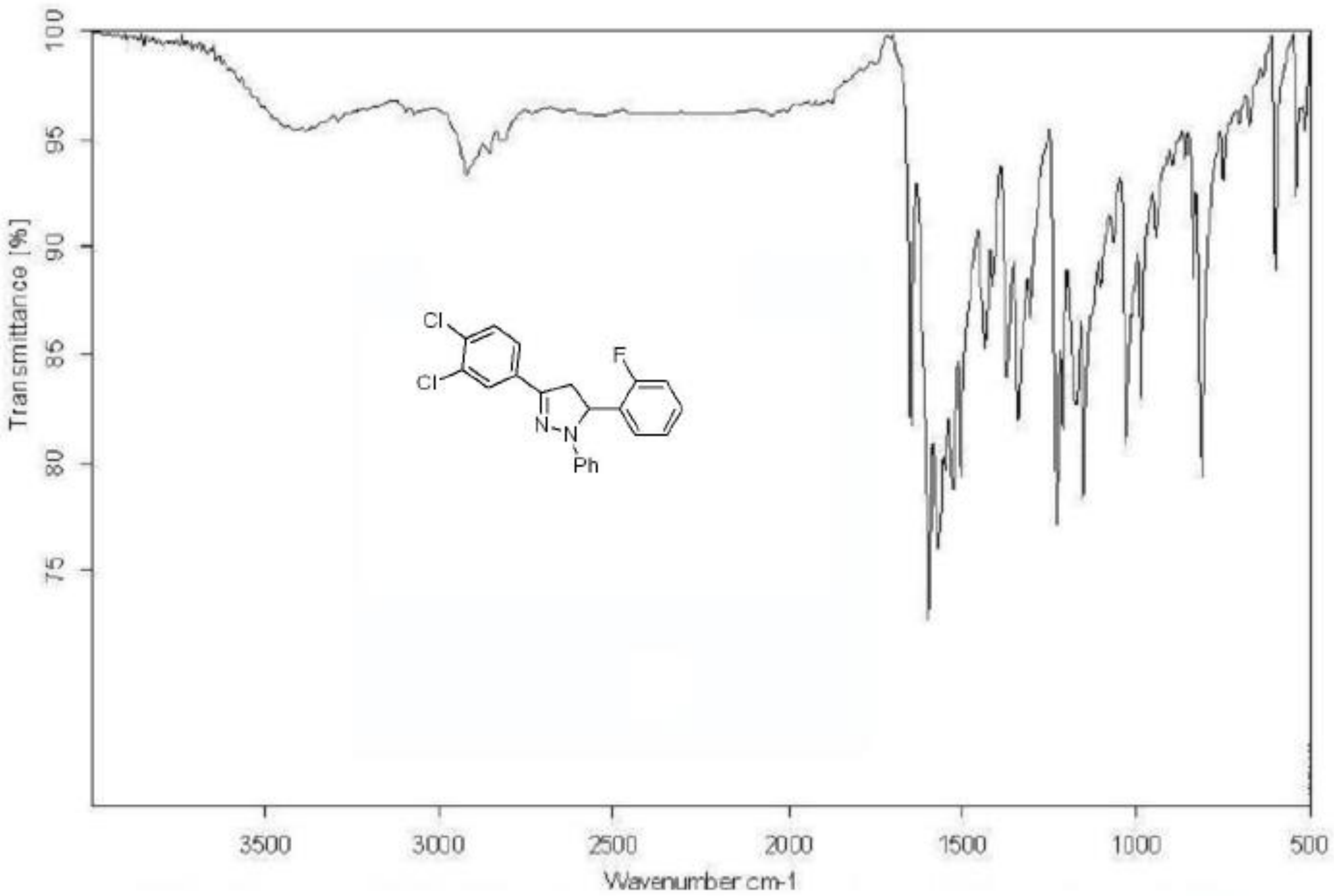

IR spectrum of 3-(3,4-dichlorophenyl)-1-phenyl-5-(2-fluorophenyl)-4,5-dihydro-1H-pyrazole (**19**).

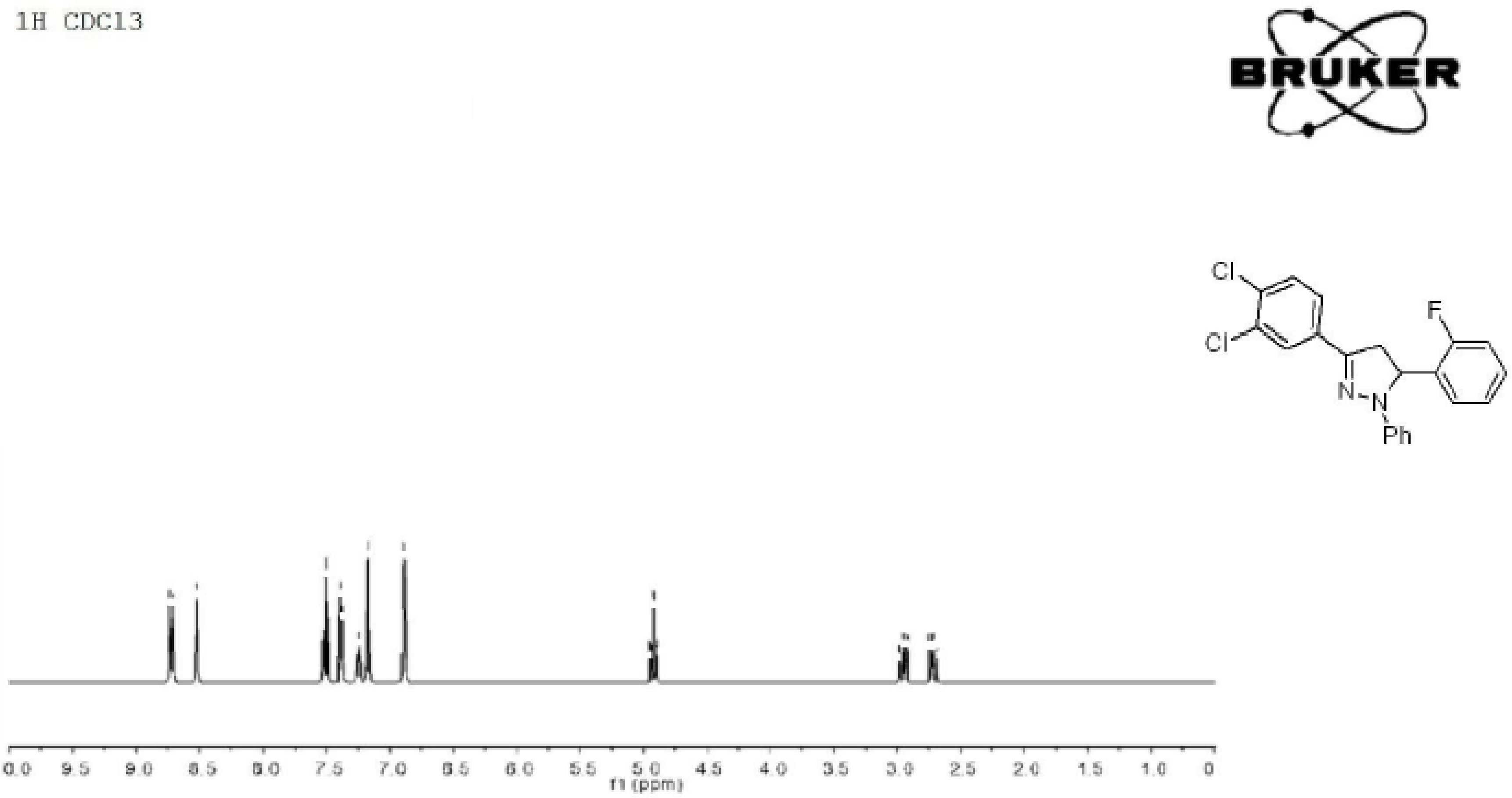

^{1}H-NMR spectrum of 3-(3,4-dichlorophenyl)-1-phenyl-5-(2-fluorophenyl)-4,5-dihydro-1H-pyrazole (**19**).

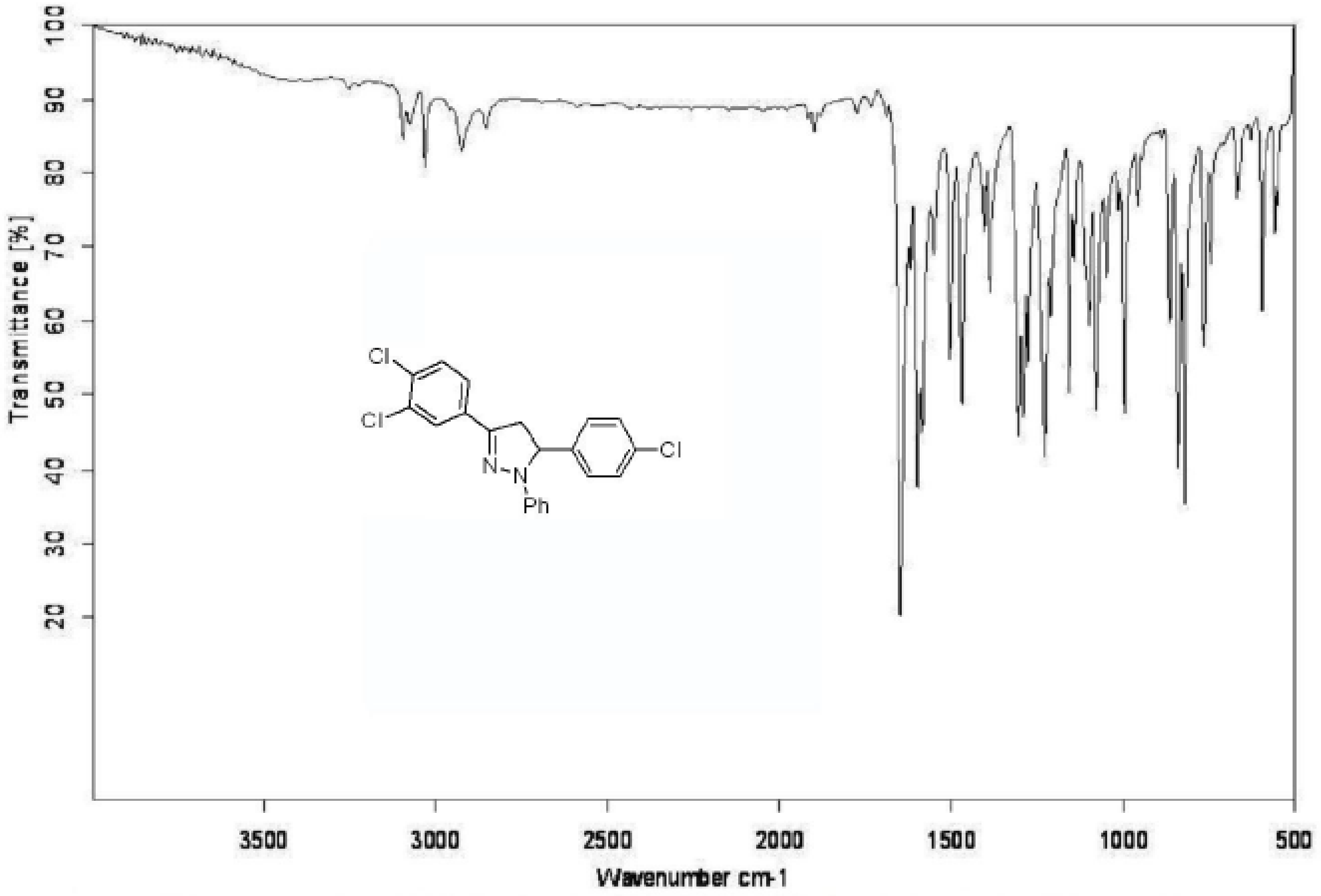

IR spectrum of 3-(3,4-dichlorophenyl)-1-phenyl-5-(4-chlorophenyl)-4,5-dihydro-1H-pyrazole (**20**).

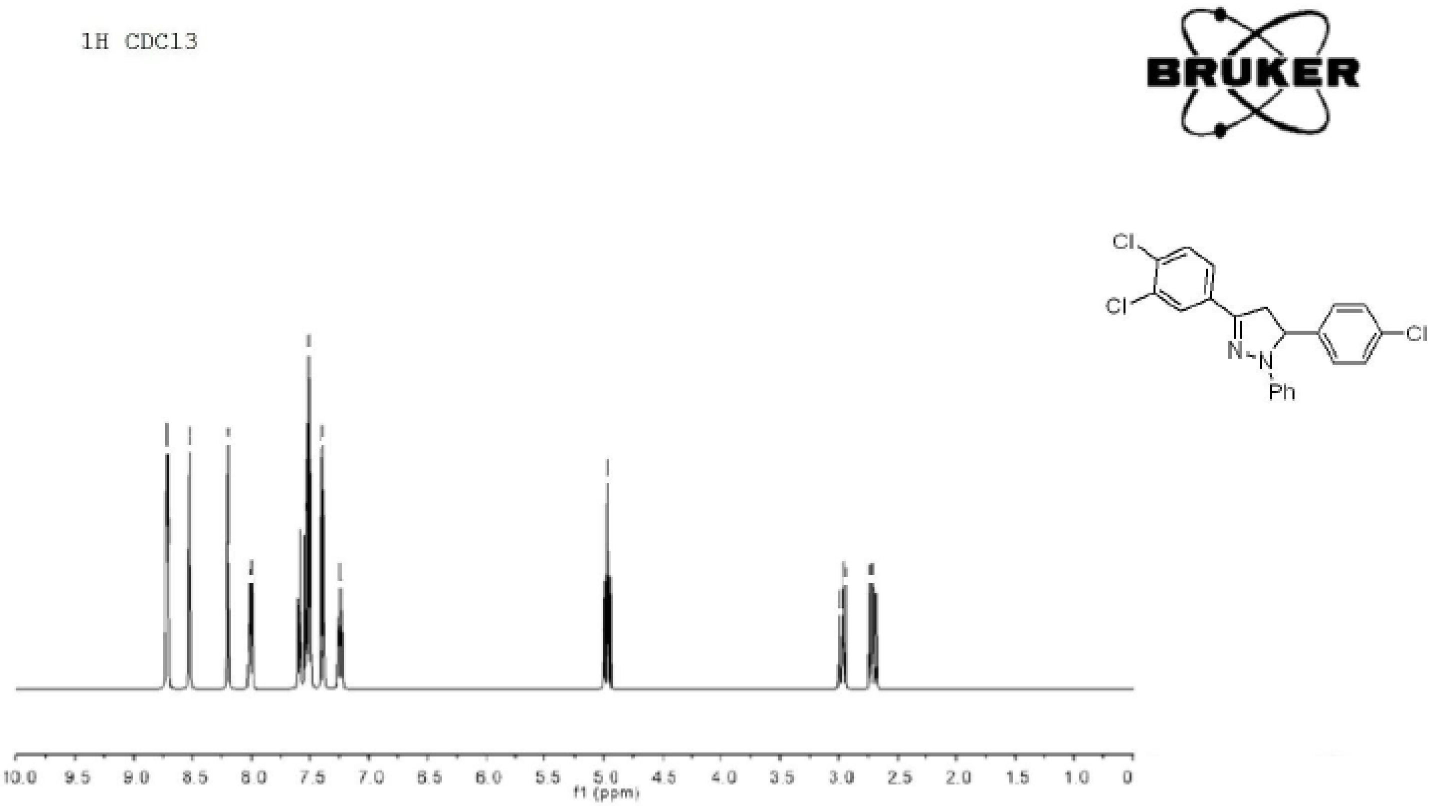

^{1}H-NMR spectrum of 3-(3,4-dichlorophenyl)-1-phenyl-5-(4-chlorophenyl)-4,5-dihydro-1H-pyrazole (**20**).

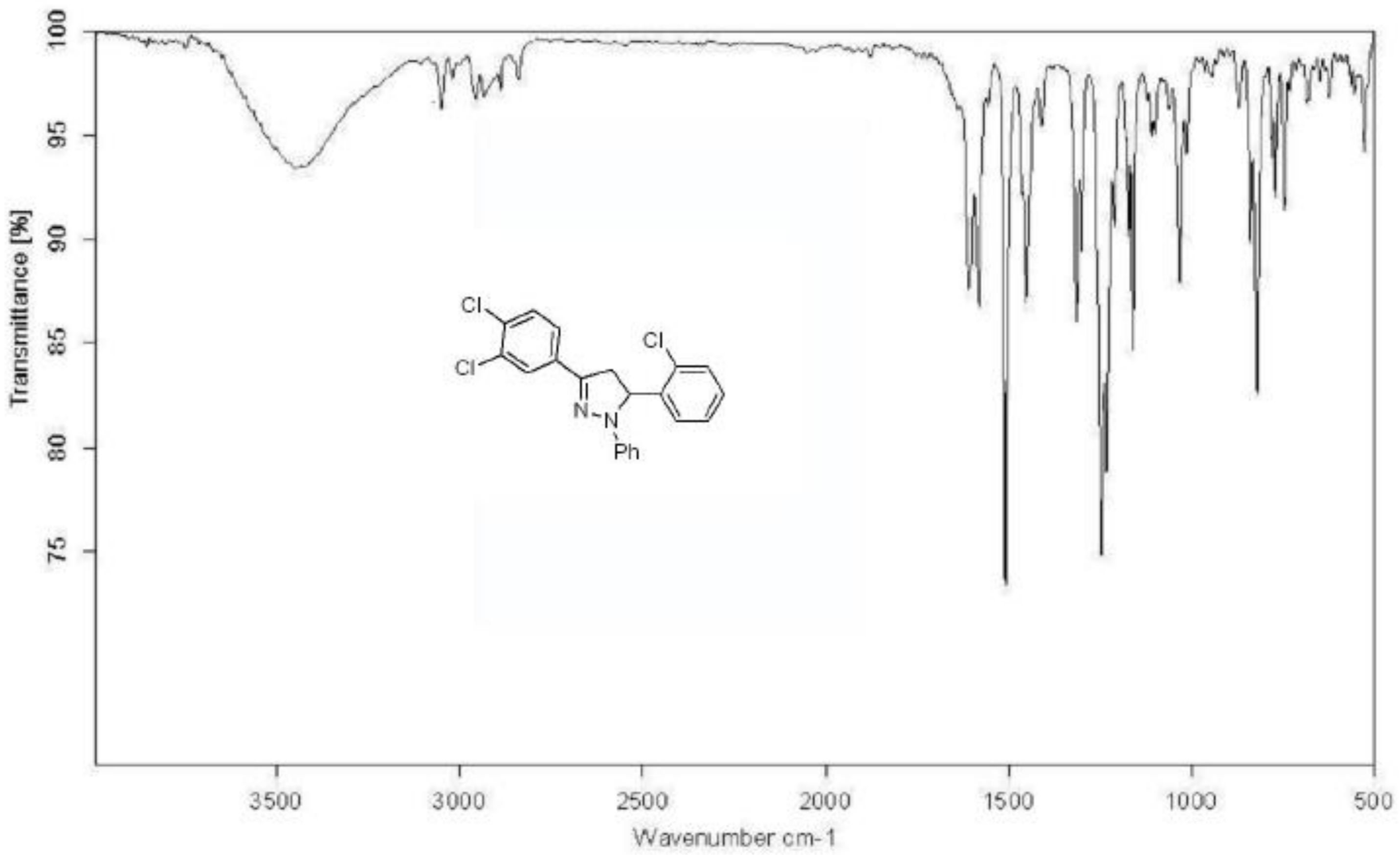

IR spectrum of 3-(3,4-dichlorophenyl)-1-phenyl-5-(2-chlorophenyl)-4,5-dihydro-1H-pyrazole (**21**).

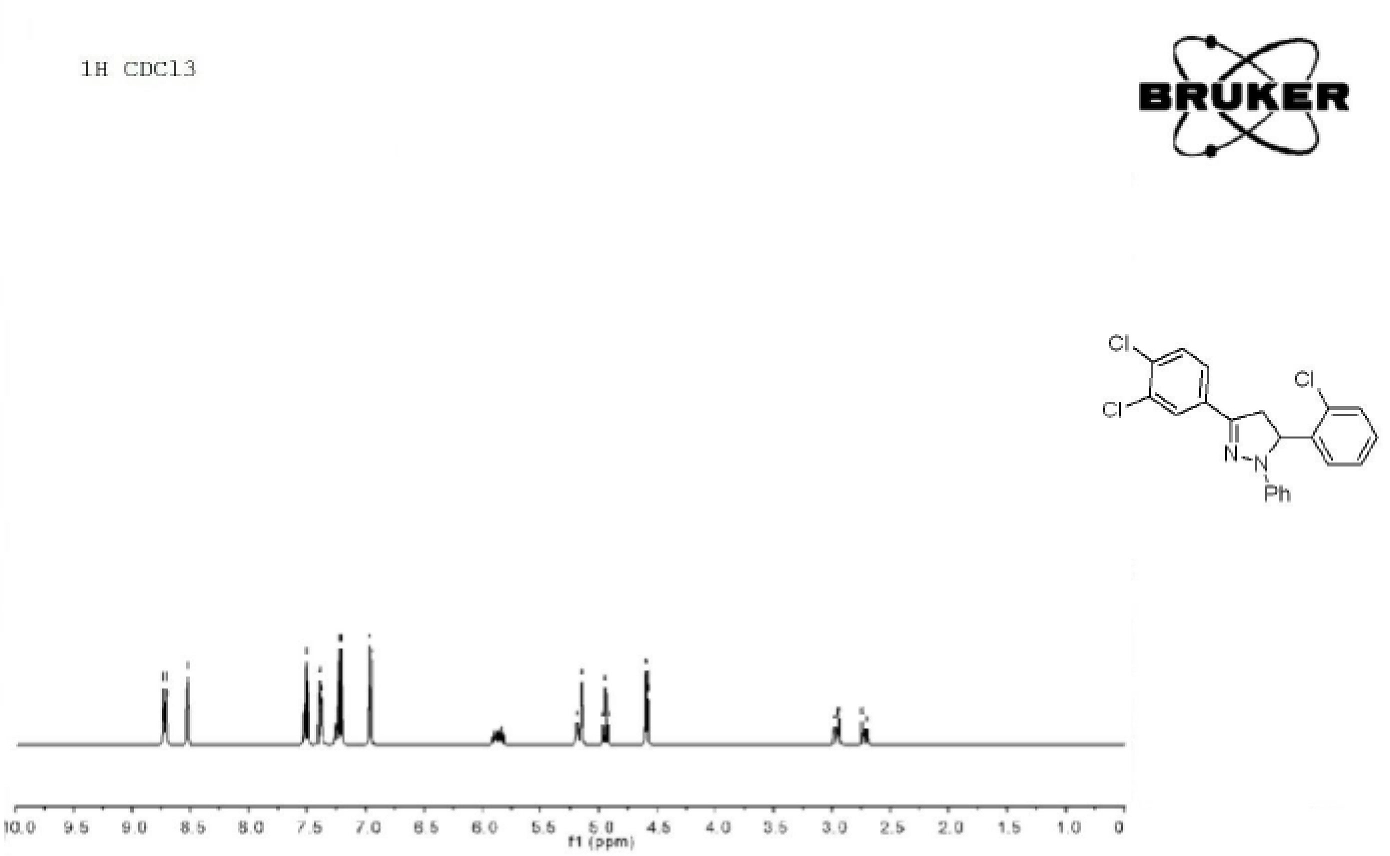

^{1}H-NMR spectrum of 3-(3,4-dichlorophenyl)-1-phenyl-5-(2-chlorophenyl)-4,5-dihydro-1H-pyrazole (**21**).

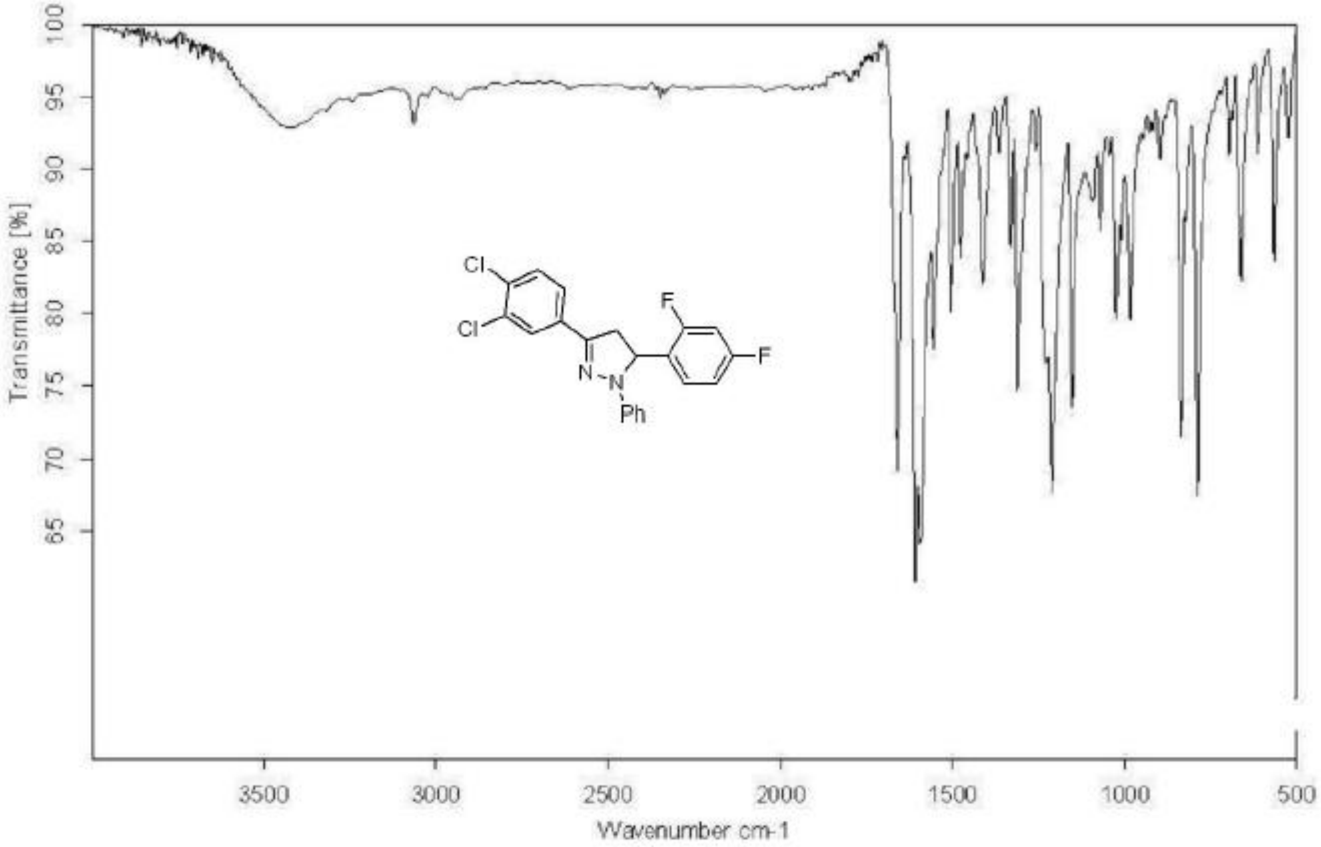

IR spectrum of 3-(3,4-dichlorophenyl)-1-phenyl-5-(2,4-difluorophenyl)-4,5-dihydro-1H-pyrazole (**22**).

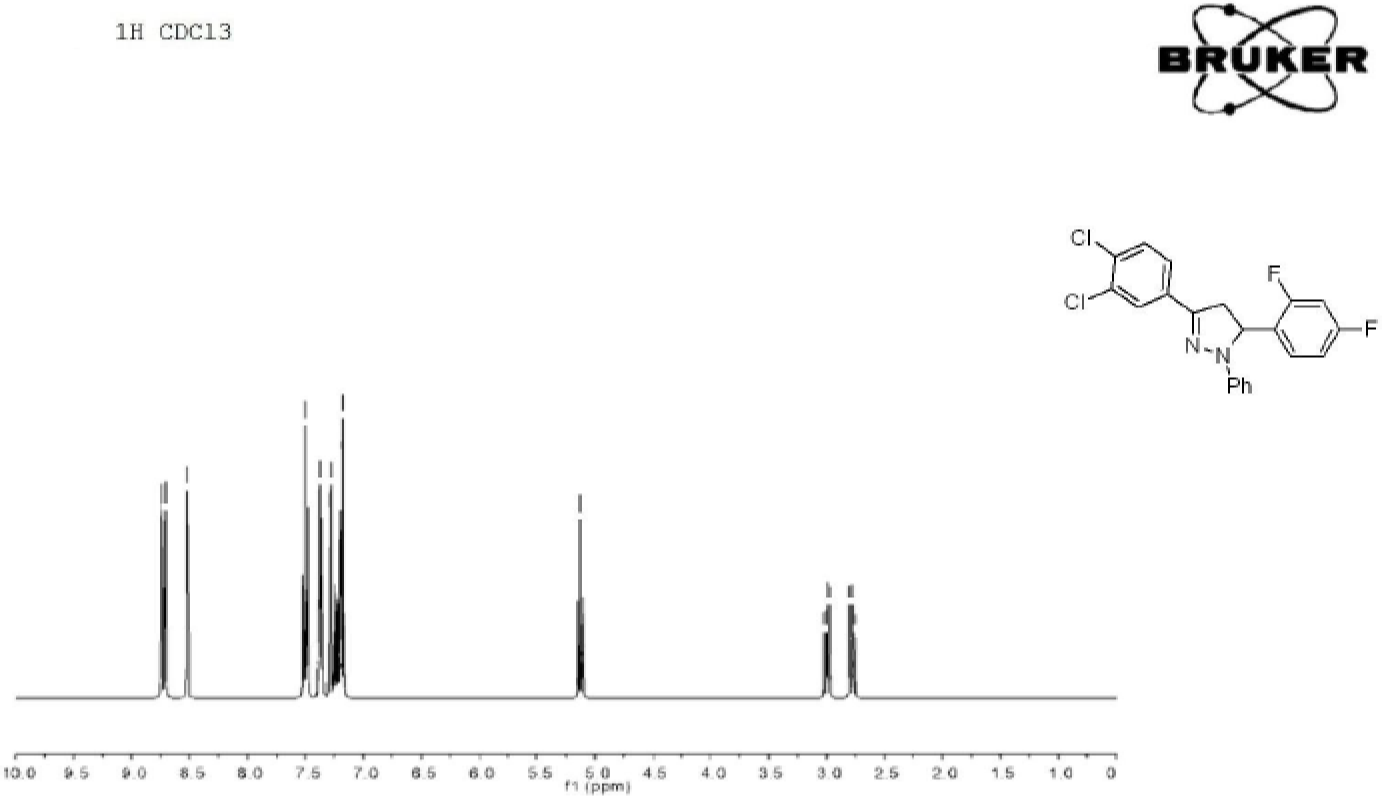

^{1}H-NMR spectrum of 3-(3,4-dichlorophenyl)-1-phenyl-5-(2,4-difluorophenyl)-4,5-dihydro-1H-pyrazole (**22**).

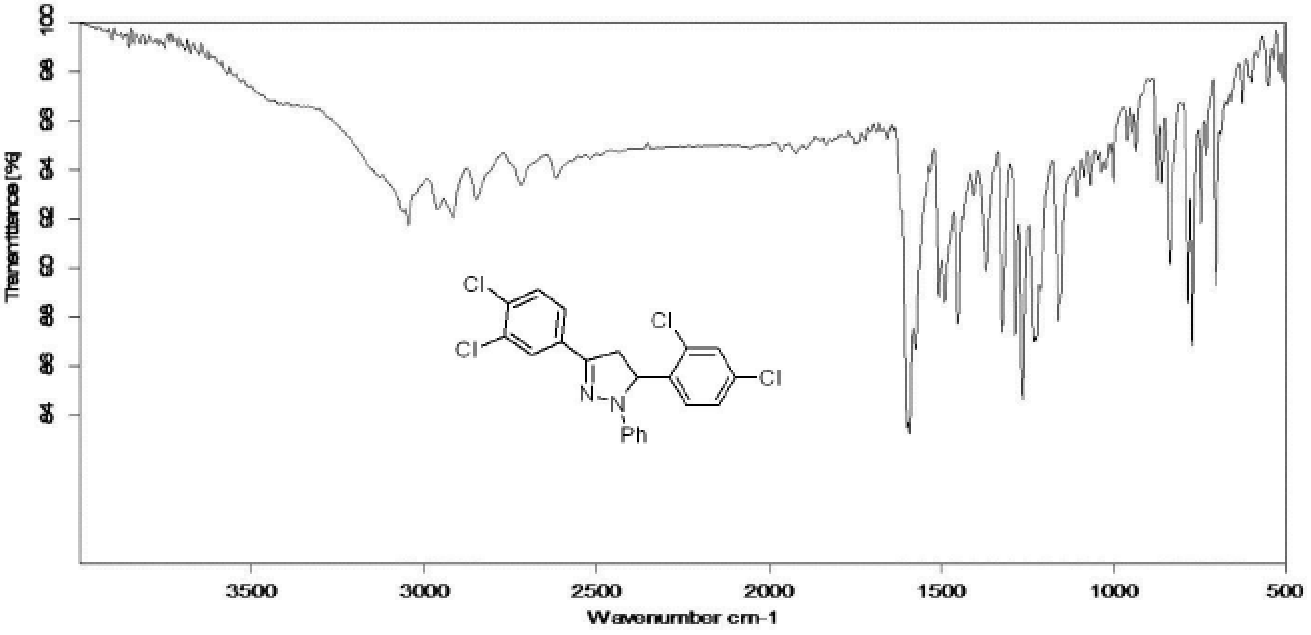

IR spectrum of 3-(3,4-dichlorophenyl)-1-phenyl-5-(2,4-dichlorophenyl)-4,5-dihydro-1H-pyrazole (**23**).

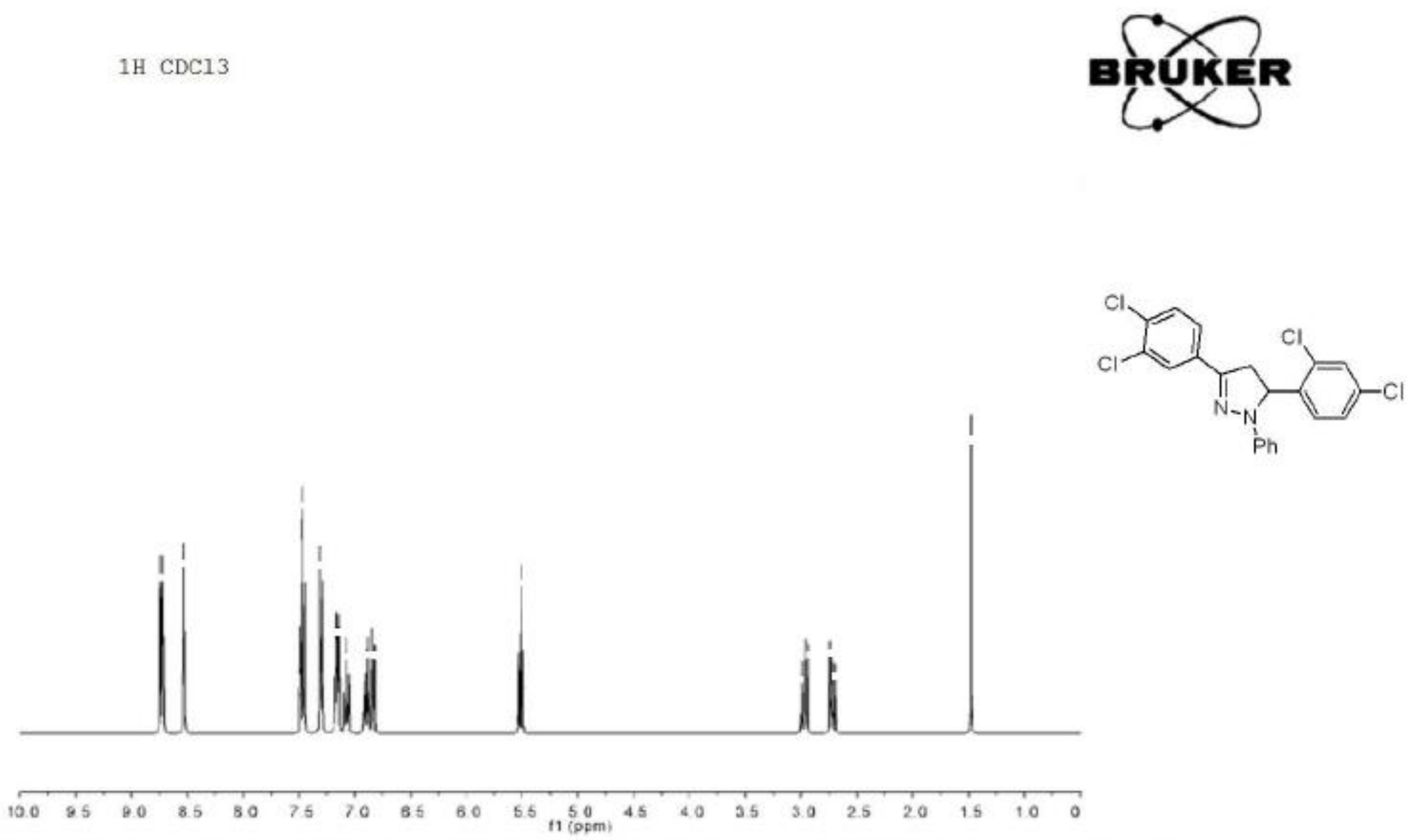

^{1}H-NMR spectrum of 3-(3,4-dichlorophenyl)-1-phenyl-5-(2,4-dichlorophenyl)-4,5-dihydro-1H-pyrazole (**23**).

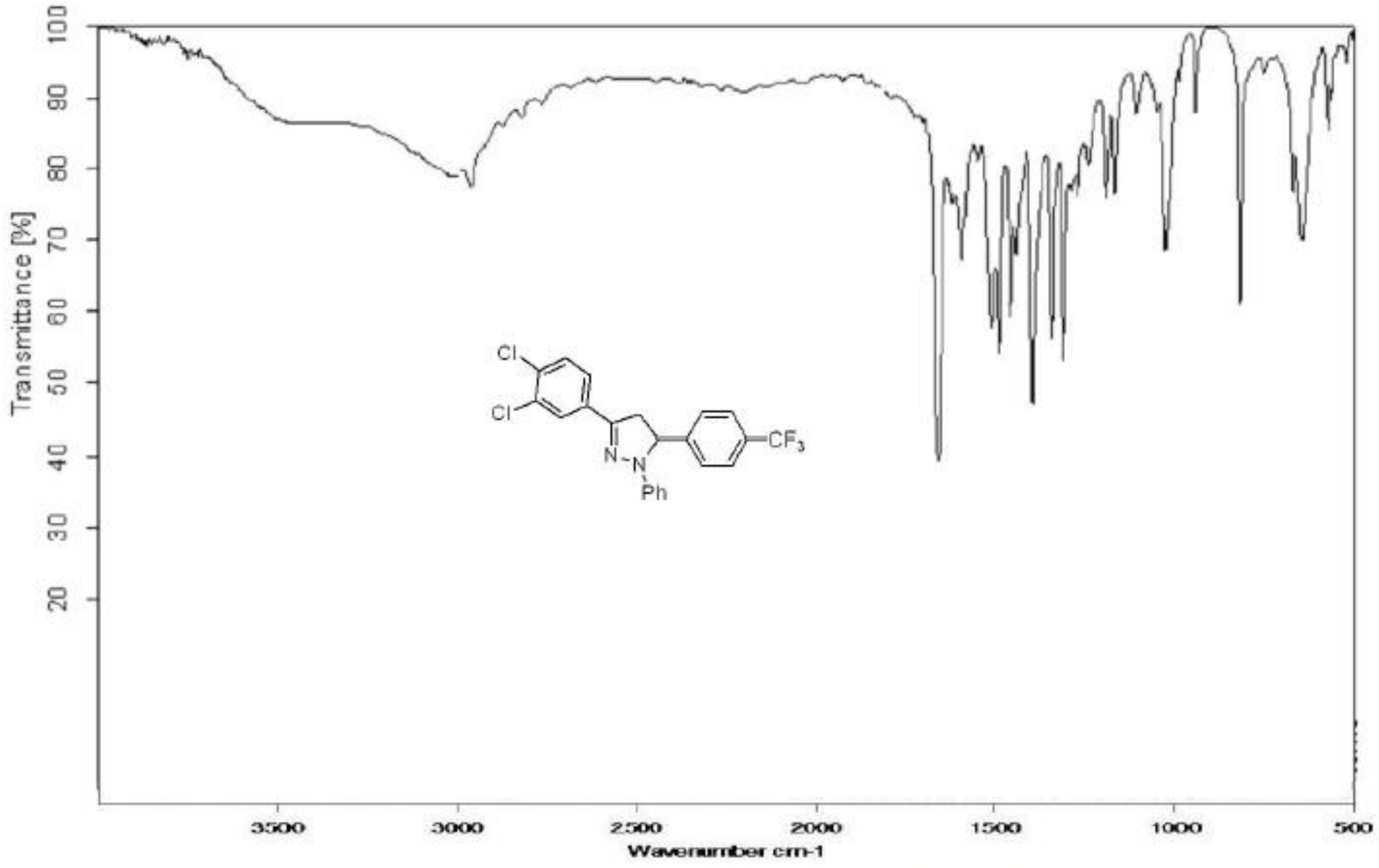

IR spectrum of 3-(3,4-dichlorophenyl)-1-phenyl-5-(4-trifluoromethylphenyl)-4,5-dihydro-1H-pyrazole (**24**).

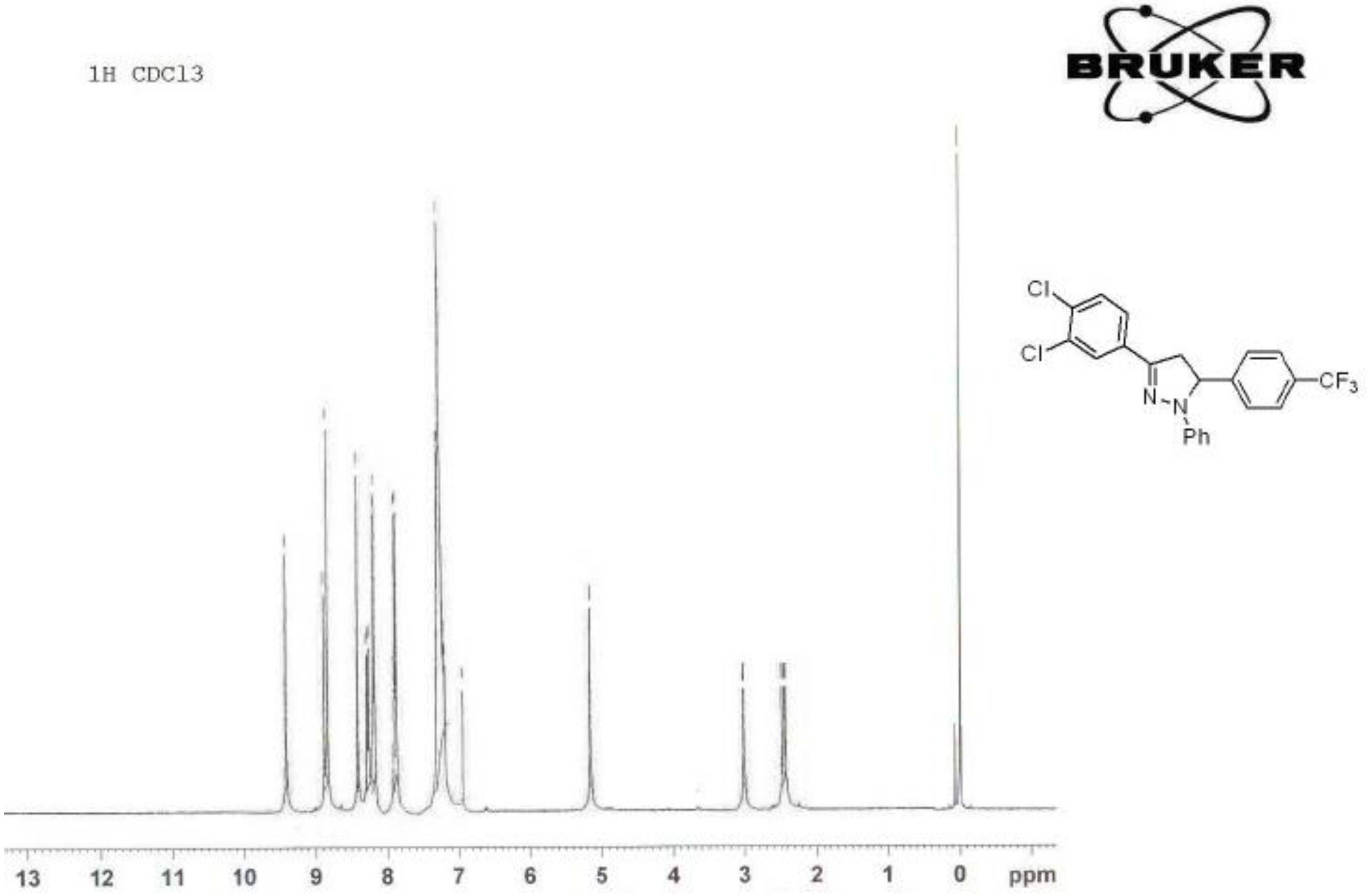

^{1}H-NMR spectrum of 3-(3,4-dichlorophenyl)-1-phenyl-5-(4-trifluoromethylphenyl)-4,5-dihydro-1H-pyrazole (**24**).

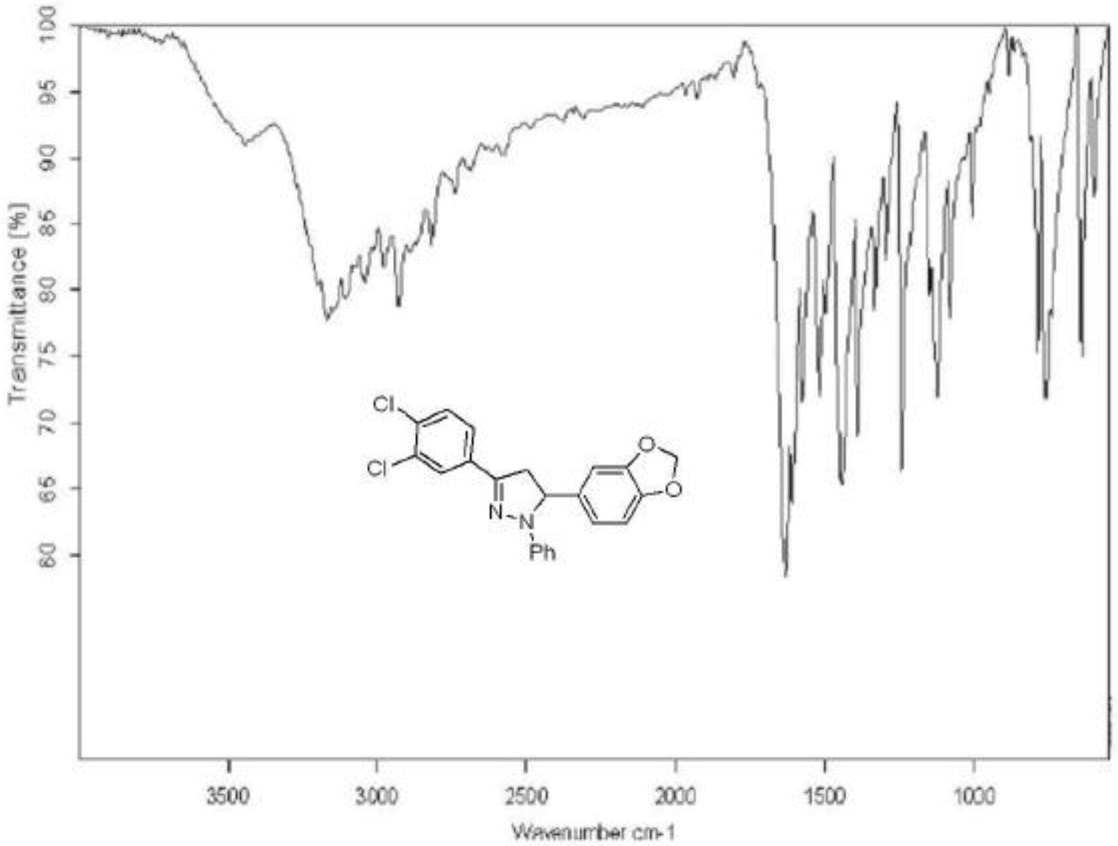

IR spectrum of 3-(3,4-dichlorophenyl)-1-phenyl-5-(benzo[d][1,3]dioxol-5-yl)-4,5-dihydro-1H-pyrazole (**25**).

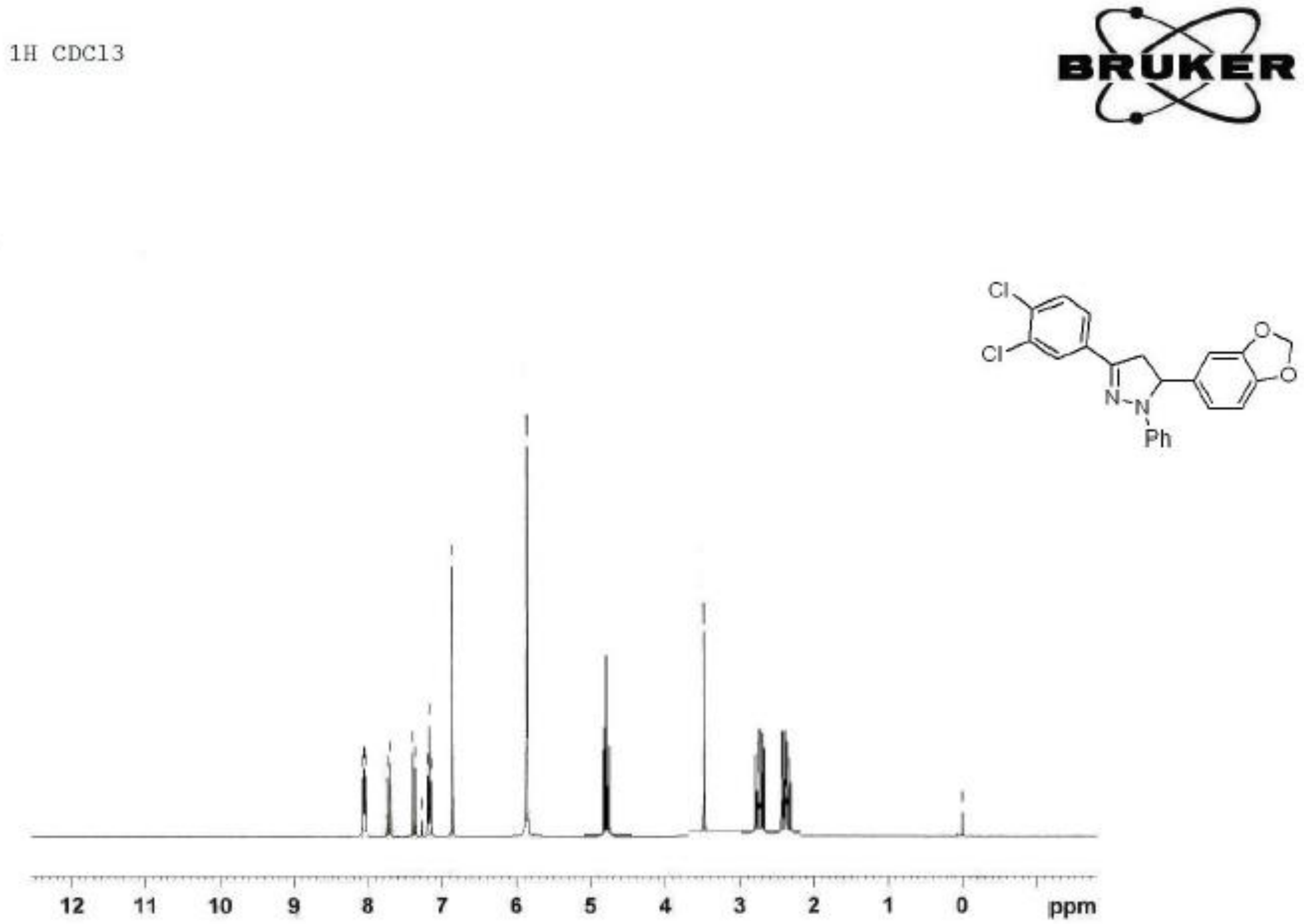

^{1}H-NMR spectrum of 3-(3,4-dichlorophenyl)-1-phenyl-5-(benzo[d][1,3]dioxol-5-yl)-4,5-dihydro-1H-pyrazole (**25**).

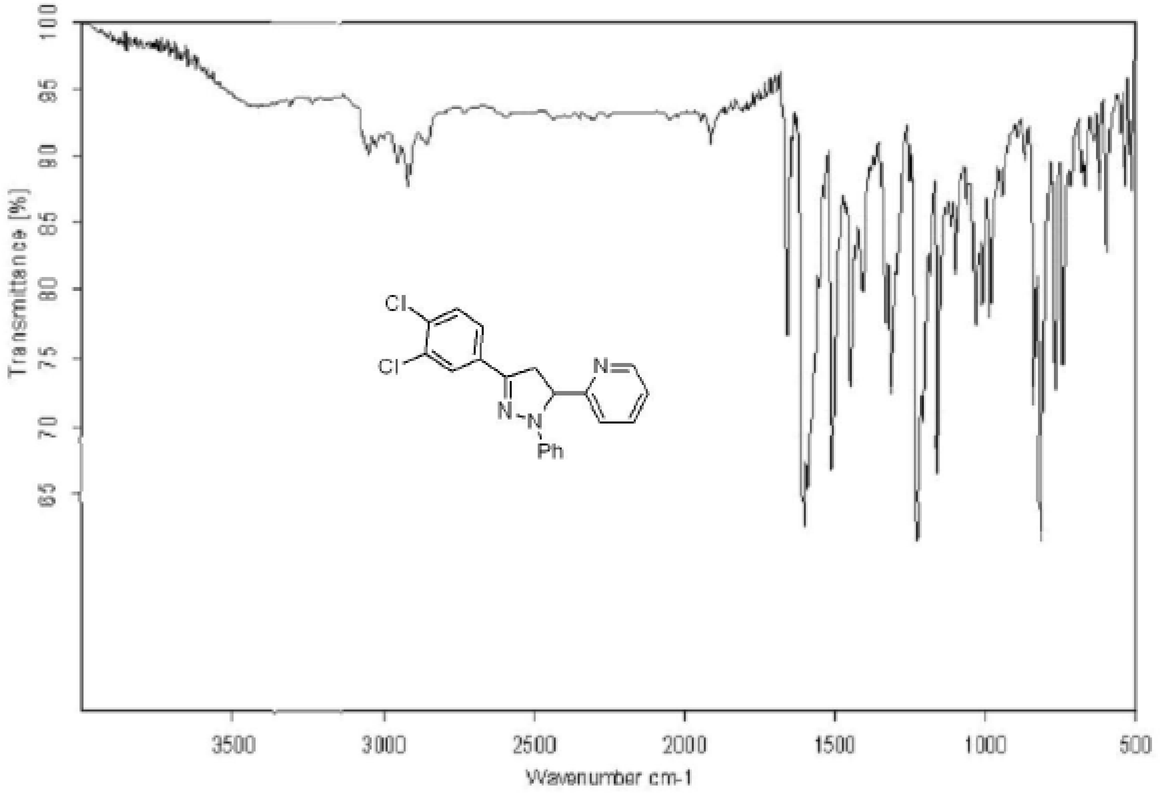

IR spectrum of 3-(3,4-dichlorophenyl)-1-phenyl-5-(pyridin-2-yl)-4,5-dihydro-1H-pyrazole (**26**).

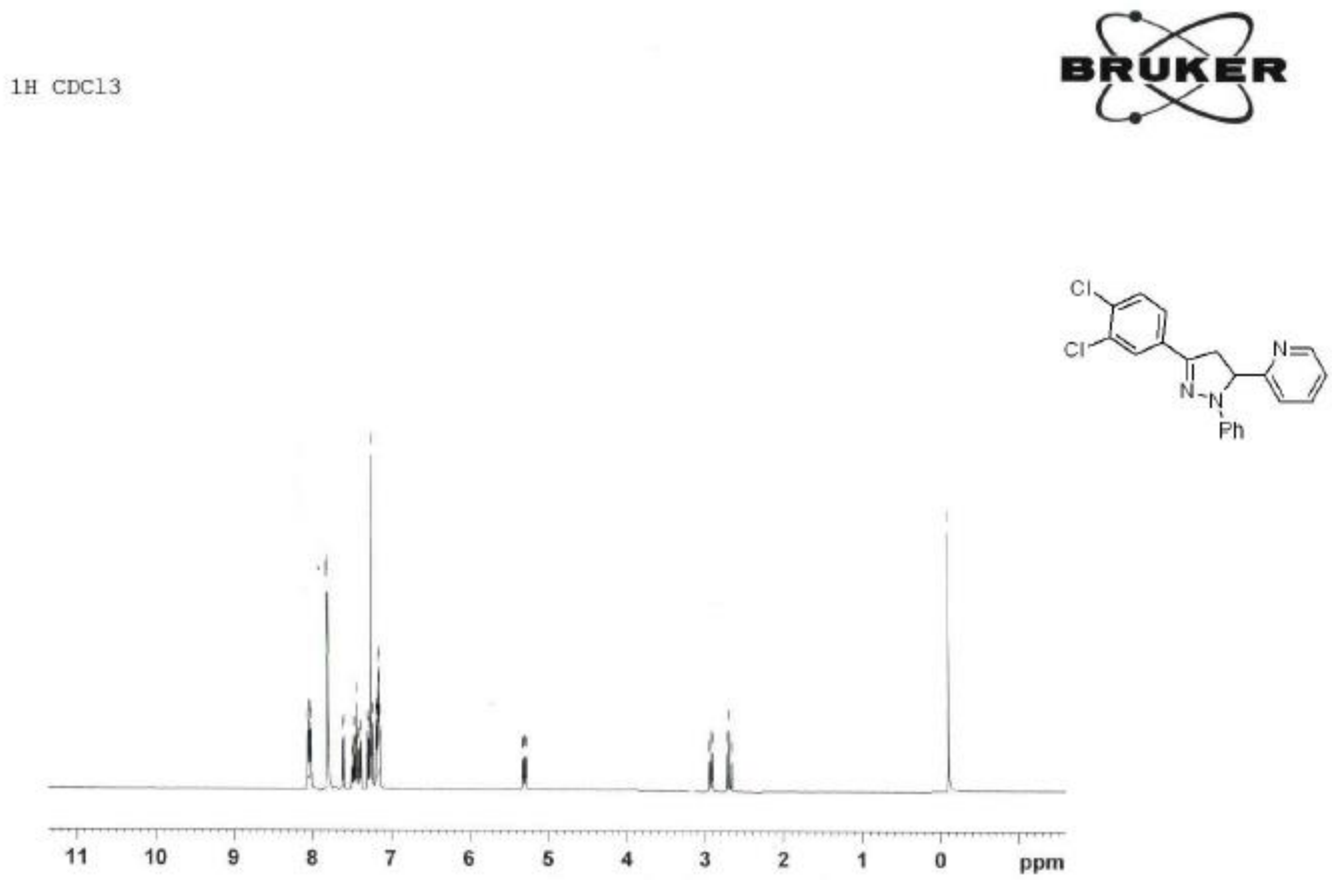

^{1}H-NMR spectrum of 3-(3,4-dichlorophenyl)-1-phenyl-5-(pyridin-2-yl)-4,5-dihydro-1H-pyrazole (**26**).

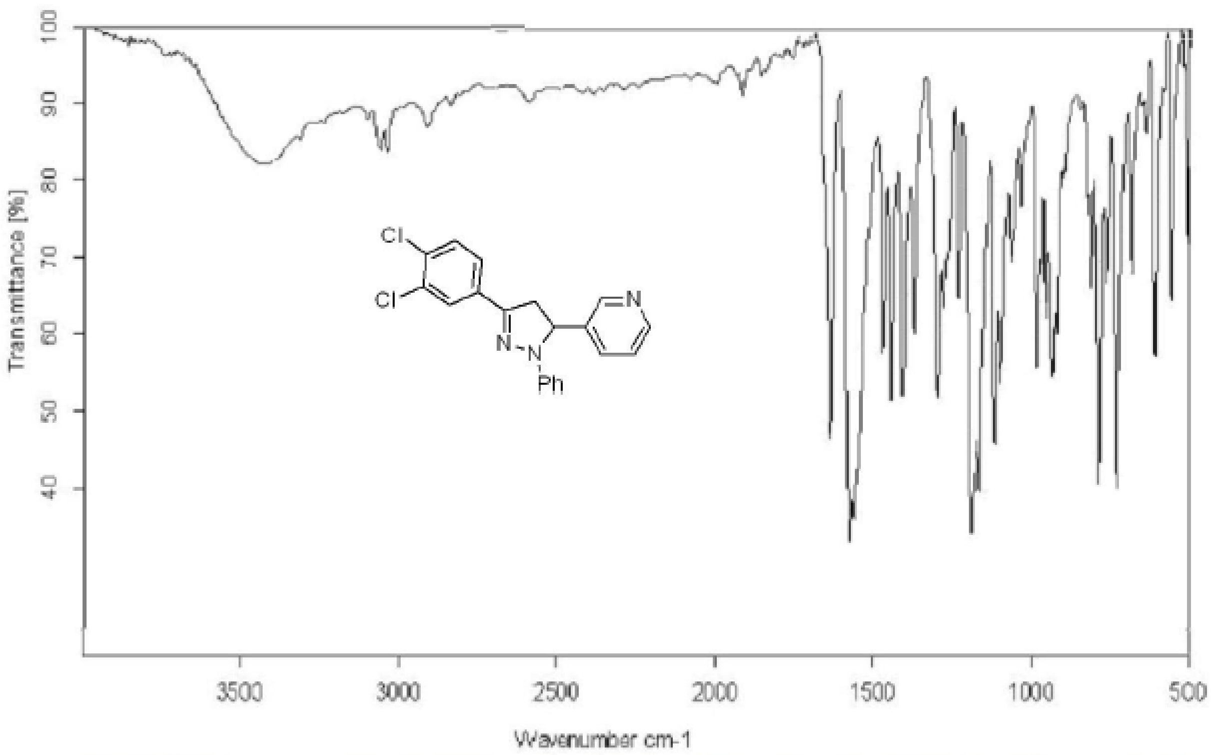

IR spectrum of 3-(3,4-dichlorophenyl)-1-phenyl-5-(pyridin-3-yl)-4,5-dihydro-1H-pyrazole (**27**).

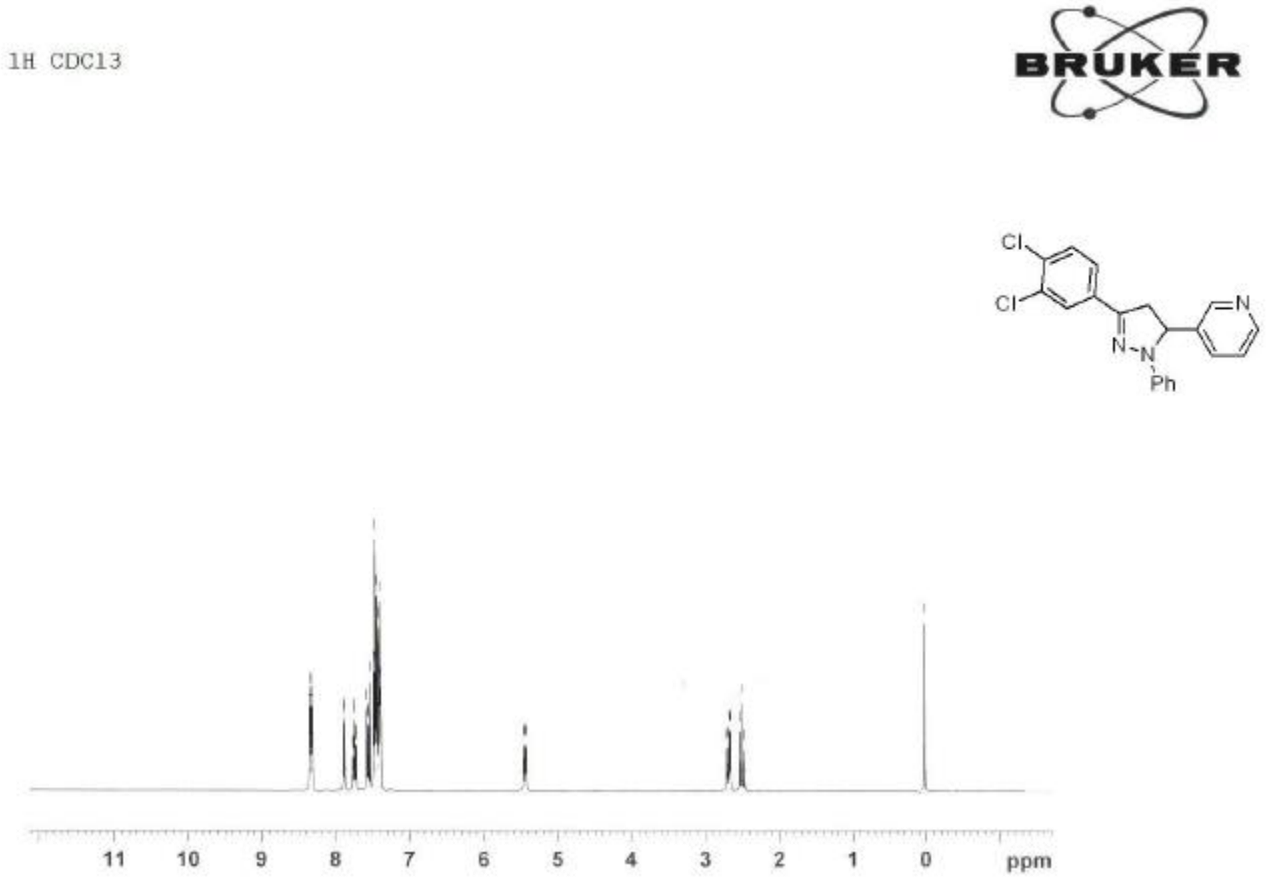

^{1}H-NMR spectrum of 3-(3,4-dichlorophenyl)-1-phenyl-5-(pyridin-3-yl)-4,5-dihydro-1H-pyrazole (**27**).

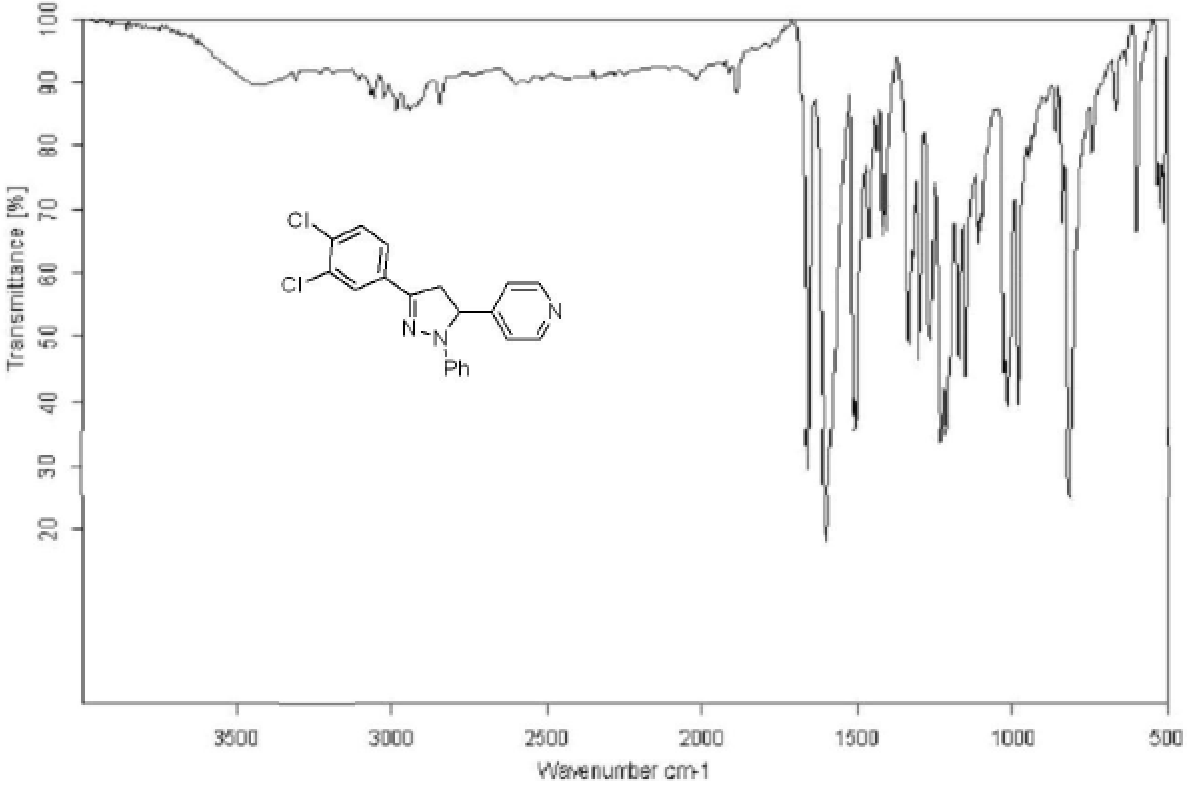

IR spectrum of 3-(3,4-dichlorophenyl)-1-phenyl-5-(pyridin-4-yl)-4,5-dihydro-1H-pyrazole (**28**).

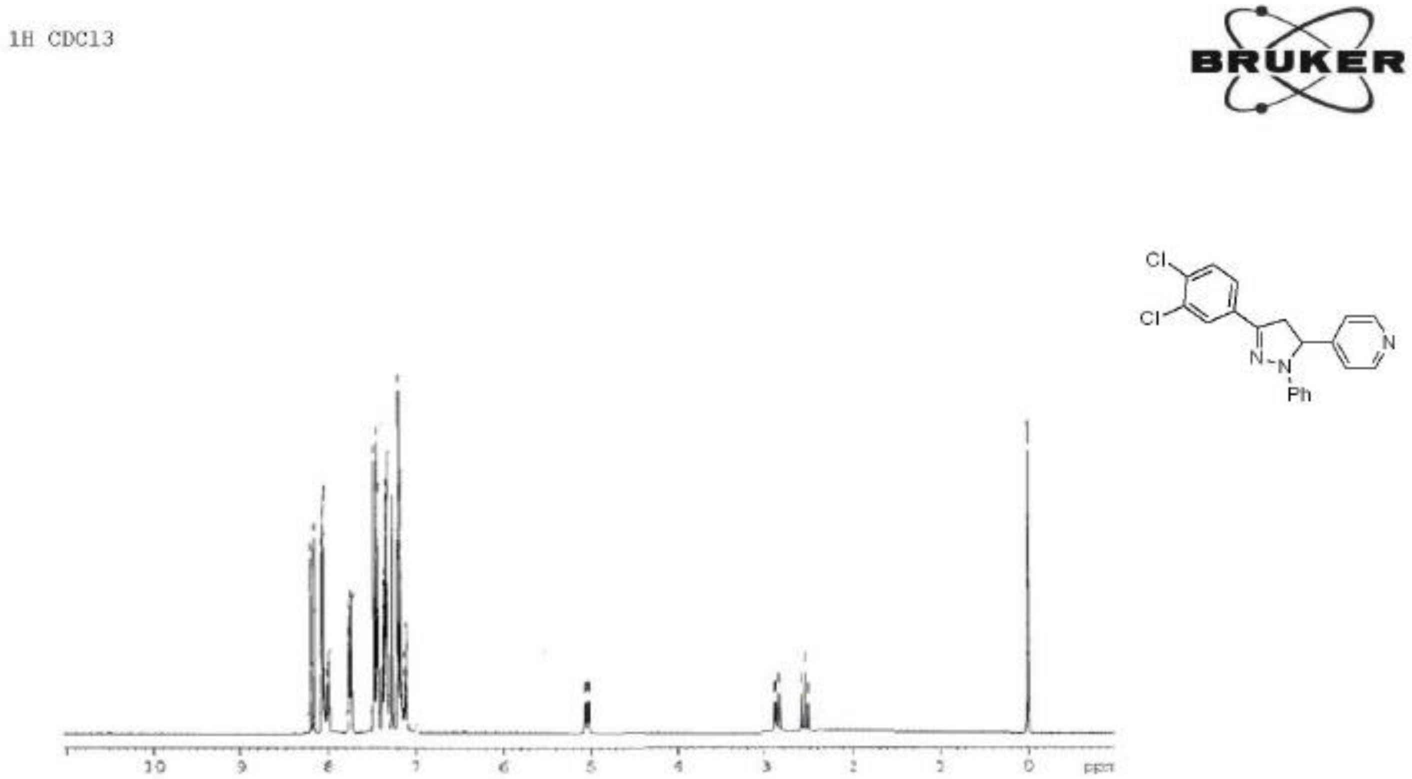

^{1}H-NMR spectrum of 3-(3,4-dichlorophenyl)-1-phenyl-5-(pyridin-4-yl)-4,5-dihydro-1H-pyrazole (**28**).

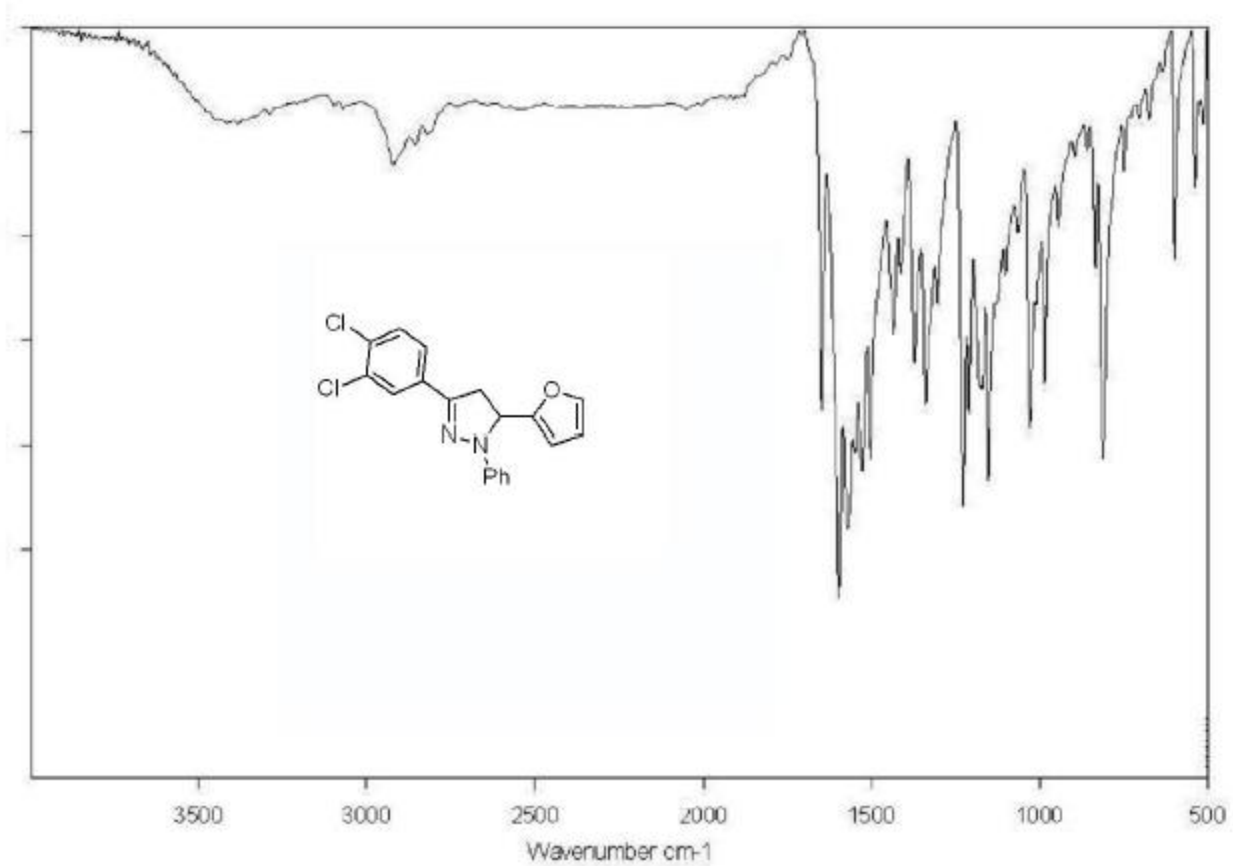

IR spectrum of 3-(3,4-dichlorophenyl)-1-phenyl-5-(furan-2-yl)-4,5-dihydro-1H-pyrazole (**29**).

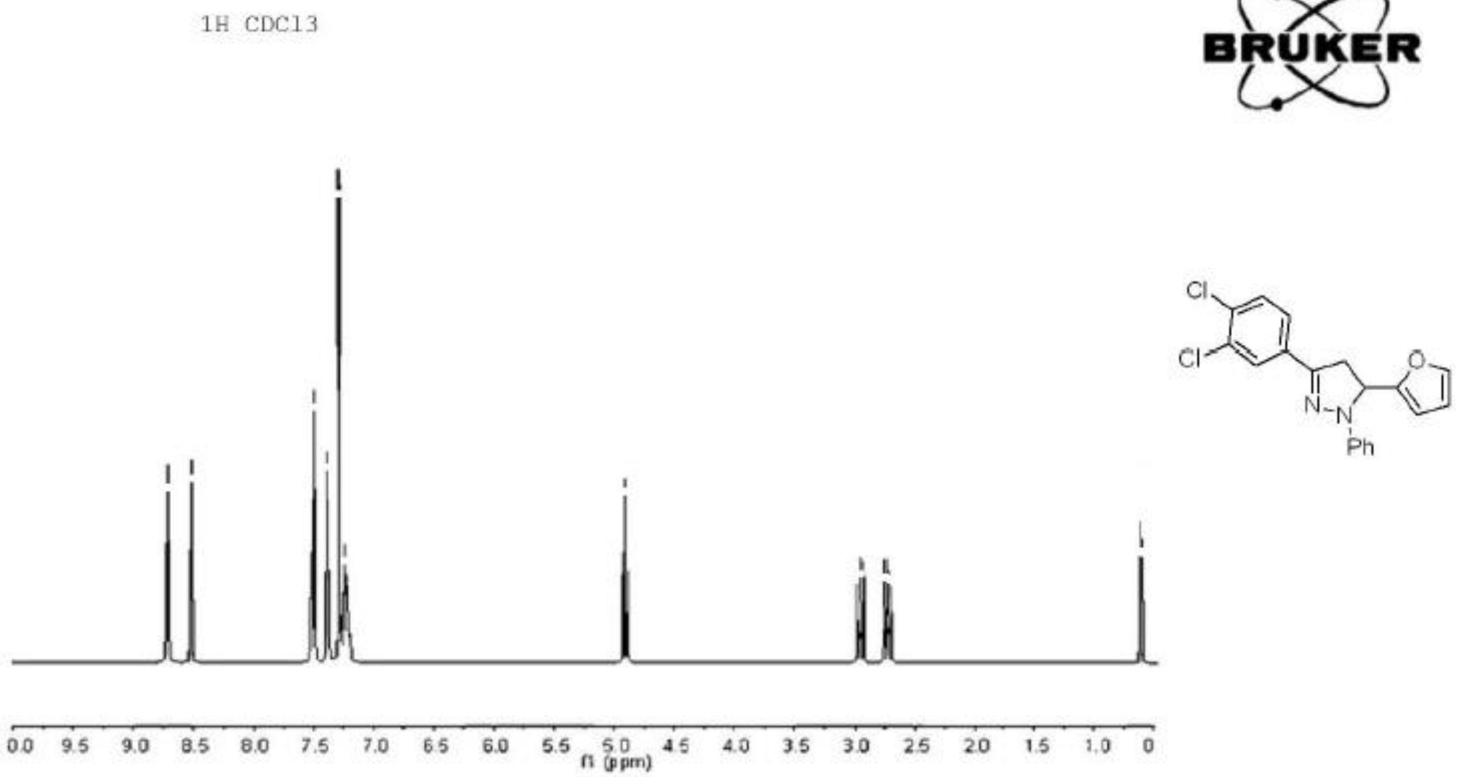

^{1}H-NMR spectrum of 3-(3,4-dichlorophenyl)-1-phenyl-5-(furan-2-yl)-4,5-dihydro-1H-pyrazole (**29**).

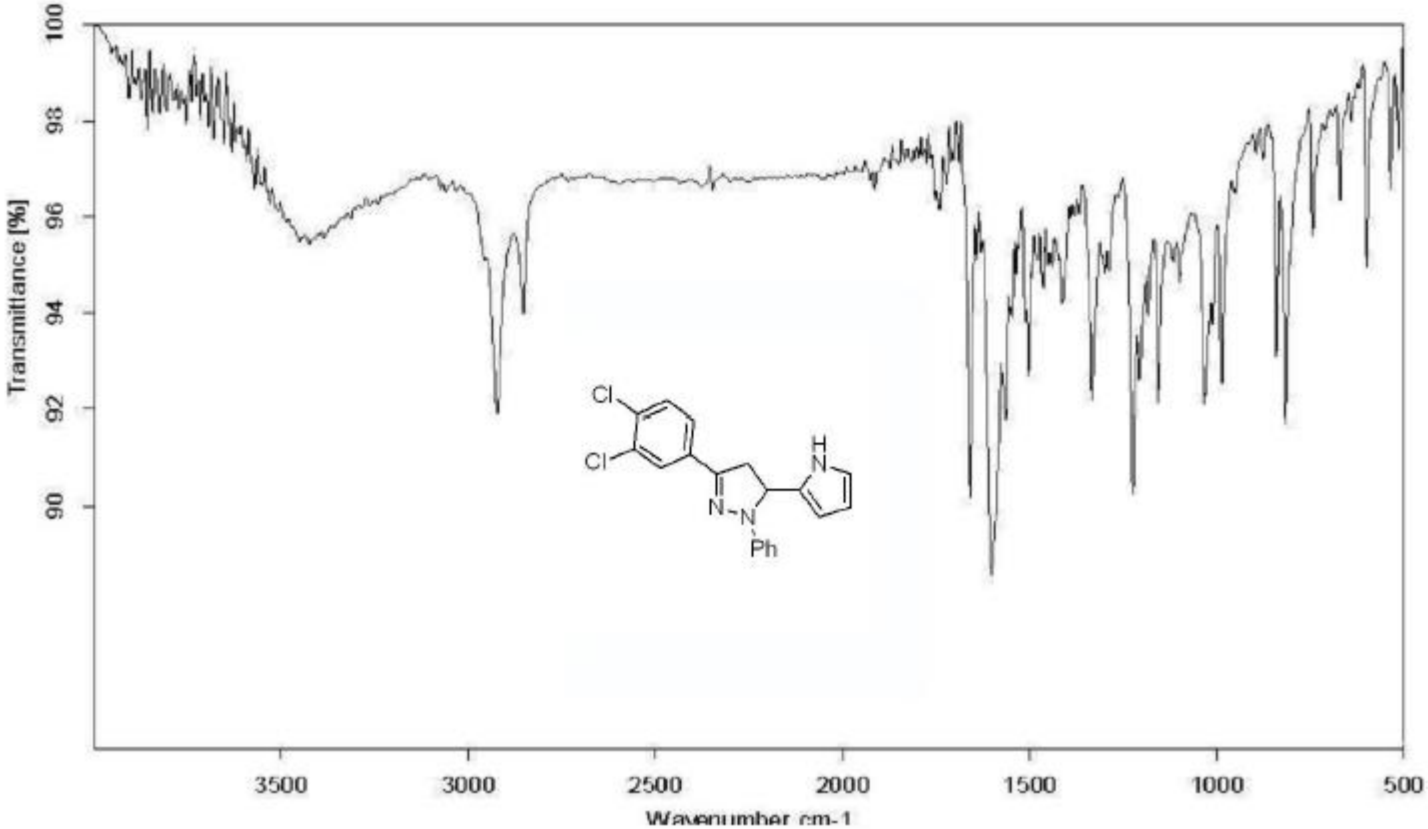

IR spectrum of 3-(3,4-dichlorophenyl)-1-phenyl-5-(1H-pyrrol-2-yl)-4,5-dihydro-1H-pyrazole (**30**).

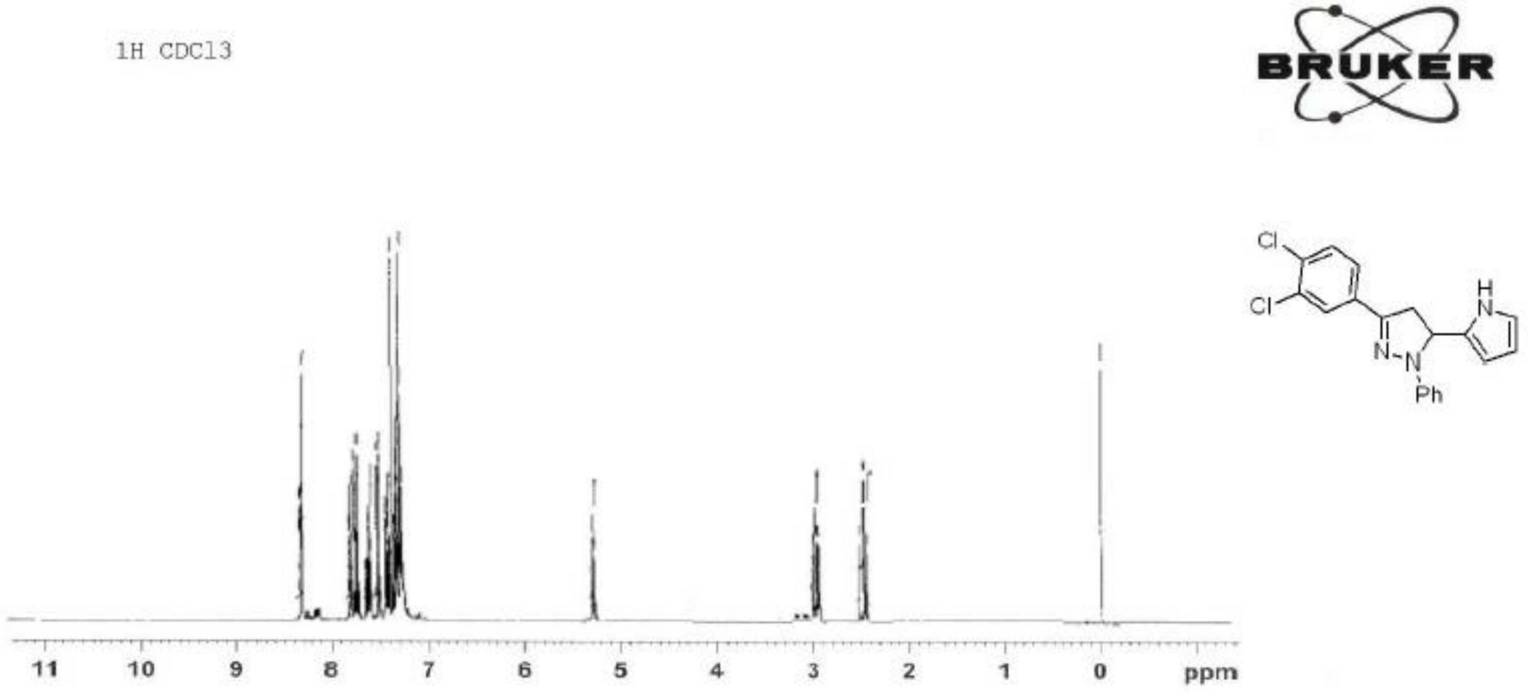

^{1}H-NMR spectrum of 3-(3,4-dichlorophenyl)-1-phenyl-5-(1H-pyrrol-2-yl)-4,5-dihydro-1H-pyrazole (**30**).

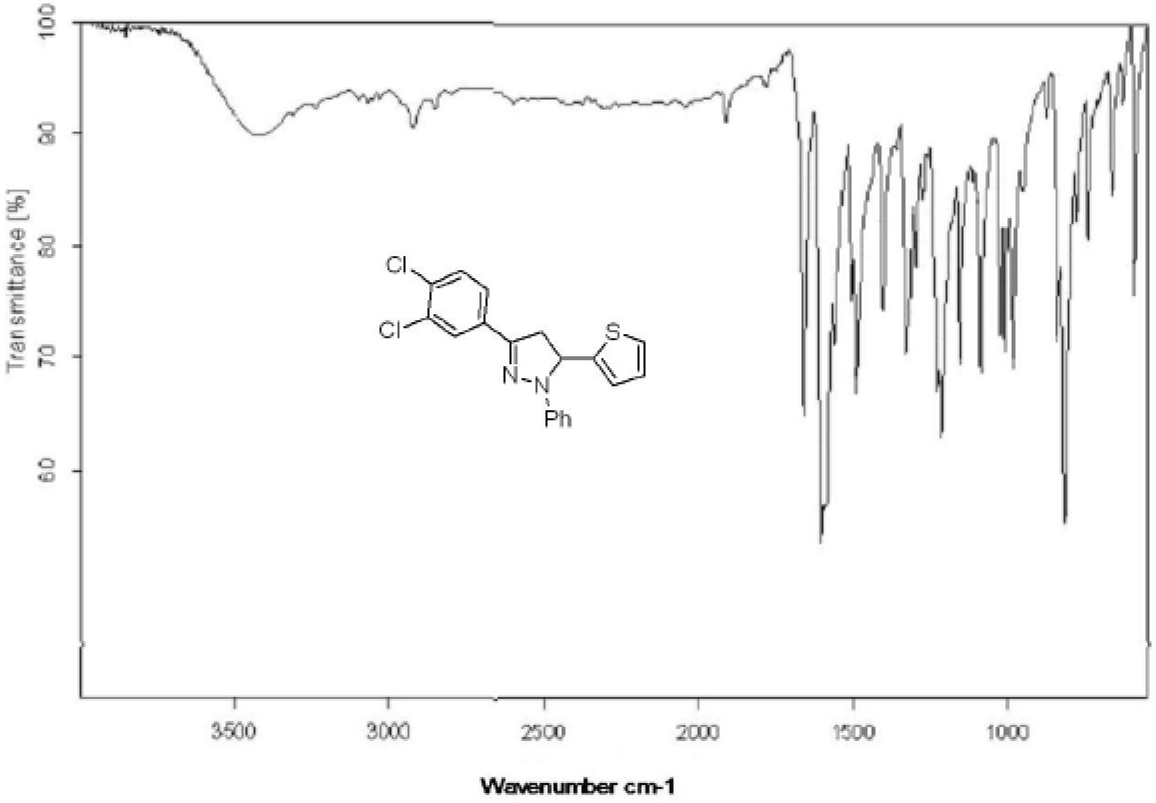

IR spectrum of 3-(3,4-dichlorophenyl)-1-phenyl-5-(thiophen-2-yl)-4,5-dihydro-1H-pyrazole (**31**).

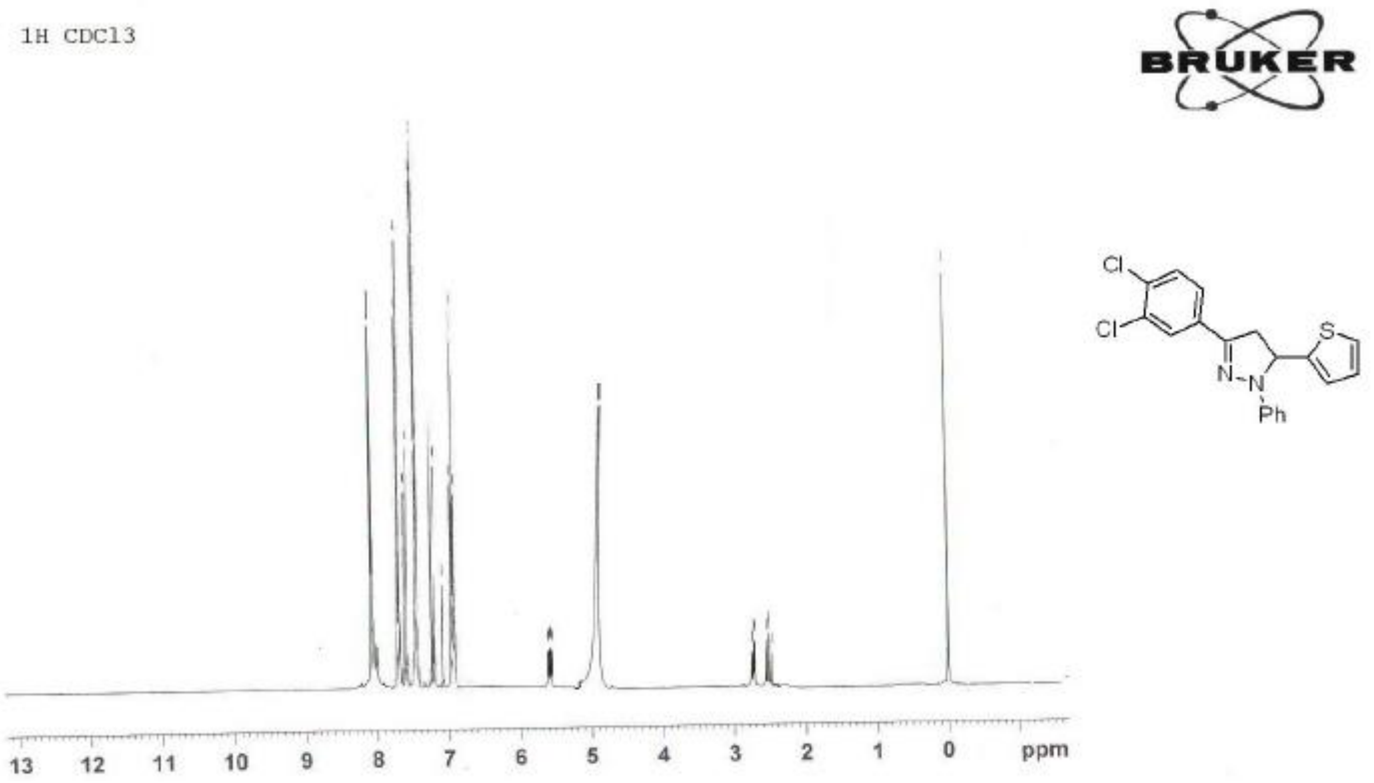

^{1}H-NMR spectrum of 3-(3,4-dichlorophenyl)-1-phenyl-5-(thiophen-2-yl)-4,5-dihydro-1H-pyrazole (**31**).

Rf values of chalcones and dihydropyrazoles

**Chalcones and dihydropyrazole TLC Profiles (20% ethyl acetate in hexane)**	
**Compound No.**	***Rf* value**
**7**	0.70
**8**	0.66
**9**	0.69
**11**	0.53
**12**	0.55
**14**	0.62
**15**	0.56
**16**	0.67
**17**	0.46
**18**	0.49
**19**	0.50
**20**	0.58
**21**	0.59
**22**	0.57
**23**	0.61
**24**	0.64
**25**	0.44
**26**	0.35
**27**	0.31
**28**	0.38
**29**	0.51
**30**	0.39
**31**	0.52
